# Probabilistic weather forecasting with machine learning

**DOI:** 10.1038/s41586-024-08252-9

**Published:** 2024-12-04

**Authors:** Ilan Price, Alvaro Sanchez-Gonzalez, Ferran Alet, Tom R. Andersson, Andrew El-Kadi, Dominic Masters, Timo Ewalds, Jacklynn Stott, Shakir Mohamed, Peter Battaglia, Remi Lam, Matthew Willson

**Affiliations:** Google DeepMind, London, UK

**Keywords:** Atmospheric dynamics, Natural hazards, Computer science

## Abstract

Weather forecasts are fundamentally uncertain, so predicting the range of probable weather scenarios is crucial for important decisions, from warning the public about hazardous weather to planning renewable energy use. Traditionally, weather forecasts have been based on numerical weather prediction (NWP)^1^, which relies on physics-based simulations of the atmosphere. Recent advances in machine learning (ML)-based weather prediction (MLWP) have produced ML-based models with less forecast error than single NWP simulations^2,3^. However, these advances have focused primarily on single, deterministic forecasts that fail to represent uncertainty and estimate risk. Overall, MLWP has remained less accurate and reliable than state-of-the-art NWP ensemble forecasts. Here we introduce GenCast, a probabilistic weather model with greater skill and speed than the top operational medium-range weather forecast in the world, ENS, the ensemble forecast of the European Centre for Medium-Range Weather Forecasts^4^. GenCast is an ML weather prediction method, trained on decades of reanalysis data. GenCast generates an ensemble of stochastic 15-day global forecasts, at 12-h steps and 0.25° latitude–longitude resolution, for more than 80 surface and atmospheric variables, in 8 min. It has greater skill than ENS on 97.2% of 1,320 targets we evaluated and better predicts extreme weather, tropical cyclone tracks and wind power production. This work helps open the next chapter in operational weather forecasting, in which crucial weather-dependent decisions are made more accurately and efficiently.

## Abstract (web summary)

GenCast, a probabilistic weather model using artificial intelligence for weather forecasting, has greater skill and speed than the top operational medium-range weather forecast in the world and provides probabilistic, rather than deterministic, forecasts.

## Main

Every day, people, governments and other organizations around the world rely on accurate weather forecasts to make many key decisions—whether to carry an umbrella, when to flee an approaching tropical cyclone, how to plan the use of renewable energy in a power grid, or how to prepare for a heatwave. But forecasts will always have some uncertainty, because we can only partially observe the current weather, and even our best weather models are imperfect. The highly non-linear physics of weather means that small initial uncertainties and errors can rapidly grow into large uncertainties about the future^5^. Making important decisions often requires knowing not just a single probable scenario but the range of possible scenarios and how likely they are to occur.

Traditional weather forecasting is based on numerical weather prediction (NWP) algorithms, which approximately solve the equations that model atmospheric dynamics. Deterministic NWP methods map the current estimate of the weather to a forecast of how the future weather will unfold over time. To model the probability distribution of different future weather scenarios^6,7^, weather agencies increasingly rely on ensemble forecasts, which generate several NWP-based forecasts, each of which models a single possible scenario^4,8–11^. ENS of the European Centre for Medium-Range Weather Forecasting (ECMWF)^4^ is the state-of-the-art NWP-based ensemble forecast in the broader Integrated Forecast System of the ECMWF and will subsume their deterministic forecast, HRES, going forward^12^.

ENS satisfies several key desiderata of a probabilistic weather model. First, its ensemble members represent sharp and spectrally realistic individual weather trajectories, as opposed to some summary statistic such as a conditional mean. Second, it produces skilful and calibrated marginal forecast distributions (forecasts of the weather at a given place and time), which is important for many day-to-day users of weather forecasts. Third, it captures the aspects of the joint spatiotemporal structure of the forecast distribution that are crucial for probabilistic modelling of large-scale phenomena such as cyclones and for applications such as forecasting distributed energy generation. Nonetheless, ENS—along with other NWP-based ensemble forecasts—is still prone to errors, is slow to run and is time-consuming to engineer.

Recent advances in machine learning (ML)-based weather prediction (MLWP) have been shown to provide greater accuracy and efficiency than NWP for non-probabilistic forecasts^2,3,13–18^. Rather than forecasting a single weather trajectory, or a distribution of trajectories, these methods have largely focused on forecasting the mean of the probable trajectories, with relatively little emphasis on quantifying the uncertainty associated with a forecast. They are typically trained to minimize the mean squared error (MSE) of their predictions and as a result tend to produce blurry forecasts, especially at longer lead times, rather than a specific realization of a possible weather state^2^. There have been limited attempts to use traditional initial condition perturbation methods to produce ensembles with MLWP-based forecasts^3,15,18,19^. However, these methods have not addressed the issue of blurring—meaning that their ensemble members do not represent realistic samples from the weather distribution—and they have not rivalled operational ensemble forecasts such as ENS. An exception is NeuralGCM^20^, a hybrid NWP–MLWP method, which combines the dynamical core of a traditional NWP with local ML-based parameterizations and shows competitive performance with operational ensemble forecasts. However, ensembles of this hybrid model have 1.4° spatial resolution, which is an order of magnitude coarser than operational NWP-based forecasts.

This work presents GenCast, the first MLWP method, to our knowledge, that significantly outperforms the top operational ensemble NWP model, ENS. We demonstrate that GenCast generates ensembles of realistic individual weather trajectories, providing both better marginal and better joint forecast distributions than ENS.

## GenCast

GenCast is a probabilistic weather model that generates global 15-day ensemble forecasts at 0.25° resolution, which are more accurate than the top operational ensemble system, ENS of ECMWF. Generating a single 15-day GenCast forecast takes about 8 min on a Cloud TPUv5 device, and an ensemble of forecasts can be generated in parallel.

GenCast models the conditional probability distribution *P*(**X**^*t*+1^|**X**^*t*^, **X**^*t*−1^) of the future weather state **X**^*t*+1^ conditional on the current and previous weather states. A forecast trajectory **X**^1:*T*^ of length *T* is modelled by conditioning on the initial and previous states, (**X**^0^, **X**^−1^), and factoring the joint distribution over successive states,

$$P({{\bf{X}}}^{1:T}| {{\bf{X}}}^{0},{{\bf{X}}}^{-1})=\mathop{\prod }\limits_{t=0}^{T-1}P({{\bf{X}}}^{t+1}| {{\bf{X}}}^{t},{{\bf{X}}}^{t-1})$$

each of which is sampled autoregressively.

The representation of the global weather state, **X**, consists of six surface variables and six atmospheric variables at 13 vertical pressure levels (Extended Data Table 1) on an equiangular 0.25° latitude–longitude grid. The forecast horizon is 15 days, with 12 h between successive steps *t* and *t* + 1, so *T* = 30. We train GenCast using analysis for **X**, which represents the best estimate of the weather state, inferred from observations.

**Fig. 1:**
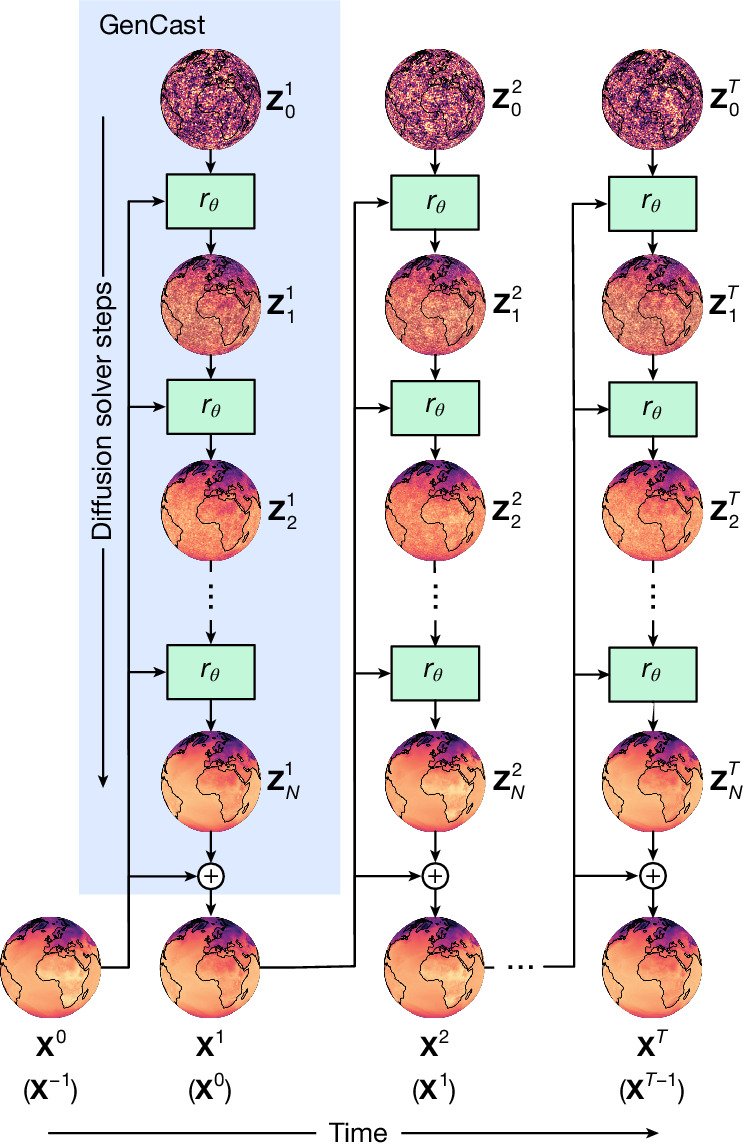
Schematic of how GenCast produces a forecast. The blue box shows how, conditioning on inputs (**X**^0^, **X**^−1^), an initial noise sample, ${{\bf{Z}}}_{0}^{1}$, is refined by the neural network refinement function, *r*_*θ*_ (green box), which is parameterized by *θ*. The resulting ${{\bf{Z}}}_{1}^{1}$ is the first refined candidate state, and this process repeats *N* times. The final ${{\bf{Z}}}_{N}^{1}$ is then added as a residual to **X**^0^ to produce the weather state at the next time step, **X**^1^. This process then repeats autoregressively, *T* = 30 times, conditioning on (**X**^*t*^, **X**^*t*−1^) and using a new initial noise sample ${{\bf{Z}}}_{0}^{t}$ at each step to produce the full weather trajectory sample (for visual clarity, we illustrate the previous state in parentheses, (**X**^*t*−1^), below the current state, **X**^*t*^, but note that it is not added to ${{\bf{Z}}}_{N}^{t}$ as a residual for predicting **X**^*t*+1^). Each trajectory generated by independent ${{\bf{Z}}}_{0}^{1:T}$ noise samples represents a sample from, *P*(**X**^1:*T*^|**X**^0^, **X**^−1^).

GenCast is implemented as a conditional diffusion model^21–23^, a generative ML method that can model the probability distribution of complex data and generate new samples. Diffusion models underpin many of the recent advances in modelling natural images, sounds and videos under the umbrella of generative AI^24,25^. Diffusion models work through a process of iterative refinement. A future atmospheric state, **X**^*t*+1^, is produced by iteratively refining a candidate state initialized as pure noise, ${{\bf{Z}}}_{0}^{t+1}$, conditioned on the previous two atmospheric states (**X**^*t*^, **X**^*t*−1^). The blue box in Fig. 1 shows how the first forecast step is generated from the initial conditions and how the full trajectory, **X**^1:*T*^, is generated autoregressively. Because each time step in a forecast is initialized with noise (${{\bf{Z}}}_{0}^{t+1}$), the process can be repeated with different noise samples to generate an ensemble of trajectories. See Methods for further details of the sampling process.

At each stage of the iterative refinement process, GenCast makes use of a denoiser neural network, which is trained to remove noise artificially added to atmospheric states using the loss function described in the Methods. The architecture of the denoiser comprises an encoder, processor and decoder. The encoder component maps a noisy target state ${{\bf{Z}}}_{n}^{t+1}$, as well as the conditioning (**X**^*t*^, **X**^*t*−1^), from the equiangular 0.25° latitude–longitude grid to an internal learned representation defined on a six-times-refined icosahedral mesh. The processor component is a graph transformer^26^ in which each node attends to its *k*-hop neighbourhood on the mesh. The decoder component maps from the internal mesh representation back to a denoised target state, defined on the grid.

GenCast is trained on 40 years of best-estimate analysis from 1979 to 2018, taken from the publicly available ERA5 (fifth generation ECMWF reanalysis) reanalysis dataset^27^. Reanalysis provides a reconstruction of past weather by computing analysis for historical dates and times. For simplicity, we refer to ERA5 reanalysis as analysis from here on. Full details of the GenCast architecture and training protocol are provided in the Methods. When evaluating GenCast, we initialize it with ERA5 analysis.

**Fig. 2:**
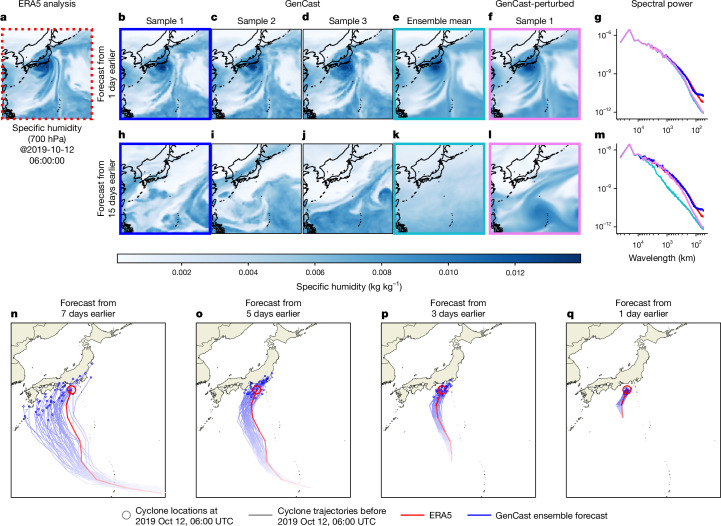
Visualization of forecasts and tropical cyclone tracks. **a**, The ERA5 analysis state^27^ for specific humidity at 700 hPa, at validity time 06 UTC, 12 October 2019, shows Typhoon Hagibis near the centre of the frame, hours before making landfall in Japan. **b**–**d**, Sample 1 (**b**), sample 2 (**c**) and sample 3 (**d**) GenCast forecast states, initialized one day earlier, show how the samples are sharp and very similar to one another. **e**, The GenCast ensemble mean, obtained by computing the mean of 50 sample states such as in **b**–**d**, is somewhat blurry, showing how uncertainty results in a blurrier average state. **f**, Sample 1 forecast state from GenCast-Perturbed, initialized one day earlier as in **b**–**e**, is blurry, similar to a single-step ensemble mean. **g**, The spatial power spectrum of the states in **a**, **b**, **e** and **f**, in which the line colours match the frames of the panels, show how spectra of the GenCast samples closely match with that of ERA5, whereas the blurrier GenCast ensemble mean and GenCast-Perturbed states have less power at shorter wavelengths. **h–m**, These subplots are analogous to **b**–**g**, except the forecasts are initialized 15 days earlier. The GenCast samples are still sharp (**h**–**j**) and GenCast-Perturbed (**l**) is still equally blurry, whereas the GenCast ensemble mean (**k**) is even blurrier than at 1-day lead time. This is also reflected in the power spectrum (**m**). **n**–**q**, The trajectory of Typhoon Hagibis based on ERA5^27^ (in red) and the ensemble of tropical cyclone trajectories from GenCast (in blue) up to a validity time 4 h before the cyclone made landfall in Japan. GenCast forecasts are shown at lead times of 7 days, 3 days, 5 days and 1 day. The blue and red circles show cyclone locations at the validity time. At long lead times, the cyclone trajectories have a substantial spread, whereas for the shorter lead times, the predictive uncertainty collapses to a small range of trajectories. Typhoon Hagibis represents the 55^th^ percentile of GenCast’s ensemble mean position error among tropical cyclones in 2019.

As an illustrative example, Fig. 2b–d,h–j showcases GenCast forecast samples and Fig. 2n–q provides an example of how they can be used in important downstream applications, such as predicting the paths of tropical cyclones. Typhoon Hagibis—the costliest tropical cyclone of 2019—is shown as a representative case study. When initialized 7 days before the landfall of Typhoon Hagibis, the predicted trajectories of GenCast exhibit high uncertainty, covering a wide range of possible scenarios. At shorter lead times, the uncertainty of GenCast about the path of the cyclone is lower, reflecting greater confidence about the landfall timing and location.

## Baselines

We compare GenCast to ENS, currently the best operational ensemble forecast, which we regridded from its (pre-June 2023) native 0.2° latitude–longitude resolution to 0.25°. ENS contains 50 perturbed ensemble members, so we used 50-member GenCast ensembles to perform all evaluations. The public TIGGE archive^28^ only makes all 50 ENS ensemble members available for surface variables and for atmospheric variables at eight pressure levels in the troposphere. So these are the variables and levels we compare models on.

We also develop a deterministic 12 h step forecast model using the GenCast architecture, to serve as a strong ML baseline and an ablation of the role of diffusion. We used this model to generate ensemble forecasts (denoted as GenCast-Perturbed) by initializing it using ERA5 analysis perturbed by Gaussian Process noise; full details are in Supplementary Information section A.4.

For a fair comparison of models, we evaluate each model against its corresponding best-estimate analysis, following established practice^2,29^. We thus evaluate the operational forecasts of ECMWF against HRES-fc0^30^ (a dataset comprising the initial conditions used for the HRES deterministic forecast of ECMWF), and we evaluate ML models that were trained and initialized using ERA5, against ERA5^30^.

We use 2019 as our test period, and, following the protocol in ref. ^2^, we initialize ML models using ERA5 at 06 UTC and 18 UTC, as these benefit from only 3 h of look-ahead (with the exception of sea surface temperature, which in ERA5 is updated once per 24 h). This ensures ML models are not afforded an unfair advantage by initializing from states with longer look-ahead windows.

We follow a standard verification practice^29^ in evaluating ensemble forecasts using best-estimate analysis as ground truth. However, we note that this does not reward representing initial condition uncertainty. We also note that we evaluate the raw output of GenCast against that of ENS, following standard practice in the field. Both MLWP and NWP forecasts can be further improved by post-processing methods, and the relative impact of these methods on the two approaches is an interesting direction for future work.

## Realism of GenCast samples

Figure 2 shows some of the forecast samples of GenCast for Typhoon Hagibis, shortly before it made landfall in Japan on 12 October 2019. Figure 2b–e,g,h–k,m shows that GenCast forecasts are sharp and have spherical harmonic power spectra that closely match the ERA5 ground truth at both 1- and 15-day lead times. This reflects how the ensemble members of GenCast, like those of ENS, represent realistic samples of the weather. As expected, the GenCast ensemble mean is blurry, losing power at high frequencies (see also Supplementary Figs. B5 and B6). Forecasts by deterministic models trained to minimize forecast MSE—including top deterministic MLWP models such as GraphCast^2^—are blurred and closer to the ensemble mean^2^. Ensemble members generated by perturbing these deterministic models also blur. This is especially true for multi-step-trained models such as GraphCast, but it is also true (albeit to a lesser extent) for models such as GenCast-Perturbed (Fig. 2f,l), which are only trained to predict a one-step forecast-distribution mean.

## Skilful marginal forecast distributions

Many day-to-day users of weather forecasts rely on the spatiotemporal marginals of the forecast distributions, that is, the weather forecast for a given place and time. We evaluate the per-grid-cell marginals of GenCast and ENS in terms of overall forecast skill, calibration and performance on extreme weather prediction.

### Ensemble skill

**Fig. 3:**
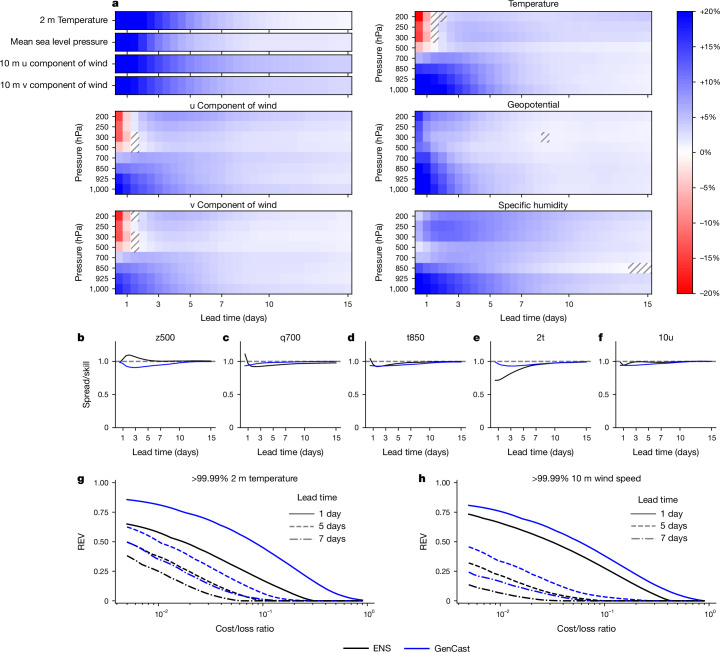
The marginal forecast distributions of GenCast are skilful and well-calibrated. **a**, CRPS scores for GenCast versus ENS^4^ in 2019. The scorecard compares CRPS skill between GenCast and ENS across all variables and eight pressure levels. Dark-blue cells on the scorecard indicate a variable, lead time and level combination for which GenCast has 20% better (that is, lower) CRPS than ENS, whereas dark-red cells indicate 20% lower CRPS for ENS (white means they perform equally). The results show that GenCast significantly (*P* < 0.05) outperforms ENS on 97.2% of all reported variable, lead time and level combinations. Hatched regions indicate neither model is significantly better. **b**–**f**, Spread/skill scores for GenCast and ENS for selected variables. Both models are generally well-calibrated with spread/skill close to 1. **g**,**h**, REV for predictions of the exceedance of the 99.99th percentile for 2 m temperature and 10 m wind speed, at lead times of 1 day, 5 days and 7 days. GenCast consistently achieves greater REV than ENS whenever either forecast is better than climatology, particularly at small cost/loss ratios.

The CRPS^31^ is a standard measure of the skill of a probabilistic forecast. It measures how well the marginal distributions of the forecast represent the ground truth, and it is minimized, in expectation, by a forecast whose marginals reflect true predictive uncertainty. See Supplementary Information section A.5.1 for the mathematical definition of CRPS. As shown in the scorecard of Fig. 3, the forecasts of GenCast are significantly more skilful (*P* < 0.05) than that of ENS on 97.2% of our 1,320 variable, lead time and vertical level combinations (and 99.6% of targets at lead times greater than 36 h). Although dependencies across weather variables mean that these 1,320 scorecard targets do not each represent independent forecast tasks, such scorecards are a standard means of summarizing model performance. The largest improvements of GenCast are often at shorter lead times up to around 3–5 days, for surface variables, as well as temperature and specific humidity at higher pressure levels, for which the CRPS skill scores range between 10% and 30% better. GenCast-Perturbed also achieves strong results, with better or competitive CRPS compared with ENS on 82% of scorecard targets (Supplementary Fig. B7) but is still definitively worse than that of GenCast, which outperforms the CRPS of GenCast-Perturbed in 99% of targets (Extended Data Fig. 8 and Supplementary Fig. B8). Owing to our lack of confidence in the quality of ERA5 precipitation data, we exclude precipitation results from our main results and refer readers to Supplementary Information section B.2.

**Extended Data Fig. 1:**
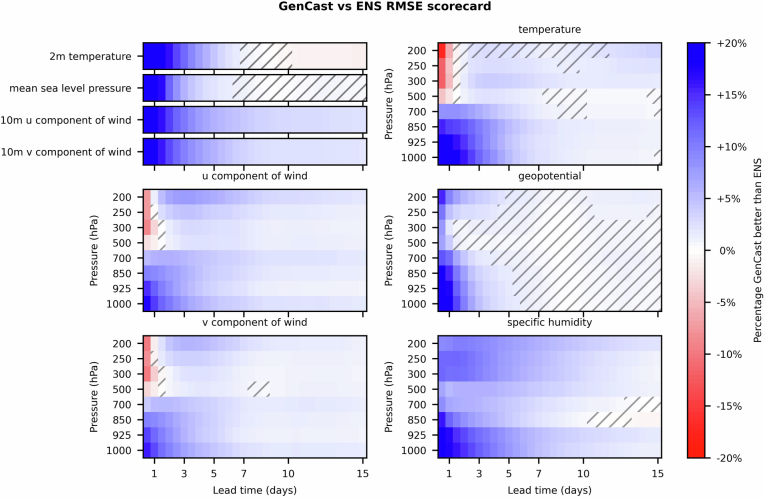
GenCast displays as good or better ensemble-mean RMSE than ENS. A scorecard comparing GenCast to ENS^4^. Dark blue cells on the scorecard indicate a variable, lead time, and level combination where GenCast has 20% better (i.e. lower) ensemble-mean RMSE than ENS, while dark red cells indicate 20% lower ensemble-mean RMSE for ENS (white means they perform equally). Hatched regions indicate where neither model is significantly better (*P* > 0.05). GenCast is as good or better than ENS on 96% of targets, and significantly better (*P* < 0.05) on 78% of targets.

We also compared the root mean squared error (RMSE) of the ensemble means of GenCast and ENS. The ensemble-mean RMSE measures how closely the mean of an ensemble of forecasts matches ground truth. Although RMSE is a common metric for deterministic forecasts, it does not account for uncertainty, which is central to probabilistic verification. Nonetheless, as shown in Extended Data Fig. 1, the ensemble mean RMSE of GenCast is as good or better than that of ENS on 96% of targets and significantly better (*P* < 0.05) on 78% of targets.

### Ensemble calibration

For a probabilistic forecast to be useful, it should be well-calibrated: it should know when it may be wrong and have confidence when it is likely to be right. This is a crucial aspect of the quality of the forecast distribution, allowing a decision-maker to hedge their choices in proportion to the confidence of the forecast. Two common tools in the weather community for evaluating calibration of the marginal forecast distributions, on average, are spread/skill ratios and rank histograms.

Well-calibrated probabilistic forecasts exhibit uncertainty (as measured by ensemble spread), which is commensurate on average with the size of their errors^32^. The degree to which this relationship holds can be quantified by the spread/skill ratio defined in Supplementary Information section A.5.3. This ratio should be 1 for a perfect ensemble forecast, with values greater than 1 suggestive of overdispersion (an underconfident forecast) and values less than 1 suggestive of underdispersion (overconfidence).

Similarly, the members of an ideal ensemble forecast should be indistinguishable from ground truth values. Deviations from this property on average can be diagnosed using rank histograms^33^. The rank histogram should be flat if the truth tends to be indistinguishable from the ensemble members, inverted U-shaped if the truth mostly ranks near the centre of the ensemble (indicating the ensembles are overdispersed), and U-shaped if the truth ranks mostly near the tails of the ensemble (indicating the ensembles are underdispersed). See Supplementary Information section A.5.4 for definitions and details.

**Extended Data Fig. 2:**
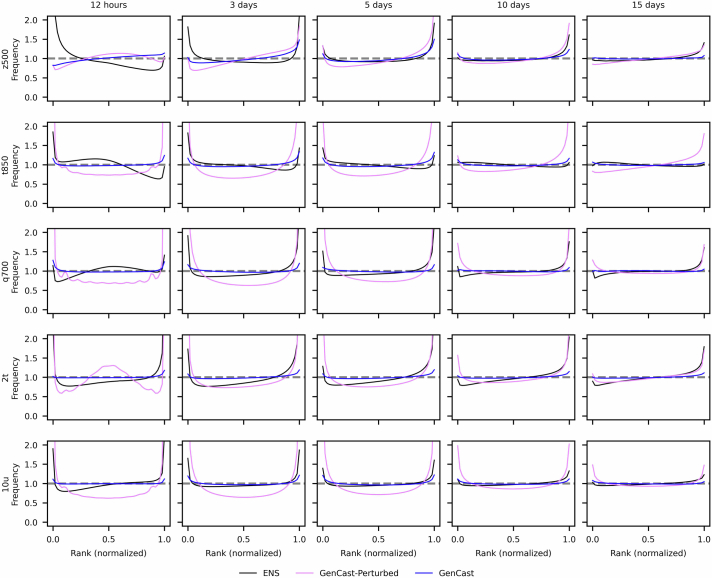
Rank histograms show that GenCast’s ensembles are well calibrated. GenCast generally has very flat rank histograms, in many cases flatter than those of ENS^4^, and in almost all cases substantially flatter than GenCast-Perturbed. These plots show rank histograms of GenCast, ENS, and GenCast-Perturbed at different lead times for z500, t850, q700, 2t and 10 u.

Generally, GenCast exhibits good calibration according to these verification methods, similar to that exhibited by ENS. The spread/skill scores of GenCast are typically fractionally less than but very close to 1 (Fig. 3b–f and Supplementary Fig. B1) and also tend to have flat rank histograms (Extended Data Fig. 2 and Supplementary Fig. B2). By contrast, GenCast-Perturbed is consistently overconfident, showing spread/skill scores substantially less than 1 and U-shaped rank histograms.

### Local surface extremes

**Extended Data Fig. 3:**
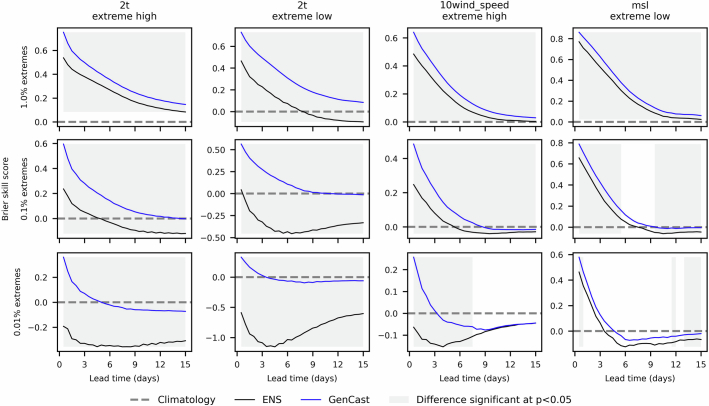
GenCast provides better forecasts of extreme surface weather than ENS (Brier skill scores). The plots show Brier skill scores for ENS^4^ and GenCast for forecasts of extreme high temperatures, extreme low temperatures, extreme high wind speed, and extreme low mean sea level pressure, with each row computing the metric for different percentile threshold. A skill score of 1 represents a perfect score; a skill score of 0 represents the skill of climatology; and below 0 represents a skill worse than climatology. Regions for which GenCast is better than ENS with statistical significance (*P* < 0.05) are shaded in grey. GenCast shows significant improvement over ENS for all thresholds, variables, and lead times shown, with the exception of lead times longer than 7 days for >0.01 percentile for high 10 m wind speed, and certain lead times for <0.01 and <0.1 percentile for low mean sea level pressure where the improvement is not significant.

Extreme heat, cold, wind and other severe surface weather pose serious threats to lives, health and property but can be anticipated and prepared for with the help of quality probabilistic forecasts. We assess the predictions of GenCast of whether 2 m temperature, 10 m wind speed or mean sea level pressure will exceed some extreme percentile of the climatological distribution. When comparing Brier skill scores (Supplementary Information section A.5.5)—a standard metric for evaluating probabilistic forecasts of binary events—GenCast significantly (*P* < 0.05) outperforms ENS on predicting the exceedance of the 99.99th, 99.9th and 99th percentiles for high 2 m temperature and 10 m wind speed, and for extremely low temperature and mean sea level pressure below the 0.01st, 0.1st and 1st percentiles (Extended Data Fig. 3). This is true across all lead times, except for lead times longer than 7 days for >99.99th percentile 10 m wind speed and certain lead times for <0.01 and <0.1 percentile mean sea level pressure, for which the improvement is not significant.

In decision-making about extreme weather events, it is often worth making preparations given even a relatively small probability of the event in question^34,35^. However, skill in this important regime is not well captured by the Brier score, which places equal weight on all probability decision thresholds^36^. We thus use relative economic value (REV) curves^37,38^ (for full details, see Supplementary Information section A.5.6) as a standard tool to characterize the potential value of a forecast over a range of different probability decision thresholds. Each decision threshold corresponds to a cost/loss ratio for a decision problem in which we must trade off the cost of making preparations against the loss incurred if we encounter the weather event unprepared. We draw attention in particular to lower cost/loss ratios, which are common in decision-making around extreme weather. REV is normalized relative to the value of a climatological forecast (REV = 0) and a perfect forecast (REV = 1). Note that despite the name, the ‘value’ in REV need not be economic or monetary, merely quantifiable in relative terms.

**Extended Data Fig. 4:**
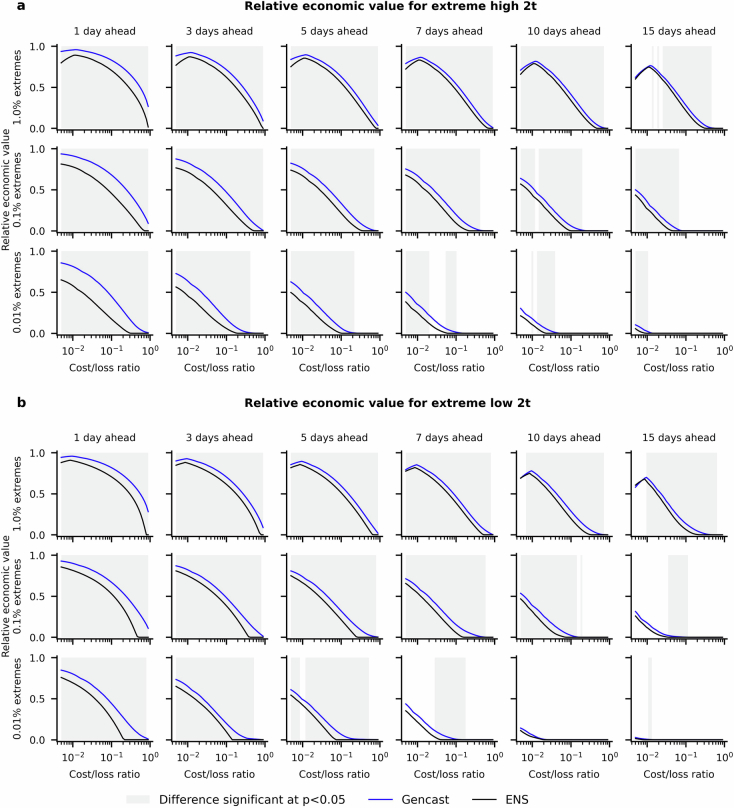
GenCast provides better forecasts of extreme surface weather than ENS (REV panel 1). **a**,**b**, These plots compare relative economic value achieved by GenCast and ENS^4^ forecasts of **a**, extreme high 2 m temperature and **b**, extreme low 2 m temperature, for various extreme thresholds and lead times. Regions for which GenCast is better then ENS with statistical significance are shaded in grey. GenCast is significantly better than ENS (*P* < 0.05) for up to 7 days lead time in many cases, and up to 15 days lead time in some others, while in some cases the differences are not statistically significant.

**Extended Data Fig. 5:**
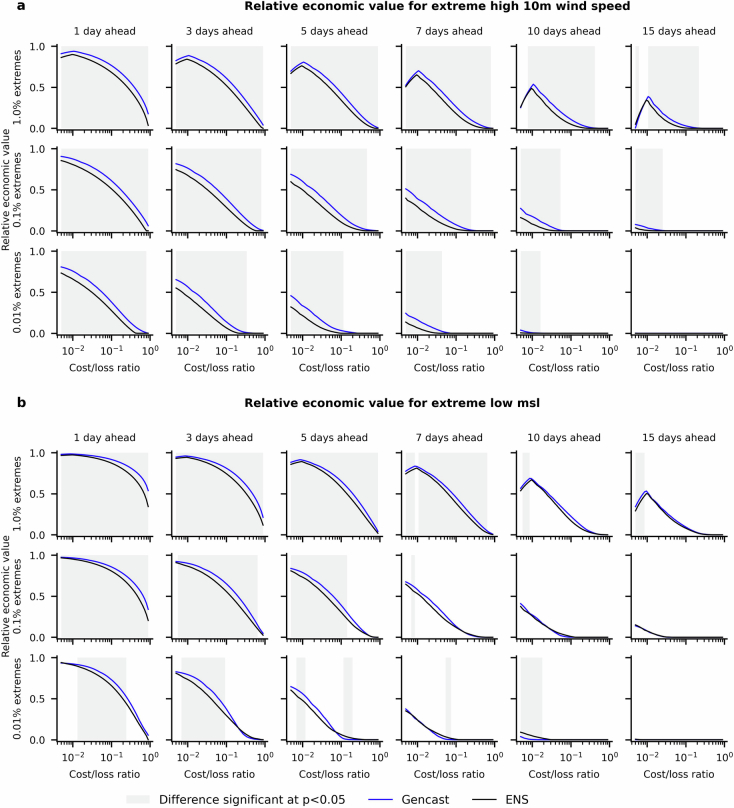
GenCast provides better forecasts of extreme surface weather than ENS (REV panel 2). **a**,**b**, These plots compare relative economic value achieved by GenCast and ENS^4^ forecasts of **a**, extreme high 10 m wind speed and **b**, extreme low mean sea level pressure, for various extreme thresholds and lead times. Regions for which GenCast is better then ENS with statistical significance are shaded in grey. GenCast is significantly better than ENS (*P* < 0.05) for up to 7 days lead time in many cases, and up to 15 days lead time in some others, while in some cases the differences are not statistically significant.

Figure 3g,h shows results for predictions of whether 2 m temperature and 10 m wind speed will exceed the 99.99th percentile relative to climatology. GenCast (blue curves) yields significantly (*P* < 0.05) better REV than ENS (black curves) across all cost/loss ratios, at lead times of 1 day, 5 days and 7 days (solid, dashed and dash-dot lines, respectively), with the only exceptions being those (cost/loss, lead time) combinations at which neither model outperforms climatology. Extended Data Figs. 4 and 5 show that GenCast also provides better forecasts of other levels of extreme events (other exceedance percentiles), and for other variables, including extreme low temperature and low mean sea level pressure.

## Skilful joint forecast distributions

Physical constraints impose spatiotemporal dependency structure on the joint distribution of weather. For example, we know a cyclone will be a spatially local phenomenon following a single trajectory, even though its exact path may be uncertain. These spatiotemporal dependencies influence the distribution of derived quantities that are important for applications, such as cyclone tracks, or the total wind power across a specific set of wind farms. For example, the variance of the total wind power output from a set of wind farms increases when positive correlation between their wind speeds increases. It is thus important that a weather model captures these dependencies in its predictive joint distribution. We perform three evaluations on derived variables that require capturing specific aspects of this joint structure.

### Spatially pooled evaluation

Neighbourhood verification is an established method from the meteorological literature that evaluates spatially pooled versions of forecasts^39^. Pooling mitigates the double penalty problem of standard per-grid-cell evaluation, in which the models are penalized more for predicting a feature (such as a storm) at a spatial offset than not at all. Moreover, the distribution of a spatially pooled weather quantity is influenced by spatial dependencies, and thus probabilistic pooled metrics evaluate how well a model captures some of the spatial dependency structure inherent in weather states.

We compute average-pooled and max-pooled versions of the marginal CRPS scorecard. Forecasts and analysis targets are aggregated over circular spatial regions distributed to jointly cover the surface of Earth, and CRPS is computed on these pooled quantities for a range of pooling region sizes from 120 km to 3,828 km.

**Extended Data Fig. 6:**
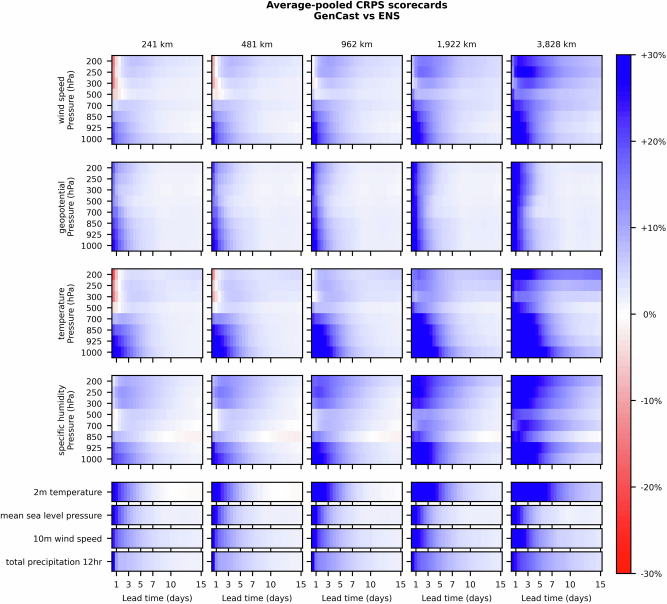
GenCast ensembles capture spatial dependency structure (average-pooled CRPS). Average-pooled CRPS scorecard comparing GenCast and ENS at varying spatial scales, dark blue (resp. red) means GenCast is 30% better (resp. worse) than ENS, and white means they perform equally. We aggregate the u-component and v-component of wind into wind speed, and include 12 hr accumulated precipitation (tp12h) in our surface variables. This results in 5,400 pooled verification targets across all variables, lead times, and spatial scales. Aggregating over all pooled verification targets, GenCast outperforms ENS’s average-pooled CRPS on 98.1% of targets.

**Extended Data Fig. 7:**
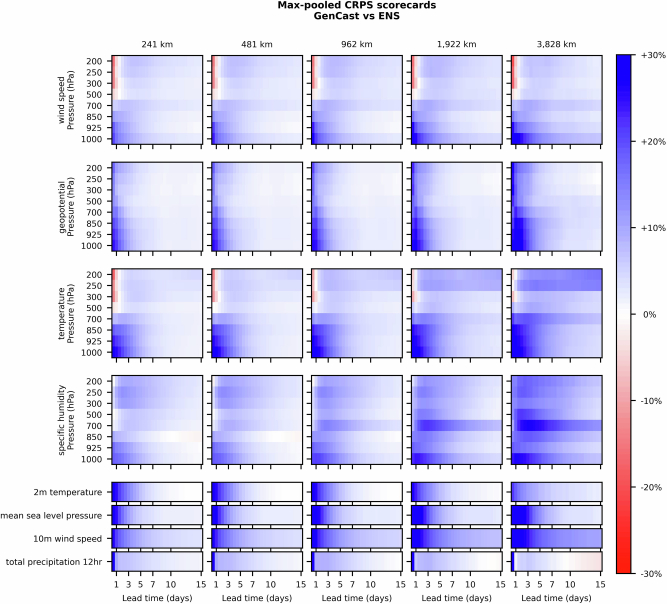
GenCast ensembles capture spatial dependency structure (max-pooled CRPS). Max-pooled CRPS scorecard comparing GenCast and ENS^4^ at varying spatial scales, dark blue (resp. red) means GenCast is 30% better (resp. worse) than ENS, and white means they perform equally. We aggregate the u-component and v-component of wind into wind speed, and include 12 hr accumulated precipitation (tp12h) in our surface variables. This results in 5,400 pooled verification targets across all variables, lead times, and spatial scales. Aggregating over all pooled verification targets, GenCast outperforms ENS’s average-pooled CRPS on 97.6% of targets.

**Extended Data Fig. 8:**
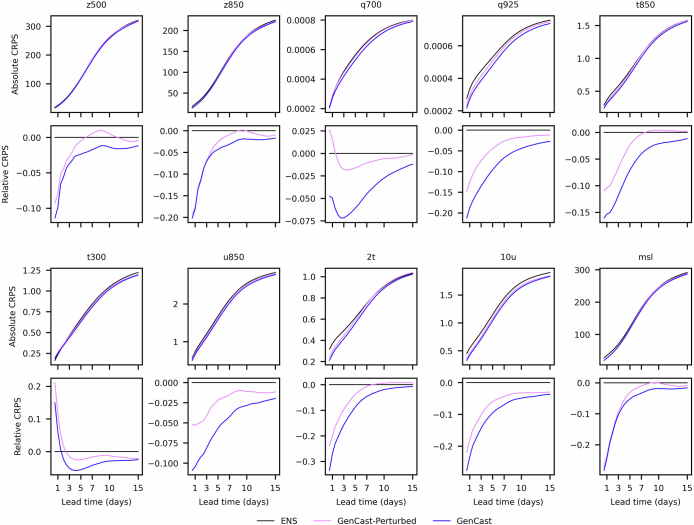
GenCast is more skillful than both ENS and GenCast-Perturbed. Line plots are shown for a set of representative variables, showing absolute and relative CRPS on z500, z850, q700, q925, t850, t300, u850, 2t, 10 u and msl. GenCast achieves the best CRPS, and GenCast-Perturbed is competitive with ENS^4^.

Across all 5,400 pooled verification targets—spanning each variable, level, lead time and spatial scale—GenCast outperforms ENS on average-pooled CRPS in 98.1% of targets and on max-pooled CRPS in 97.6% of targets, with relative performance increasing at larger scales (Extended Data Figs. 6 and 7). GenCast-Perturbed is competitive with or better than ENS on 86% of targets for average-pooled CRPS, but only 50% of targets for max-pooled CRPS (Supplementary Figs. B15 and B16), and in both cases is worse than GenCast on 94% and 97% of targets, respectively. This suggests that GenCast captures spatial dependencies better than ENS and GenCast-Perturbed across all surface and atmospheric variables.

### Regional wind power forecasting

In the electricity sector, power grid operators use regional wind power forecasts for tasks such as unit commitment and reserve quantification^40^, in which leveraging forecast uncertainty can improve decision-making^41,42^. However, forecast errors make it harder to ensure the balance of supply and demand, increasing reliance on fossil fuel-based spinning reserves^40^, thus undermining the potential of wind power for reducing carbon emissions^43^.

To estimate the potential impact of GenCast in wind energy applications, we conducted a simplified regional wind power forecasting experiment, in which 10 m wind speed of forecasts and analysis targets are interpolated at all 5,344 wind farm locations from the Global Power Plant Database^44^. These 10 m wind speeds are then converted to wind power using a standard idealized power curve (Supplementary Fig. A1) multiplied by the nominal capacity of each wind farm. Wind power (in megawatts) is then summed across arbitrary groupings of wind farms defined by the pooling regions from the above spatially pooled evaluation with sizes of 120 km, 240 km and 480 km.

**Fig. 4:**
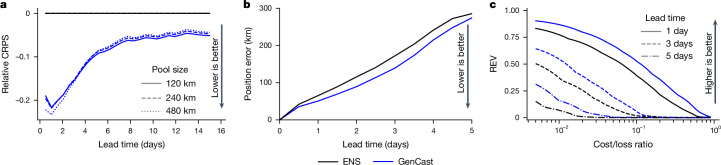
GenCast outperforms ENS on regional wind power and tropical cyclone track forecasting. **a**, Relative CRPS of the total wind power summed across wind farm locations^44^ in pooling regions of different sizes. **b**, Position error of ensemble mean cyclone tracks. **c**, REV of tropical cyclone track probability forecasts at lead times of 1 day, 3 days and 5 days. All plots show a comparison of GenCast and ENS^4^.

GenCast outperforms the CRPS of ENS by around 20% up to lead times of 2 days, 10–20% from 2 days to 4 days, and retains statistically significant (*P* < 0.05) improvements out to 7 days (Fig. 4a and Supplementary Fig. B17). This is a substantially greater improvement than that provided by GenCast-Perturbed (Supplementary Fig. B18).

It is important to note that this experiment does not account for complications of curtailment because of non-weather effects (for example, turbine maintenance) or grid topology. We also use 10 m wind speeds; most turbines are closer to 100 m above the ground. Nonetheless, these results indicate that GenCast provides more skilful wind forecasts that can capture joint spatial structure across real-world wind farm sites, indicating a potential value for the management and use of wind energy.

### Tropical cyclones

Tropical cyclones cause thousands of deaths and tens of billions of dollars in damages on average every year. Mitigating these devastating consequences depends on accurate predictions of cyclone trajectories^45^. Preventative measures may be justified even when the risk of a cyclone impact is low, making probabilistic cyclone forecasts particularly important^34,46^. Moreover, cyclones are defined by the interactions of multiple weather variables across different atmospheric levels and over time, as such probabilistic cyclone trajectory forecasting constitutes a substantial test of both the tails and the spatiotemporal joint structure in the predictive distribution of a model. To assess the cyclone prediction skill of GenCast and ENS, we apply the TempestExtremes tropical cyclone tracker^47^ to GenCast, ENS, ERA5 and HRES-fc0 and evaluate the two models using established deterministic and probabilistic verification methods from the tropical cyclone literature.

First, we evaluate the position error of ensemble mean cyclone trajectories from GenCast and ENS, using a pairing procedure to ensure evaluation on the same set of cyclones. The ensemble mean track of GenCast is consistently more skilful than that of ENS. On average, GenCast gives a 12-h advantage in accuracy between 1 day and 4 days ahead (Fig. 4b), with significantly (*P* < 0.05) lower error between 12 h and 3.5 day lead times (inclusive, Supplementary Fig. B9).

Ensemble mean cyclone trajectories provide intuitive summaries of ensemble forecasts, but do not capture their uncertainty (or even possible multi-modality), and cannot be used to assess the ability of a model to predict cyclogenesis. We, therefore, also evaluate forecast track probability fields from GenCast and ENS—computed as the fraction of ensemble members that predict a cyclone centre passing through a given 1° grid box at a given time^48^. Cyclones are typically associated with low cost/loss ratios given their potentially severe consequences. The track probability forecasts of GenCast outperform those of ENS, achieving better REV at all cost/loss ratios, with the only exceptions being large cost/loss ratios for which neither model outperforms climatology (Fig. 4c). These improvements are significant (*P* < 0.05) in almost all cases out to 7 day lead times (Supplementary Fig. B11). This shows that GenCast can provide substantial value in decisions about when and how to prepare for tropical cyclones^34^. See Methods for the evaluation and cyclone tracker details and Supplementary Information section C.1 for additional cyclone visualizations.

## Conclusion

Our results indicate that probabilistic weather forecasts based on MLWP can be more skilful and faster to generate than the top NWP-based ensemble forecast, ENS of ECMWF. GenCast succeeds across three key desiderata for probabilistic weather models. First, GenCast generates ensembles of sharp individual weather trajectories with realistic power spectra, rather than sets of summary statistics such as conditional means. Second, the marginal forecast distributions of GenCast (that is, forecasts for a given place and time) are well-calibrated and provide more skilful predictions than those of ENS, including better predictions of extreme events. Third, GenCast outperforms ENS across several evaluations that require capturing spatial and temporal dependencies in the joint distribution: pooled evaluation, regional wind power forecasting and tropical cyclone track prediction.

Going forward, GenCast could be further improved for operational settings in several ways. GenCast operates at 0.25° resolution, the current maximum resolution of global reanalysis data. However, it may be useful to scale up to higher resolution to support additional applications and match the upgraded resolution of ENS (as of mid-2023) of 0.1°. As a diffusion model, GenCast is computationally more expensive than an equivalent deterministic MLWP architecture, because it requires multiple function evaluations to sample each forecast time step. To efficiently scale to higher resolution or to move towards computational parity with GenCast-Perturbed and similar models, distillation^49^ and other efficiency techniques should be explored. Furthermore, previous work has shown that the performance of MLWP models that are trained on reanalysis can be further improved by fine-tuning using operational data, such as HRES analysis inputs and targets^30^. This underscores the importance for GenCast of traditional NWP-based data assimilation for providing training and initialization data.

Together, our results open a new front in weather forecasting, promising greater accuracy, efficiency and accessibility across a wide range of settings. More generally, our work demonstrates that cutting-edge generative AI methods can capture very high-dimensional and complex distributions over rich temporal dynamics, with sufficient accuracy and reliability to support effective decision-making in crucial applications.

## Methods

### Task definition and general approach

A general formulation of the task of probabilistic weather forecasting from the present time *t* = 0 into the future is to model the joint probability distribution $P({\bar{{\bf{X}}}}^{0:T}| {{\bf{O}}}^{\le 0})$, where *T* is the forecast horizon, ${\bar{{\bf{X}}}}^{t}$ denotes the atmospheric state at time *t* and **O**^≤0^ are observations made up to the forecast initialization time *t* = 0. This joint distribution can be factored as

$$P({\bar{{\bf{X}}}}^{0:T}| {{\bf{O}}}^{\le 0})=\mathop{\underbrace{P({\bar{{\bf{X}}}}^{0}| {{\bf{O}}}^{\le 0})}}\limits_{{\rm{State}}\,{\rm{inference}}}\,\mathop{\underbrace{P({\bar{{\bf{X}}}}^{1:T}| {\bar{{\bf{X}}}}^{0})}}\limits_{{\rm{Forecast}}\,{\rm{model}}}$$

Our innovation in this work is an MLWP-based Forecast model, and we adopt a traditional NWP-based State inference approach. We make several approximations to the above general formulation, as follows.

Full atmospheric states are not directly observed, and so we approximate each ${\bar{{\bf{X}}}}^{t}$ with a best-estimate NWP-based analysis state **X**^*t*^, which has been generated at finite resolution, using a window of observations in a process known as data assimilation. In our case, each **X**^*t*^ is an 84 × 720 × 1,440 array, which includes six surface variables and six atmospheric variables at each of 13 vertical pressure levels (Extended Data Table 1), on a 0.25° latitude–longitude grid. We generate 15-day forecasts, at 12 h steps, so *T* = 30.

As a first, standard approximation^29^, we use analysis **X**^0:*T*^ as evaluation targets. This means we are in effect evaluating the forecast of each model as a predictive distribution,

$$P({{\bf{X}}}^{0:T}| {{\bf{O}}}^{\le 0}),$$

over sequences of future best-estimate NWP analyses.

Second, we wish to rely on a Markov assumption, but although the underlying atmospheric state sequence ${\bar{{\bf{X}}}}^{1:T}$ is Markov, it is only partially observed in **X**^1:*T*^. In our models GenCast and GenCast-Perturbed, we make a weaker second-order Markov approximation, under which we factorize

$$P({{\bf{X}}}^{-1},{{\bf{X}}}^{0:T}| {{\bf{O}}}^{\le 0})=P({{\bf{X}}}^{0},{{\bf{X}}}^{-1}| {{\bf{O}}}^{\le 0})\mathop{\prod }\limits_{t=1}^{T}P({{\bf{X}}}^{t}| {{\bf{X}}}^{t-1},{{\bf{X}}}^{t-2}).$$

We found that conditioning on two previous time steps works better than one.

For GenCast, the initialization *P*(**X**^0^, **X**^−1^|**O**^≤0^) is handled by fixing (**X**^0^, **X**^−1^) to their values obtained from two consecutive best-estimate analyses from the ERA5 dataset^27^. For GenCast-Perturbed, additional perturbations are added, see Supplementary Information section A.4. With initialization dealt with, the problem is reduced to modelling *P*(**X**^*t*^|**X**^*t*−1^, **X**^*t*−2^), and samples of **X**^1:*T*^ can be generated autoregressively.

### Diffusion model specification

Beyond image and video generation, diffusion models^21–23^ have also been applied in the geophysical domain, to tasks including data assimilation^50^, NWP ensemble emulation^51^ and climate downscaling^52^. In this work, we model *P*(**X**^*t*^|**X**^*t*−1^, **X**^*t*−2^) with a diffusion model, which enables us to sample forecast trajectories.

Rather than sampling **X**^*t*^ directly, our approach is to sample a residual **Z**^*t*^ with respect to the most recent weather state **X**^*t*−1^, in which the residuals have been normalized to unit variance on a per-variable and per-level basis as was done for GraphCast^2^. **X**^*t*^ is then computed as **X**^*t*^ = **X**^*t*−1^ + *S***Z**^*t*^, where *S* is a diagonal matrix that inverts the normalization. The one exception to this is precipitation, for which we set **X**^*t*^ = *S***Z**^*t*^ without adding the previous state.

We broadly follow the diffusion framework presented in ref. ^21^, and refer the reader to their paper for a more rigorous introduction to diffusion, as well as a detailed treatment of the available modelling decisions. We adopt their choices of noise schedule, noise scaling, loss weighting by noise level and preconditioning. However, we make changes to the noise distribution, the training-time distribution of noise levels and add additional loss weightings, all of which are described below. These changes improve performance on the task of probabilistic weather forecasting.

#### Sampling process

The sampling process begins by drawing an initial sample ${{\bf{Z}}}_{0}^{t}$ from a noise distribution on the sphere *P*_noise_(·|*σ*_0_), at a high initial noise level *σ*_0_. After *N* steps of transformation, we end up at ${{\bf{Z}}}_{N}^{t}:= {{\bf{Z}}}^{t}$, our sample from the target distribution at noise level *σ*_*N*_ = 0. To take us from one to the other, we apply an ODE solver to a probability flow ODE^21,22^. Each step of this solver is denoted by *r*_*θ*_ (Fig. 1), with

$${{\bf{Z}}}_{i+1}^{t}={r}_{\theta }({{\bf{Z}}}_{i}^{t};{{\bf{X}}}^{t-1},{{\bf{X}}}^{t-2},{\sigma }_{i+1},{\sigma }_{i})$$

taking us from noise level *σ*_*i*_ to the next (lower) noise level *σ*_*i*__+1_, conditioned on (**X**^*t*−1^, **X**^*t*−2^).

We use the second-order DPMSolver++2S solver^53^, augmented with the stochastic churn (again making use of *P*_noise_) and noise inflation techniques used in ref. ^21^ to inject further stochasticity into the sampling process. In conditioning on previous time steps, we follow the conditional denoising estimator approach outlined and motivated in ref. ^54^.

Each step *r*_*θ*_ of the solver makes use of a learned denoiser *D*_*θ*_ with parameters *θ*, described in detail below. We take *N* = 20 solver steps per generated forecast time step. As we are using a second-order solver, each step *r*_*θ*_ requires two function evaluations of the denoiser *D*_*θ*_ (except the last step which requires only a single evaluation). This results in 39 function evaluations in total. See Supplementary Information section A.2.1 for further details, including a full list of sampling hyperparameters.

#### Noise distribution on the sphere

At the core of a diffusion model is the addition and removal of noise, drawn from some distribution *P*_noise_(·|*σ*) parameterized by noise level *σ*. When using diffusion to generate natural images^55^, *P*_noise_ is usually chosen to be independent and identically distributed (i.i.d.) Gaussian. However, we have found it beneficial to use a noise distribution that better respects the spherical geometry of global weather variables. Rather than sampling i.i.d. Gaussian noise on the latitude–longitude grid, we instead sample isotropic Gaussian white noise on the sphere, which is then projected onto the grid. This choice of *P*_noise_ has the consequence that the noise has a flat spherical harmonic power spectrum in expectation. For motivation and details of these changes, see Supplementary Information section A.2.3.

#### Denoiser architecture

To recap, our diffusion sampling process involves taking several solver steps *r*_*θ*_, and each solver step calls a denoiser *D*_*θ*_ as part of its computation. We parameterize the denoiser *D*_*θ*_ following ref. ^21^ as a preconditioned version of a neural network function *f*_*θ*_.

$${D}_{\theta }({{\bf{Z}}}_{\sigma }^{t};{{\bf{X}}}^{t-1},{{\bf{X}}}^{t-2},\sigma )\,:= \,{c}_{{\rm{skip}}}(\sigma )\cdot {{\bf{Z}}}_{\sigma }^{t}+{c}_{{\rm{out}}}(\sigma )\cdot {f}_{\theta }({c}_{{\rm{in}}}(\sigma ){{\bf{Z}}}_{\sigma }^{t};\,{{\bf{X}}}^{t-1},{{\bf{X}}}^{t-2},{c}_{{\rm{noise}}}(\sigma )).$$

Here ${{\bf{Z}}}_{\sigma }^{t}$ denotes a noise-corrupted version of the target **Z**^*t*^ at noise level *σ*, and *c*_in_, *c*_out_, *c*_skip_ and *c*_noise_ are preconditioning functions taken from table 1 in ref. ^21^, with *σ*_data_ = 1 because of the normalization of the targets.

The architecture used for *f*_*θ*_ is related to the GraphCast architecture^2^. To be precise, the Encoder and Decoder architectures stay the same, and those inputs to the encoder corresponding to the previous two time steps are normalized to zero mean and unit variance in the same way. However, unlike in GraphCast, which uses a similar message-passing GNN for the Processor architecture as in the Encoder and Decoder, in GenCast the Processor is a graph-transformer model operating on a spherical mesh that computes neighbourhood-based self-attention. Unlike the multimesh used in GraphCast, the mesh in GenCast is a six-times refined icosahedral mesh^2^, with 41,162 nodes and 246,960 edges. The Processor consists of 16 consecutive standard transformer blocks^26,56^, with feature dimension equal to 512. The four-head self-attention mechanism in each block is such that each node in the mesh attends to itself and to all other nodes that are within its 32-hop neighbourhood on the mesh.

To condition on previous time steps (**X**^*t*−1^, **X**^*t*−2^), we concatenate these along the channel dimension with the input to be denoised and feed this as input to the model. Conditioning on noise level *σ* is achieved by replacing all layer-norm layers in the architecture with conditional layer-norm^57^ layers. We transform log noise levels into a vector of sine–cosine Fourier features at 32 frequencies with base period 16 and pass them through a two-layer MLP to obtain 16-dimensional noise-level encodings. Each of the conditional layer-norm layers applies a further linear layer to output replacements for the standard scale and offset parameters of layer norm, conditioned on these noise-level encodings.

#### Training the denoiser

At training time, we apply the denoiser to a version of the target **Z**^*t*^, which has been corrupted by adding noise **ε** ~ *P*_noise_(·|*σ*) at noise level *σ*:

$${{\bf{Y}}}^{t}={D}_{\theta }({{\bf{Z}}}^{t}+{\boldsymbol{\varepsilon }}\,;{{\bf{X}}}^{t-1},{{\bf{X}}}^{t-2},\sigma ).$$

We train its output, denoted as **Y**^*t*^, to predict the expectation of the noise-free target **Z**^*t*^ by minimizing the following mean-squared-error objective weighted per elevation level and by latitude–longitude cell area,

$$\sum _{t\in {D}_{{\rm{train}}}}E\left[\lambda (\sigma )\frac{1}{| G| | \,J| }\sum _{i\in G}\sum _{j\in J}{w}_{j}{a}_{i}{({Y}_{i,j}^{t}-{Z}_{i,j}^{t})}^{2}\right],$$

where

- *t* indexes the different time steps in the training set *D*_train_;
- *j* ∈ *J* indexes the variable, and for atmospheric variables the pressure level, that is, *J* = {z1000, z850, …, 2t, msl};
- *i* ∈ *G* indexes the location (latitude and longitude coordinates) in the grid;
- *w*_*j*_ is the per-variable-level loss weight, set as in GraphCast^2^ with the additional sea surface temperature variable weighted at 0.1;
- *a*_*i*_ is the area of the latitude–longitude grid cell, which varies with latitude and is normalized to unit mean over the grid;
- *λ*(*σ*) is the per-noise-level loss weight in ref. ^21^; and
- the expectation is taken over *σ* ~ *P*_train_, **ε** ~ *P*_noise_(·; *σ*).

Instead of using the log-normal distribution for *P*_train_ that is suggested in ref. ^21^, we construct a distribution whose quantiles match the noise-level schedule used for sample generation, assigning a higher probability to noise levels that are closer together during sampling. Details are in Supplementary Information section A.2.2. As done by GraphCast^2^, we weight the squared error made at each latitude–longitude grid cell by a per-variable-level loss weight, as well as the normalized area of that grid cell; this is also a departure from ref. ^21^.

Unlike GraphCast, which is fine-tuned by back-propagating gradients through 12-step trajectories (3 days with 6 h steps) produced by feeding the model its own predictions as inputs during training, GenCast is only ever trained using targets that consist of the next 12-h state, without ever being provided its own predictions on previous steps as inputs.

#### Resolution training schedule

The GenCast results reported in this paper were generated by a model that was trained in a two-stage process. Stage 1 was a pre-training stage, taking 2 million training steps. During this stage, the ground truth dataset was bilinearly downsampled from 0.25° to 1° and the denoiser architecture used a 5-refined icosahedral mesh. This training stage takes a little over 3.5 days using 32 TPUv5 instances. After this training phase was complete, stage 2 was conducted, fine-tuning the model to 0.25°, taking 64,000 further training steps. This takes just under 1.5 days using 32 TPUv5 instances. During stage 2, the ground truth data is kept at 0.25°, and the denoiser architecture is updated to take in 0.25° data and output 0.25° outputs and to operate on a 6-refined icosahedral mesh. The GNN and graph-transformer architectures are such that the same model weights can operate on the higher data and mesh resolutions without any alterations. We do, however, make a minor modification before beginning the fine-tuning stage to decrease the shock to the model of operating on higher resolution data. In the Encoder GNN, which performs message passing between the grid and mesh nodes, when the data resolution increases from 1° to 0.25°, the number of messages being received by each mesh node increases by a factor of 16. To approximately preserve the scale of the incoming signal to all mesh nodes at the start of fine-tuning, we divide the sum of these message vectors by 16. The optimization hyperparameters used for both stages of training are detailed in Supplementary Information section A.3.

### Training data

We trained GenCast on a dataset built from the ERA5 archive of ECMWF^27^, a large corpus of global reanalysis data. Our dataset contains the best-estimate analyses of ERA5 for a subset of the available variables, on 13 pressure levels (see Extended Data Table 1 for a complete list of variables and pressure levels), on a 0.25° equiangular grid. We also subsampled the temporal resolution from 1 h to 6 h, corresponding to 00:00, 06:00, 12:00 and 18:00 UTC times each day. From this dataset, we extracted sequences at 12-h temporal resolution (sequences of 00/12 UTC or 06/18 UTC times) to train GenCast.

Although its temporal resolution is hourly, ERA5 only assimilates observations in 12-h windows, from 21 UTC–09 UTC and 09 UTC–21 UTC. This means that steps taken within a single 12-h assimilation window have a different, less dispersed distribution to those that jump from one window into the next. By choosing a 12-h time step, we avoid training on this bimodal distribution and ensure that our model always predicts a target from the next assimilation window.

For accumulated variables such as precipitation, instead of subsampling the data in time, we accumulated values over the 12-h period preceding each time.

Our dataset covers the period 1979–2019. During the development phase of GenCast, we used dates from 1979 to 2017 for training and validated results in 2018. Before starting the test phase, we froze all model and training choices, retrained the model on data from 1979 to 2018 and evaluated results in 2019.

### GenCast-Perturbed training protocol

GenCast-Perturbed is trained by taking the GenCast architecture for *f*_*θ*_ described above in the section ‘Denoiser architecture’, removing the conditioning on noise level and noisy targets, and training it at 0.25° resolution as a deterministic forecast model using the same training dataset. It takes (**X**^*t*−1^, **X**^*t*−2^) as inputs and outputs a single forecast of the normalized residual target **Z**^*t*^. It is trained to minimize the mean-squared error of its single-step 12-h forecasts. Specifically, we minimize

$$\sum _{t\in {D}_{{\rm{train}}}}{\rm{E}}\left[\frac{1}{| G| | \,J| }\sum _{i\in G}\sum _{j\in J}{w}_{j}{a}_{i}{({Y}_{i,j}^{t}-{Z}_{i,j}^{t})}^{2}\right],$$

where in this case *Y*^*t*^ is the deterministic forecast rather than the output of a denoising step and $t,j\in J,i\in G,{w}_{j},{a}_{i}$ are all defined as above. The optimization hyperparameters are detailed in Supplementary Information section A.3.

### Statistical methods

We compare GenCast with ENS on several verification metrics (detailed in Supplementary Information section A.5) computed on our 2019 evaluation set. For each relevant metric (and where applicable at each lead time, level, quantile and cost/loss ratio), we test the null hypothesis of no difference in the metric between GenCast and ENS, against the two-sided alternative. Specifically, we are testing for differences in the values of the metrics that would be attained in the limit of infinite years of evaluation data, assuming the stationarity of the climate.

Most of our metrics are computed from time series of spatially aggregated values given at *n* = 730 12-hourly initialization times from 2019. For these metrics, we apply a paired-differences significance test based on the stationary block bootstrap^58^, which handles temporal dependence by resampling blocks of the time-series data from which the metric is computed. We use automatic block length selection^59,60^.

By contrast, deterministic cyclone position error is only obtained for a given cyclone at select times at which pairing criteria are met. For this metric, we instead perform a cluster bootstrap^61^ that assumes independence between (but not within) cyclones.

We base all our tests on bias-corrected and accelerated (bca) bootstrap confidence intervals^62^. Further details of the statistical tests are given in Supplementary Information section A.6.

### Local surface extremes evaluation

We evaluate GenCast and ENS on the task of predicting when surface weather variables exceed high (99.99th, 99.9th and 99th) and low (0.01st, 0.1st and 1st) climatological percentiles. These percentiles are computed per latitude–longitude using 7 recent years of 6-hourly data from 2016 to 2022, taken from the corresponding ground truth dataset for each model (ERA5 for GenCast and HRES-fc0 for ENS). For each latitude–longitude, the 99.99th and 0.01st percentiles correspond to a return period of approximately 7 years.

### Tropical cyclone evaluation

We extract cyclone trajectories from ENS and GenCast forecasts using the same cyclone tracker, TempestExtremes, downsampling ENS forecasts from a 6-h to 12-h resolution for a fair comparison with GenCast. We also apply the same cyclone tracker to chunks of HRES-fc0 and ERA5 spanning the same time period as the forecast trajectories, generating ground truth cyclone tracks for each model and initialization. The ensemble cyclone forecast skill of each model is then evaluated against its own ground truth. We use established deterministic and probabilistic verification methods from the tropical cyclone literature, detailed below. See Supplementary Information section A.7 for a comparison between our two cyclone evaluations, the motivation behind our choice of ground truth and further cyclone tracker details.

#### Cyclone position error evaluation

We evaluate ensemble mean cyclone trajectory forecasts from GenCast and ENS using position error^48^. To be able to compare GenCast and ENS against the same cyclones (despite being evaluated against different ground truths), we first associate the ERA5 and HRES-fc0 cyclone trajectories with named cyclones from the International Best Track Archive for Climate Stewardship (IBTrACS)^63,64^. Ground truth cyclones that are within 200 km (in geodesic distance) of an IBTrACS cyclone at lead time zero are retained and any others are removed (TempestExtremes and IBTrACS have different definitions of a cyclone, meaning that they do not necessarily identify exactly the same set of cyclones).

Next, for both models, each ensemble member cyclone trajectory is paired to a TempestExtremes named ground truth cyclone if it is within 100 km of that cyclone at lead time zero (otherwise it is removed). We then compute the ensemble mean cyclone location for each named cyclone as the cyclone progresses until fewer than 50% of the ensemble member cyclones remain active and compute the position error between each ensemble mean cyclone centre and its corresponding ground truth cyclone centre. To account for the 6-h offsets between GenCast and ENS initializations, we estimate the position error of ENS at the same 06/18 UTC initializations as GenCast by averaging the two position errors on either side of that initialization with the same lead time^2^. For a fair comparison, we evaluate GenCast and ENS against exactly the same cyclones and lead times by computing average position error over the intersection of named cyclone and lead time pairs for which both a GenCast and ENS ensemble mean track position error exists (Fig. 4b).

#### Cyclone track probability evaluation

To evaluate the probabilistic skill of ensemble cyclone tracks, we compute 1° resolution track probability heatmaps for each time step, in which the predicted probability in each 1° cell is the fraction of ensemble members predicting a cyclone centre within that cell. We choose 1° as it corresponds to 111 km at the equator, which is close to 120 km, a common radius used for defining cyclone track probability^48^. We convert the ground truth cyclone tracks from ERA5 and HRES-fc0 to binary ground truth maps for each initialization time and lead time. Finally, we follow ref. ^34^ in computing the REV of the track probability forecast of each model against their respective binary ground truth heatmaps.

Unlike the paired position error analysis above, this track probability analysis does not restrict the ground truth TempestExtremes tracks to IBTrACS-named cyclones, nor does it evaluate GenCast and ENS against exactly the same cyclones. Owing to differences between HRES-fc0 and ERA5, the TempestExtremes cyclone tracker identifies 23% more cyclones in HRES-fc0 than in ERA5. However, REV accounts for this difference in base rates by virtue of its normalizations with respect to climatology and the perfect forecast (Supplementary Information section A.5.6), and is thus a fair metric to use when comparing methods evaluated against different ground truths. Furthermore, even when using HRES-fc0 as the ground truth of GenCast, which puts GenCast at a disadvantage, GenCast outperforms ENS beyond one day lead times (Supplementary Information Fig. B12).

#### Cyclone tracker

To extract cyclone trajectories from gridded forecasts and analysis datasets, we use the TempestExtremes v2.1 cyclone tracking algorithm^47^. TempestExtremes is open-source on GitHub (https://github.com/ClimateGlobalChange/tempestextremes) and has been used in a wide range of cyclone studies^47^. The algorithm has two stages. The first stage, DetectNodes, finds candidate tropical cyclones where minima in mean sea level pressure are co-located with upper-level warm cores. The second stage, StitchNodes, stitches these locations together to form trajectories. Further details of how the tracker identifies cyclones and what is involved in each tracker stage are given in Supplementary Information section A.7.3, and readers are referred to refs. ^47,65^ for full details.

In their 2017 work, the authors of ref. ^66^ optimized the hyperparameters of TempestExtremes so that when applied to 6-hourly reanalysis datasets the resulting tracks closely match the observed tracks from the IBTrACS dataset^63,64^. We made two changes to the StitchNodes hyperparameters of the tracker (Supplementary Information section A.7.3) to account for the 12-hourly (instead of 6-hourly) temporal resolution of our evaluation, but otherwise left all tracker hyperparameters at their default values. We then used the same set of tracker hyperparameters for each model and each analysis dataset.

As TempestExtremes performs a global optimization when stitching nodes, the track results at a particular lead time depend on raw predictions at nearby lead times. We prepend 10 days of the respective ground truth of the model (ERA5 or HRES-fc0) to each forecast before running the cyclone tracker. This avoids cyclones being dropped when forecasts are initialized close to the end of the lifetime of a cyclone because of the short duration of the cyclone within the forecast period not passing the criteria of the tracker. Similarly, we report only results up to lead times of 9 days despite providing 15 days of predictions to the tracker, because the tracker may drop cyclones that begin close to the end of the forecast period.

### Spatially pooled CRPS evaluation

To evaluate skill at forecasting spatial structure, we compute spatially pooled versions of CRPS. Our approach is an instance of neighbourhood verification^39^, adapted to the surface of a sphere. We define pool centres as the nodes of a *k*-times refined icosahedral mesh. Pooling regions are defined within a fixed geodesic distance of each pool centre, with radii set to the mean distance between mesh nodes. To capture performance at different spatial scales, we do this separately for 6 mesh refinement levels (*k* = 7, 6, …, 2), resulting in a wide range of pool sizes: 120 km, 241 km, 481 km, 962 km, 1,922 km and 3,828 km. We evaluate performance on two types of pooling aggregation: average pooling and max pooling. Forecasts and targets are first aggregated over pooling regions and then standard skill scores are computed on these pooled counterparts. For average pooling, the grid cells are weighted by their area. Finally, to account for slight non-uniformities in the distribution of pooling centres when computing the global average-pooled CRPS, we weight each pooling region by the area of the Voronoi cell of the pooling centre.

These metrics are computed for 2 m temperature, 10 m wind speed, 12-h accumulated precipitation and mean sea level pressure at 0.25° (Supplementary Figs. B13 and B14).

We also compute pooled CRPS scorecards for wind speed, geopotential, temperature and specific humidity at all pressure levels (Extended Data Figs. 6 and 7 and Supplementary Figs. B15 and B16). To reduce the computational cost of these pooled scorecard evaluations that include all pressure levels, forecasts and targets were subsampled to 1° before pooling. In this case, we skipped the smallest pool size because 120 km corresponds to approximately 1° at the equator, making it similar to a univariate evaluation of the subsampled forecasts.

Supplementary Information section A.8 provides further motivation and details on the pooled metrics evaluation.

### Regional wind power evaluation

For the regional wind power forecasting experiment, we use all 5,344 wind farm locations and their nominal capacities from the Global Power Plant Database (GPPD)^44^, which captures about 40% of all global wind farm capacity as of 2020 (ref. ^44^). We first bilinearly interpolate 10 m wind speed forecasts and analysis states at each wind farm location. We then map 10 m wind speed to load factor—the ratio between the actual wind turbine power output and the maximum power output—using an idealized International Electrotechnical Commission Class II 2 MW turbine power curve from the WIND Toolkit^67^. This power curve has a cut-in speed of 3 ms^−^^1^, maximum output at 14 ms^−^^1^ and curtailment at 25 ms^−^^1^ (Supplementary Fig. A1). The load factor is then multiplied by the nominal capacity to obtain idealized power generation in megawatts at each wind farm.

To generate arbitrary groupings of wind farms across the globe at a range of spatial scales, we use a similar procedure to the pooled evaluation. Pooling centres are defined on a 7-times refined icosahedral mesh and separate evaluations performed using pool sizes of 120 km, 240 km and 480 km. The 120 km scale contains 3,648 groups with a mean capacity of 272 MW, the 240 km scale contains 7,759 groups with a mean capacity of 513 MW and the 480 km scale contains 15,913 groups with a mean capacity of 996 MW. The power output is summed over wind farm sites in each group and CRPS is computed for this derived quantity. We then compute the average CRPS across all wind farm groups. By using power as the target variable, more weight is applied to pools containing more wind farm capacity in the global average CRPS.

### Accounting for assimilation windows

During our 2019 test period, ENS was initialized with analyses whose assimilation window had between 3 h and 5 h of look-ahead beyond the stated initialization time^68^. The 06/18 UTC ERA5 initializations of the ML models afford them only 3 h of look-ahead. The 00/12 UTC states of ERA5 have 9 h of look-ahead, which we show in Supplementary Fig. B20 translates into improved metrics on 00/12 UTC initializations over 06/18 UTC initializations. Overall, the difference in assimilation windows used in our evaluation leaves ENS with a small advantage of up to 2 h additional look-ahead over the ML models, for all variables except sea surface temperature.

### ENS initialization and evaluation times

As discussed above, we evaluate GenCast only on forecasts initialized at 06/18 UTC, as using 00/12-initialized forecasts gives GenCast an additional advantage because of the longer data-assimilation look-ahead. Ideally, we would compare all models at the same 06/18 UTC initialization times. However, ENS forecasts from 06/18 UTC are archived only up to 6-day lead times and are not free for public download. Hence, we evaluate ENS on forecasts initialized at 00/12 UTC. For globally averaged metrics, this should not matter, and in fact ref. ^2^ found that 00/12 UTC initialization tends to give a small advantage in RMSE to the deterministic HRES forecast over the 06/18 UTC initialization, and we expect a similar minor advantage to apply to ENS. However, the regional wind power evaluation is sensitive to the diurnal cycle because wind power capacity is sparsely and non-uniformly distributed around the world. Thus, in this case, it is important to compare forecasts by ENS and GenCast at the same set of validity times. We, therefore, evaluate ENS (initialized at 00/12 UTC) at the same 06/18 UTC targets as GenCast. However, GenCast produces 06/18 UTC forecasts at lead times of 12 h, 24 h, 36 h and so on, whereas for ENS we obtain only 06/18 UTC forecasts at lead times of 6 h, 18 h, 30 h and so on. To estimate 06/18 UTC regional wind power CRPS of ENS at the same lead times as GenCast, we linearly interpolate the CRPS curve of ENS. In Supplementary Information section B.8.1, we validate this approach on 2018 data in which we did get access to ENS 06/18 UTC initializations, showing that this lead time interpolation overestimates the performance of ENS, in particular at short lead times.

## Online content

Any methods, additional references, Nature Portfolio reporting summaries, source data, extended data, supplementary information, acknowledgements, peer review information; details of author contributions and competing interests; and statements of data and code availability are available at 10.1038/s41586-024-08252-9.

## Supplementary information

Supplementary Information This file contains Supplementary Methods, Supplementary Results, Forecast Visualizations, and Supplementary References.

## Figures and Tables

**Extended Data Table 1:**
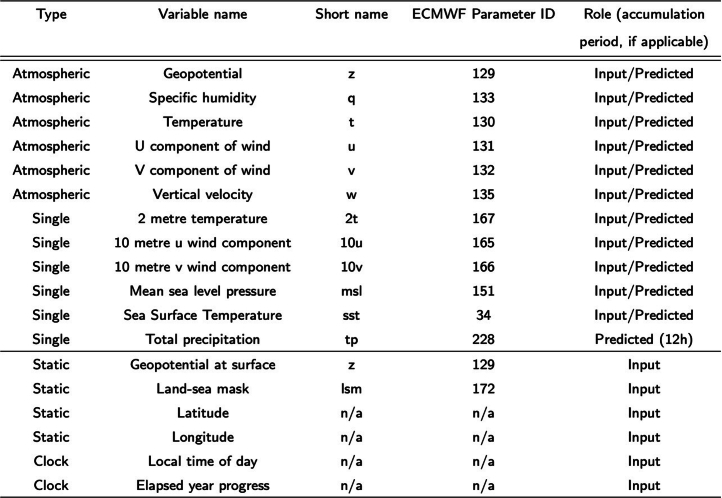
ECMWF variables used in our datasets

The “Type” column indicates whether the variable represents a static property, a time-varying single-level property (e.g. surface variables are included), or a time-varying atmospheric property. The “Variable name” and “Short name” columns are ECMWF’s labels. The “ECMWF Parameter ID” column is ECMWF’s numeric label, and can be used to construct the URL for ECMWF’s description of the variable, by appending it as suffix to the following prefix, replacing “ID” with the numeric code: https://apps.ecmwf.int/codes/grib/param-db/?id=ID. The “Role” column indicates whether the variable is something our model takes as input and predicts, or only uses as input context (the double horizontal line separates predicted from input-only variables, to make the partitioning more visible). For atmospheric variables, the 13 atmospheric pressure levels are taken as input and predicted by GenCast are: 50, 100, 150, 200, 250, 300, 400, 500, 600, 700, 850, 925, and 1000 hPa.

## Data Availability

The ERA5 dataset was downloaded and is downloadable from the Climate Data Store (CDS) of the Copernicus Climate Change Service (https://cds.climate.copernicus.eu). The results contain modified Copernicus Climate Change Service information 2020. Neither the European Commission nor ECMWF is responsible for any use that may be made of the Copernicus information or data it contains. ENS and HRES data were downloaded and are downloadable from the ECMWF as of April 2024 (https://apps.ecmwf.int/datasets/data/tigge/), and are usable according to the license described at https://apps.ecmwf.int/datasets/licences/tigge/. The data form part of the THORPEX Interactive Grand Global Ensemble (TIGGE) archive (https://confluence.ecmwf.int/display/TIGGE). TIGGE is an initiative of the World Weather Research Programme (WWRP). The Global Power Plant Database v.1.3.0 was and can be downloaded from https://datasets.wri.org/dataset/globalpowerplantdatabase. The idealized wind turbine power curve was and can be downloaded from the National Renewable Energy Laboratory https://github.com/NREL/turbine-models/blob/master/Onshore/ WTK_Validation_IEC-2_normalized.csv. IBTrACS (International Best Track Archive for Climate Stewardship) data usage policy follows the World Data Center for Meteorology (WDC), which provides full and open access to the data. IBTrACS cyclone tracks are available for download from https://www.ncei.noaa.gov/products/international-best-track-archive?name=ib-v4-access. Plots showing coastlines were generated using Matplotlib^69^ with Cartopy^70^.

## Code availability

Code implementing GenCast is available as part of the GitHub (https://github.com/google-deepmind/graphcast) repository (DOI: 10.5281/zenodo.14261754), which also provides download links for both ERA5 and operational versions of the model weights.
