# Supplementary material for: Probabilistic weather forecasting with machine learning
Source: Nature. 2024 Dec 4;637(8044):84–90. doi: 10.1038/s41586-024-08252-9 (PMC11666454; doi:10.1038/s41586-024-08252-9)
Supplement: Supplementary file 1 — This file contains Supplementary Methods, Supplementary Results, Forecast Visualizations, and Supplementary References. [file 41586_2024_8252_MOESM1_ESM.pdf]

nature portfolio

https://doi.org/10.1038/s41586-024-08252-9

Supplementary information

Probabilistic weather forecasting with machine learning

In the format provided by the authors and unedited

Probabilistic weather forecasting with machine learning (Supplementary Information)

Ilan Price,* Alvaro Sanchez-Gonzalez,* Ferran Alet,* Tom R. Andersson,*
Andrew El-Kadi, Dominic Masters, Timo Ewalds, Jacklynn Stott,
Shakir Mohamed, Peter Battaglia, Remi Lam, Matthew Willson

*Equal contributions
Corresponding authors:
Ilan Price (pricei@google.com), Matthew Willson (matthjw@google.com),
Remi Lam (remilam@google.com), Peter Battaglia (peterbattaglia@google.com)

A Supplementary methods **3**
A.1 Data . . . 3
A.1.1 ERA5 . . . 3
A.1.2 Data preprocessing. . . . 3
A.2 Diffusion settings . . . 4
A.2.1 Sampler hyperparameters . . . 4
A.2.2 Training-time noise-level distribution . . . 4
A.2.3 Noise distribution on the sphere . . . 4
A.3 Optimiser hyperparameters . . . 5
A.4 Perturbed initial conditions for GenCast-Perturbed . . . 6
A.5 Verification metrics . . . 6
A.5.1 CRPS . . . 7
A.5.2 Ensemble mean RMSE . . . 7
A.5.3 Spread/skill ratio . . . 8
A.5.4 Rank histogram . . . 8
A.5.5 Brier skill score . . . 8
A.5.6 Relative Economic Value . . . 9
A.6 Statistical tests . . . 11
A.6.1 Test for deterministic cyclone position errors . . . 12
A.7 Tropical cyclone evaluation . . . 13
A.7.1 Deterministic vs. probabilistic evaluation . . . 13
A.7.2 Use of TempestExtremes for both forecasts and ground truth . . . 13
A.7.3 TempestExtremes tracker details . . . 14
A.8 Pooled evaluation details . . . 15
A.9 Regional wind power evaluation . . . 15

B Supplementary results **17**
B.1 Ensemble calibration . . . 17
B.2 Precipitation . . . 18
B.3 Spectrum for additional variables . . . 20
B.4 GenCast-Perturbed scorecards . . . 23
B.5 Tropical cyclones . . . 24
B.5.1 Deterministic evaluation . . . 24
B.5.2 Probabilistic evaluation . . . 25
B.6 Pooled evaluation . . . 28
B.7 Regional wind power forecasting statistical significance . . . 32
B.8 Empirical support of initialisation time and evaluation time choices for verification . . . 33
B.8.1 Lead time interpolation in regional wind farm evaluation . . . 33
B.8.2 Initialisations used for GenCast evaluation . . . 33

C Forecast visualisations **35**
C.1 Tropical cyclone tracks . . . 35
C.2 Global forecasts . . . 40

Appendix A Supplementary methods

A.1 Data

A.1.1 ERA5

The following description of the ERA5 dataset is adapted directly from (2). ECMWF's ERA5 (27)[1] archive is a large corpus of data that represents the global weather from 1959 to the present, at 1 hour increments, for hundreds of static, surface, and atmospheric variables. The ERA5 archive is based on *reanalysis*, which uses ECMWF's Integrated Forecast System (IFS) cycle 42r1 that was operational for most of 2016, with an ensemble 4D-Var data assimilation scheme. ERA5 assimilated 12-hour windows of observations, from 21-09 UTC and 09-21 UTC, as well as previous forecasts, into a dense representation of the weather's state, for each historical date and time. ERA5 is computed natively at 31 km (approximately 0.281 25°) resolution. We used the version available in the Climate Data Store (CDS) of the Copernicus Climate Change Service, which has been regridded to a 0.25° equiangular latitude/longitude grid.

Our ERA5 dataset contains a subset of available variables in ECMWF's ERA5 archive (Extended Data Table 1), on 13 pressure levels[2] corresponding to the levels of the WeatherBench (71) benchmark: 50, 100, 150, 200, 250, 300, 400, 500, 600, 700, 850, 925, and 1000 hPa. The range of years included was 1979-01-01 to 2022-01-10, which were downsampled to 12 hour time intervals (corresponding to 00:00, 06:00, 12:00 and 18:00 UTC each day). The downsampling is performed by subsampling, except for the total precipitation, which is accumulated for the 12 hours leading up to the corresponding downsampled time.

A.1.2 Data preprocessing.

Handling of ENS missing data. When trying to download ENS forecasts for surface variables for the 2019-10-17 00:00 UTC initialisation, we were met with persistent errors, so we left that initialisation out of the ENS evaluation, and used neighboring information when performing paired statistical tests for differences in verification metrics (see Section A.6 for details).

Variables with NaNs. ERA5 sea surface temperature (SST) data contains NaNs over land by default. As preprocessing of the SST training and evaluation data, values over land are replaced with the minimum sea surface temperature seen globally in a subset of ERA5.

[1] See ERA5 documentation: `https://confluence.ecmwf.int/display/CKB/ERA5`.

[2] We follow common practice of using pressure as our vertical coordinate, instead of altitude. A "pressure level" is a field of altitudes with equal pressure. E.g., "pressure level 500 hPa" corresponds to the field of altitudes for which the pressure is 500 hPa. The relationship between pressure and altitude is determined by the geopotential variable.

A.2 Diffusion settings

A.2.1 Sampler hyperparameters

To draw samples we use DPMSolver++2S (53) as a drop-in replacement for the second-order Heun solver used in (21). It is also a second-order ODE solver requiring $2N-1$ function evaluations for N noise levels (two per step, one fewer for the final Euler step). We augment it with the stochastic churn and noise inflation described in Algorithm 2 of (21).

At sampling time we adopt the noise level schedule specified by (21):

$$\sigma_i := \left(\sigma_{max}^{\frac{1}{\rho}} + \frac{i}{N-1}(\sigma_{min}^{\frac{1}{\rho}} - \sigma_{max}^{\frac{1}{\rho}})\right)^{\rho} \quad \text{for } i \in \{0 \dots N-1\}.$$

Settings for the parameters of this schedule ($\rho, \sigma_{min}, \sigma_{max}$, N) as well for stochastic churn and noise inflation are given in Table A1.

Name	Notation	Value, sampling	Value, training
Maximum noise level	σ_{max}	80	88
Minimum noise level	σ_{min}	0.03	0.02
Shape of noise distribution	ρ	7	7
Number of noise levels	N	20	
Stochastic churn rate	S_{churn}	2.5	
Churn maximum noise level	S_{tmax}	80	
Churn minimum noise level	S_{tmin}	0.75	
Noise level inflation factor	S_{noise}	1.05	

Table A1: **Settings used at sampling time, and their equivalents at training time where applicable**. Notation aligns with that used in (21).

A.2.2 Training-time noise-level distribution

At training time we construct a continuous distribution for noise levels, whose quantiles match the noise level schedule described above. Specifically its inverse CDF is:

$$F^{-1}(u) = \left(\sigma_{max}^{\frac{1}{\rho}} + u(\sigma_{min}^{\frac{1}{\rho}} - \sigma_{max}^{\frac{1}{\rho}})\right)^{\rho}$$

and we sample from it by drawing $u \sim U[0,1]$. At training time we use the same ρ as at sampling time, but a slightly wider range for $[\sigma_{min}, \sigma_{max}]$, values are in Table A1.

A.2.3 Noise distribution on the sphere

Much of the theory around diffusion models is developed for the case of Gaussian white noise. A true white noise process on the sphere is isotropic or rotation-invariant, and

is characterised by a flat spherical harmonic power spectrum in expectation. However these properties do not hold if we attempt to approximate it at finite resolution by sampling i.i.d. noise on the cells of our equiangular latitude-longitude grid. This is due to the greater density of cells near to the poles which results in more power at higher frequencies in the spherical harmonic domain.

Empirically we didn't find this to be a fatal problem; nonetheless we found we can obtain a small but consistent improvement using a different approach to noise sampling which is sensitive to the spherical geometry. We sample isotropic Gaussian noise in the spherical harmonic domain, with an expected power spectrum that is flat over the range of wavenumbers that our grid is able to resolve, and truncated thereafter. We then project it onto our discrete grid using the inverse spherical harmonic transform (72). The resulting per-grid-cell noise values are not independent especially near to the poles, but are approximately independent at the resolution resolved at the equator, and display the desired properties of isotropy and flat power spectrum.

A.3 Optimiser hyperparameters

Optimiser	AdamW (73)
LR decay schedule	Cosine
Stage 1: Batch size	32
Stage 1: Warm-up steps	1e3
Stage 1: Total train steps	2e6
Stage 1: Peak LR	1e-3
Stage 1: Weight decay	0.1
Stage 2: Batch size	32
Stage 2: Warm-up steps	5e3
Stage 2: Total train steps	64000
Stage 2: Peak LR	1e-4
Stage 2: Weight decay	0.1

Table A2: Diffusion model training hyperparameters.

Optimiser	AdamW
LR decay schedule	Cosine
Batch size	32
Warm-up steps	1e3
Total train steps	3e5
Peak LR	1e-3
Weight decay	0.1

Table A3: GenCast-Perturbed model training hyperparameters.

A.4 Perturbed initial conditions for GenCast-Perturbed

To initialise GenCast-Perturbed, we take two consecutive deterministic ERA5 analysis states and add to them perturbations sampled from a zero-mean Gaussian process on the sphere. This process uses the Gaussian-like stationary isotropic correlation function from (74), with a horizontal decorrelation length-scale of 1200km.

Geopotential
Temperature
U component of wind
V component of wind
2-metre temperature

Table A4: Variables to which Gaussian process perturbations are applied

We sample independent perturbations for each of the variables listed in Table A4; other variables are not perturbed by the Gaussian process. We obtained better results perturbing only this subset than the full set of input variables, although we have not exhaustively investigated the best subset of variables to perturb.

The marginal standard deviations of the perturbations are equal to 0.1 times those of 6-hour differences in the corresponding variables at each respective pressure (or surface) level. Aside from these differences in scale, for a given variable the same perturbation is used for all levels, and for both input timesteps. This is equivalent to infinite vertical and temporal decorrelation lengthscales. We investigated using lower vertical decorrelation lengthscales but found they did not help.

We selected the scale factor 0.1 and horizontal decorrelation length-scale 1200km based on CRPS scores for the resulting forecasts over a range of variables and lead times, after sweeping over scale factors $(0.03, 0.05, 0.07, 0.085, 0.1, 0.3)$, and decorrelation lengthscales (30km, 480km, 1200km, 3000km). Results were significantly worse at the shortest lengthscale we tried of 30km, but were not otherwise very sensitive to decorrelation lengthscale.

These perturbations are quite crude; in particular they are not flow-dependent and do not take any care to preserve physical invariants. Nevertheless we've found them to be surprisingly effective at longer lead times with the deterministic model using the GenCast architecture.

A.5 Verification metrics

In the following, for a particular variable, level and lead time,

- $x^m_{i,k}$ denotes the value of the mth of M ensemble members in a forecast from initialisation time indexed by $k = 1 \dots K$, at latitude and longitude indexed by $i \in G$.

- $y_{i,k}$ denotes the corresponding verification target.
- $\bar{x}_{i,k} = \frac{1}{M}\sum_m x_{i,k}^m$ denotes the ensemble mean.
- $S_{i,k}^2 = \frac{1}{M-1}\sum_m (x_{i,k}^m - \bar{x}_{i,k})^2$ denotes the unbiased estimate of ensemble variance.
- a_i denotes the area of the latitude-longitude grid cell, which varies by latitude and is normalized to unit mean over the grid.

A.5.1 CRPS

We estimate the Continuous Ranked Probability Score (CRPS, see e.g. (31)) for ensemble forecasts using the fair CRPS estimator of (75). Here 'fair' (76) means that it is an unbiased estimator of the CRPS of the underlying predictive distribution from which the ensemble was sampled.

$$\mathrm{CRPS}_{fair} := \frac{1}{K}\sum_k \frac{1}{|G|}\sum_i a_i \Big(\frac{1}{M}\sum_m |x_{i,k}^m - y_{i,k}| - \frac{1}{2M(M-1)}\sum_{m,m'} |x_{i,k}^m - x_{i,k}^{m'}| \Big). \tag{A1}$$

For CRPS, smaller is better.

A.5.2 Ensemble mean RMSE

The mean squared error of the ensemble mean:

$$\mathrm{EnsembleMeanMSE} := \frac{1}{K}\sum_k \frac{1}{|G|}\sum_i a_i \left(\bar{x}_{i,k} - y_{i,k}\right)^2, \tag{A2}$$

is a biased estimator of the MSE of the mean of the underlying predictive distribution (20, Appendix F.1), and hence unfair in the sense of (76). For consistency with our treatment of CRPS, we bias-correct it to obtain a fair ensemble mean MSE:

$$\mathrm{EnsembleMeanMSE}_{fair} := \mathrm{EnsembleMeanMSE} - \mathrm{MeanEnsembleVariance}/M \tag{A3}$$

where

$$\mathrm{MeanEnsembleVariance} := \frac{1}{K}\sum_k \frac{1}{|G|}\sum_i a_i S_{i,k}^2 \tag{A4}$$

is the mean unbiased estimate of ensemble variance. We then report the square root of this as our RMSE:

$$\mathrm{EnsembleMeanRMSE} := \sqrt{\mathrm{EnsembleMeanMSE}_{fair}}. \tag{A5}$$

A.5.3 Spread/skill ratio

We report the following spread/skill ratio:

$$\text{SpreadSkillRatio} := \sqrt{\frac{\text{MeanEnsembleVariance}}{\text{EnsembleMeanMSE}_{fair}}}, \tag{A6}$$

following (32) in using the root mean estimate of ensemble variance as our measure of spread. Under the assumption of perfect forecasts where ensemble members and ground truth $\{x^1_{i,k}, \ldots, x^M_{i,k}, y_{i,k}\}$ are all exchangeable, we find that

$$\mathbb{E}[\text{EnsembleMeanMSE}_{fair}] = \mathbb{E}[\text{MeanEnsembleVariance}], \tag{A7}$$

and thus in the perfect case SpreadSkillRatio ≈ 1. While diagnosis of under- or over-dispersion is confounded by forecast bias (77; 78), if we assume such bias is relatively small, we can associate under-dispersion on average with spread/skill < 1 and over-dispersion on average with spread/skill > 1.

A.5.4 Rank histogram

The rank histogram (33) measures where the ground truth value tends to fall with respect to the ensemble distribution. More precisely, for each evaluation time and for each grid cell, we record the rank of the ground truth among the forecast ensemble members, from 1 to $M+1$, where M is the ensemble size, and plot the histogram of these ranks. For perfect ensemble forecasts in which ensemble members and ground truth are all exchangeable, we expect to see a flat rank histogram, since the true value should fall between any pair of proximal sorted ensemble values with equal probability. If we assume relatively small bias, rank histograms can be used to diagnose under- or over-dispersion (77; 78). Under-dispersion will tend to result in the rank of the ground truth falling towards or beyond the outer bounds of the ensembles' values, resulting in a rank histogram with a $\cup$-shape. Conversely, an over-dispersed but unbiased ensemble will result in the ground truth falling predominantly near the center of the range of ensemble values, causing the rank histogram to have a $\cap$-shape. Peaks on either side of the rank histogram can suggest over– or under- prediction bias.

A.5.5 Brier skill score

Suppose now that predictions $x^m_{i,k} \in \{0, 1\}$ and targets $y_{i,k} \in \{0, 1\}$ are binary variables, such as whether or not one is close to a cyclone, or whether surface temperature exceeds the 99.9th percentile of climatology. Our ensemble means $\bar{x}_{i,k}$ now correspond to empirical predictive probabilities for the events in question, and the ensemble mean MSE of Equation (A2) corresponds to the Brier score (79) for these probabilities.

$$\text{BrierScore} := \text{EnsembleMeanMSE} \tag{A8}$$

As with the ensemble mean MSE, this is a biased estimate of the Brier score of the underlying predictive probability. Instead we use the fair / bias-corrected version from Equation (A3), which corresponds to the adjusted Brier score of (76; 80):

$$\text{BrierScore}_{fair} := \text{EnsembleMeanMSE}_{fair} \tag{A9}$$

To obtain a Brier skill score, this is then normalized relative to the Brier score attained by predicting a fixed (and location-independent) climatological probability of the event, estimated using the evaluation set:

$$p_{clim} := \frac{1}{K}\sum_k \frac{1}{|G|}\sum_i a_i y_{i,k} \tag{A10}$$

$$\text{BrierScore}_{clim} := \frac{1}{K}\sum_k \frac{1}{|G|}\sum_i a_i \left(p_{clim} - y_{i,k}\right)^2 \tag{A11}$$

$$\text{BrierSkillScore} := 1 - \frac{\text{BrierScore}_{fair}}{\text{BrierScore}_{clim}} \tag{A12}$$

Brier skill score is 0 for the climatological forecast and 1 for a perfect forecast, so larger is better.

A.5.6 Relative Economic Value

Relative Economic Value curves have been proposed by (37; 38; 81), based on the well-studied cost-loss ratio decision model (36; 82; 83) for forecasts of binary events. This model allows us to assess the value of a forecast to a range of users facing different decision problems—from those who will act on a relatively small probability of a severe event, to those who will only act once the event is predicted with confidence.

In this model, a user must decide whether or not to prepare for an adverse weather event. Facing the event unprepared incurs an expense L (the 'loss'). However, this loss can be avoided by preparing for the event with an expense C (the 'cost'), as reflected in Table A5:

	Event doesn't happen	Event happens
No preparation made	0	L
Preparation made	C	C

Table A5: **Expenses under the cost-loss decision model.**

The optimal strategy is to take action whenever the probability of the event exceeds C/L. This 'cost-loss ratio' is thus sufficient to characterise the decision problem faced by a particular user. Since it may vary significantly for different users, we display results for a range of cost-loss ratios. For severe weather events we will focus in particular on small cost-loss ratios (on the order of 0.01 to 0.2 for example) since these are more typical in practice, see (35) and references therein for a number of examples.

Under this model the expected expense of a decision system can be computed in terms of its confusion matrix, consisting of the proportions of true and false positives and negatives incurred (denoted TP, TN, FP, FN).

To make binary decisions based on our ensemble, we select a probability threshold q, and make a positive prediction whenever our empirical predictive probability $\bar{x}_{i,m}$ exceeds q, obtaining binary forecasts:

$$x_{i,k} := \mathbb{I}[\bar{x}_{i,k} > q]. \tag{A13}$$

We then compute the confusion matrix weighted by grid cell area:

$$\begin{bmatrix} \mathrm{TN} & \mathrm{FN} \\ \mathrm{FP} & \mathrm{TP} \end{bmatrix} := \frac{1}{K} \sum_k \frac{1}{|G|} \sum_i a_i \begin{bmatrix} (1 - x_{i,k})(1 - y_{i,k}) & (1 - x_{i,k}) y_{i,k} \\ x_{i,k}(1 - y_{i,k}) & x_{i,k} y_{i,k} \end{bmatrix} \tag{A14}$$

Multiplying the entries of this confusion matrix with the expenses in Table A5 and summing, we can then compute the expected expense incurred as:

$$E_{forecast} = (\mathrm{TP} + \mathrm{FP}) \cdot C + \mathrm{FN} \cdot L. \tag{A15}$$

To derive relative economic value (REV), we normalize this relative to the expense E_{clim} incurred by a constant forecast (whose value is chosen based only on the climatological base rate of the event), and the expense $E_{perfect}$ incurred by a perfect forecast. In other words, REV compares how much expense you save using the forecasts instead of relying on climatology, relative to how much it would have been possible to save if you had the perfect forecast.

Never preparing will incur expense equal to either $(\mathrm{TP}+\mathrm{FN}) \cdot L$ (because $\mathrm{TP}+\mathrm{FN}$ is the base rate of the event), whereas always preparing will incur an expense C. Choosing the better of these strategies yields

$$E_{clim} = \min\{(\mathrm{TP} + \mathrm{FN}) \cdot L, C\}, \tag{A16}$$

while with a perfect forecast, one only needs to prepare when the event is actually going to occur, incurring an expense

$$E_{perfect} = (\mathrm{TP} + \mathrm{FN}) \cdot C. \tag{A17}$$

REV is then defined as

$$\mathrm{REV}(C/L, q) := \frac{E_{clim} - E_{forecast}}{E_{clim} - E_{perfect}}, \tag{A18}$$

and is 0 for a forecast based on climatology alone and 1 for a perfect forecast, so larger is better. Note that by dividing each term in Equation (A18) by L, the definition of REV depends on C and L only through the cost-loss ratio C/L.

It also depends on the probability threshold q that was chosen earlier. When probabilities are perfectly calibrated, it is optimal to set $q = C/L$. In practise ensemble

forecast systems are rarely perfectly calibrated, and we are more interested in the potential REV obtainable by a system after its probabilities have been optimally recalibrated. We follow (37) in approximating this by computing REV at every possible threshold for the empirical predictive probability of our size-M ensemble, and taking the maximum REV over these:

$$\text{REV}^*(C/L) := \max_{j=0,\dots,M+1} \text{REV}\Big(C/L, q = \frac{j - 1/2}{M}\Big) \tag{A19}$$

It is this maximum or potential REV that we report throughout; for the sake of conciseness we refer to it just as REV. Note that it is impossible to do worse than climatology on this metric, since both options available to the climatological forecast (never preparing and always preparing) are included in the maximization above. Thus $0 \leq \text{REV}^* \leq 1$ always holds, and it is common to see values equal to zero when a forecast does not improve on climatology.

A.6 Statistical tests

As outlined in 'Statistical methods' in Methods, for key verification metrics we test the null hypothesis of no difference in the metric between GenCast and ENS, against the two-sided alternative.

With the exception of deterministic cyclone position errors (treated separately in Section A.6.1), each of our verification metrics V can be viewed as a function of the mean of a time-series of statistics given for every initialisation time t, where these statistics have already been spatially averaged. The statistics used for each metric are given in Table A6.

Verification metric	Statistic time-series	Proxy used for block length selection
CRPS	CRPS	CRPS
Ensemble mean RMSE	Ensemble mean MSE	Ensemble mean MSE
Brier skill score	Brier score, base rate	BSS with constant denominator
REV*	Confusion matrix	REV with constant denominator and q maximizing overall REV.

Table A6: **Time-series of statistics used by each verification metric.**

To perform our test, we compute paired time-series of these statistics for both GenCast and ENS. We then resample the paired time-series 10000 times using the stationary block bootstrap of (58) implemented in (84). We compute $V_{GenCast} - V_{ENS}$ for each resample, and use these values to construct a $(1 - \alpha) * 100\%$ confidence interval for the difference using the bias-corrected and accelerated ('bca') method of (62). We reject the null hypothesis when this interval does not contain zero.

Block length selection In order to account for temporal dependence it is important to select an appropriate mean block length for the stationary block bootstrap. For this we

use the automatic block length selection described in (59; 60) and implemented by (84). Block length selection is performed separately for each lead time, variable and level, and for each setting of the metric in question (such as cost-loss ratio or event threshold). Since the selection mechanism takes as input a univariate time-series and our paired time-series of statistics are multivariate, we compute a suitable per-initialisation-time proxy V_k whose temporal mean is equal to or closely related to V, described in Table A6. We then use differences $V_{GenCast,k} - V_{ENS,k}$ for block length selection.

Alignment of GenCast and ENS time-series As motivated in 'ENS initialization and evaluation times' in Methods, ENS is initialised at 00/12 UTC, and GenCast at 06/18 UTC, meaning that forecast initialisation times do not match between the two time-series of statistics. In order to align the two time-series when performing a paired test, we take one of two approaches:

- Pair statistics for GenCast with statistics for ENS that are based on a forecast initialisation time 6 hours earlier, and a validity time 6 hours earlier too (so maintaining the same lead time).
- Pair statistics for GenCast with the mean of two statistics for ENS: one taken from a forecast initialised 6 hours earlier and so with 6 hours additional lead time; another taken from a forecast initialised 6 hours later and so with 6 hours less lead time. Both forecasts thus have the same validity time as the corresponding GenCast forecast, and the lead time is the same on average. This method is used only in the results on regional wind power forecasting, where a similar adjustment is performed for the reported metrics themselves; these adjustments are described and motivated in more detail in 'ENS initialization and evaluation times' in Methods.

Missing data. For ENS, a single initialisation time is missing, as described in Section A.1.2. The statistics from Table A6 are imputed for this initialisation time based on a linear interpolation of the previous and next values.

A.6.1 Test for deterministic cyclone position errors

For a given cyclone, position errors are obtained at validity times and forecast initialisation times for which the pairing criteria described in 'Cyclone position error evaluation' in Methods are met. For each lead time we wish to test for a difference in mean position error, where the mean is taken over all paired forecast initialisation times for all cyclones.

Similarly to (2) we assume independence between, but not within, cyclones. We perform a cluster bootstrap (61, pp.100-101, 'Strategy 1') with clusters corresponding to cyclones, meaning that we resample the dataset at the cyclone level, but retain the full, original set of paired forecast initialisations for each cyclone that is resampled. Our test, like the others above, is based on bias-corrected and accelerated confidence intervals (62) derived from these resampled datasets.

A.7 Tropical cyclone evaluation

Here we provide a comparison between our two cyclone evaluations, motivation behind our choice of ground truth, and further cyclone tracker details.

A.7.1 Deterministic vs. probabilistic evaluation

As discussed in both the Main and Methods, we evaluate GenCast and ENS's cyclone forecasting skill in both a deterministic and probabilistic setting, via ensemble mean position error (Figure 4b) and strike probability (Figure 4c). Deterministic position error is a common cyclone verification method (48) and is measured in intuitive units (kilometers), but comes with a number of caveats and limitations:

1. It requires pairing forecast cyclone trajectories with cyclones existing at initialisation, and thus does not measure ability to capture cyclogenesis events,
2. For probabilistic ensemble forecasts it requires computing the ensemble mean cyclone track and thus disregards spread and uncertainty in the forecasts,
3. It can only be computed when the forecast cyclone and true cyclone both exist, and thus does not penalise underprediction or overprediction of the cyclone's duration,
4. When comparing the average position error of GenCast and ENS, it requires both models to predict a cyclone as existing at a given lead time, and removes cases where only one of the models (correctly) predicts the cyclone's existence.

Our strike probability (48) evaluation is probabilistic (avoiding limitation 2). It also does not require pairing of forecast and ground truth cyclones (overcoming limitation 1 and 3), and does not require computing the intersection of valid forecasts across models (overcoming limitation 4). Evaluating strike probability therefore penalises incorrectly predicting the existence or non-existence of a cyclone as well as errors in predictions of their positions, representing a more robust and challenging evaluation of cyclone forecasting skill.

A.7.2 Use of TempestExtremes for both forecasts and ground truth

Different cyclone trackers apply different detection cyclone detection criteria, which can affect results substantially, confounding the evaluation of the accuracy of the underlying gridded predictions (63; 85). We control for this in our cyclone evaluation through our use of the TempestExtremes cyclone tracker. We apply this same tracker in the same way to all models and ground truth analysis datasets (without tuning the tracker to optimise performance on a particular model), which ensures the same definition of a cyclone is used throughout the evaluation ('Tropical cyclone evaluation' in Methods). Our evaluation therefore minimises bias toward any particular model, isolating the relative quality of the raw forecasts (as opposed to the quality or sensitivity of the tracker or source of ground truth).

A.7.3 TempestExtremes tracker details

The TempestExtremes tracker works in two stages. An overview of these two stages is provided below, using default tracker hyperparameter values where mentioned.

The first stage, *DetectNodes*, finds candidate tropical cyclones where minima in mean sea level pressure (MSL) are co-located with coherent upper-level warm cores:

- Initial candidate locations are determined by local minima in MSL. Candidates within 6° of another stronger MSL minimum are eliminated.
- Each MSL minimum must be surrounded by a closed contour of MSL that is 200 hPa greater than the minimum. The minima must be sufficiently compact with this closed contour falling within a 5.5° great circle distance of the MSL minimum.
- A warm core criterion checks that candidate storms are co-located with maxima in the geopotential thickness field between 500 hPa and 300 hPa, Z500 - Z300. The maxima must be enclosed by a closed contour of thickness that is 58.8 $\mathrm{m^2s^{-2}}$ less than the maximum. The maxima must be sufficiently compact with this closed contour falling within a 6.5° great circle distance from the maximum.

The second stage, *StitchNodes*, detects plausible cyclone trajectories from these candidate cyclone locations by linking them together in time with the following criteria:

- Candidates cannot move more than an 8° great circle distance between subsequent time slices (the 'stitch range').
- Trajectories are allowed a maximum gap of 24 hours between candidate nodes; greater gaps result in a trajectory terminating (and potentially a new trajectory forming).
- To filter out short-lived storms that are not tropical cyclones, trajectories must last for at least 54 hours.
- A threshold analysis is performed on candidate trajectories to ensure cyclones are sufficiently intense and in the right locations on Earth: each trajectory must have at least 10 time slices with wind speed greater than 10 $\mathrm{ms^{-1}}$, elevation below 150 m, and latitude between $-50°$ and 50°.

The algorithm's default hyper-parameters were chosen so that when TempestExtremes is applied to 6-hourly analysis datasets the resulting tracks closely match observed tracks (66), using the IBTrACS dataset as ground truth (63). To account for the 12-hourly (instead of 6-hourly) temporal resolution in our evaluation, we changed two of the tracker's *StitchNodes* hyperparameters. Firstly, we halved the number of time slices for the criteria checks from 10 to 5. Secondly, because cyclones can travel further in a 12 hour interval than in 6 hours, we increased the stitch range from an 8° to a 12° great circle distance. This value was chosen from visual inspection to trade-off fast-moving cyclones being cut off (if the stitch range is too small) and trajectories jumping between separate storms (if the stitch range is too large). Increasing the stitch range to 12° can sometimes result in nearby but separate storms being connected as part of the same cyclone

trajectory (for example, in Figure C4g). However, we found this to be rare, and do not expect it to bias the results towards one particular model. All other TempestExtremes hyperparameters were left as their default values, and the same hyperparameters were used for each model and each analysis dataset.

A.8 Pooled evaluation details

The meteorological literature has produced a wide range of methods to verify spatial structure in weather forecasts (86). We use a neighbourhood (or 'fuzzy') approach, where forecasts and targets are first aggregated over regions of a particular spatial scale and standard skill scores computed on these pooled counterparts, with the process repeated at a range of spatial scales (39; 87). Neighbourhood methods often perform pooling using squares on a 2D latitude-longitude grid, which can lead to undesirable behaviour towards the poles and is not appropriate for evaluating global weather forecasts. We perform pooling on the surface of a sphere, sampling pool centres approximately uniformly over the Earth's surface with icosahedral meshes and using pooling regions within a fixed geodesic distance of each pool centre ('Spatially pooled CRPS evaluation' in Methods).

Our choice of six pooling sizes vary from mesoscales ($\sim$100s of km) to planetary scales ($>$2000 km). We use two standard aggregation methods from the neighbourhood verification literature: average-pooling and max-pooling, which assess how well the models predict statistics of the spatial distributions.

Average-pooling behaves like a low-pass filter, focusing the evaluation on increasingly large spatial structures as pooling size increases, and reducing the double-penalty problem at scales smaller than the pooling size.

Max-pooling emphasises the positive tail of the spatial distribution, which may include high-impact events such as extreme wind or temperature that would otherwise be blurred by average-pooling. In some cases, forecast users perform max-pooling to account for possible location error, such as the location of a weather front for wind power ramp prediction (88).

A.9 Regional wind power evaluation

Figure A1 shows the power curve used to convert from wind speed to load factor.

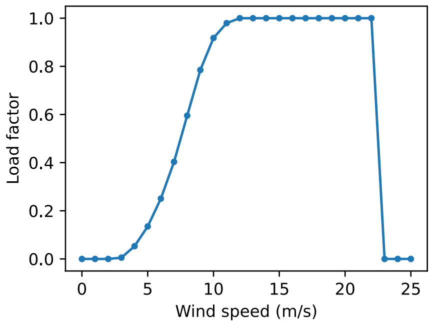

Figure A1: International Electrotechnical Commission Class II turbine power curve from the WIND Toolkit (67).

Appendix B Supplementary results

B.1 Ensemble calibration

We find that, generally, GenCast's spread/skill and rank histograms tend to be as good as ENS's and much better than that of GenCast-Perturbed. Figure B1 compares spread/skill scores across lead times for a set of different variables and pressure levels. GenCast exhibits spread-skill scores close to 1, with a marginal tendency towards minimal underdispersion. ENS's spread/skill ratio is also mostly quite close to one, with some exceptions like 2t, where it exhibits some under-dispersion. GenCast-Perturbed shows substantial under-dispersion across almost all variables and lead times.

Like those shown in Extended Data Figure 2, the rank histograms in Figure B2 confirm that GenCast generally has very flat rank histograms, in many cases flatter than those of ENS, and in almost all cases substantially flatter than GenCast-Perturbed.

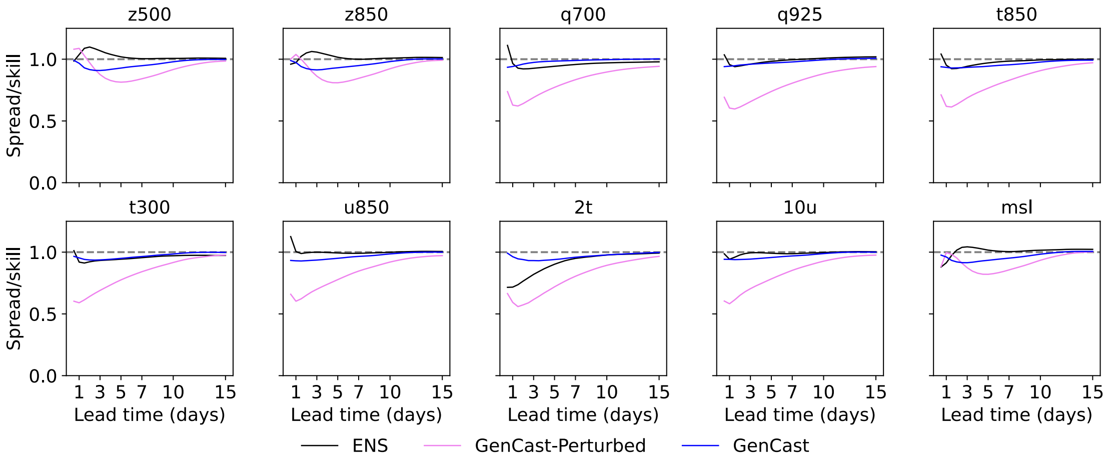

Figure B1: **Spread/skill shows that GenCast 's ensembles are well calibrated.** Spread/skill ratio model comparison for z500, z850, q700, q925, t850, t300, u850, 2t, 10u and msl.

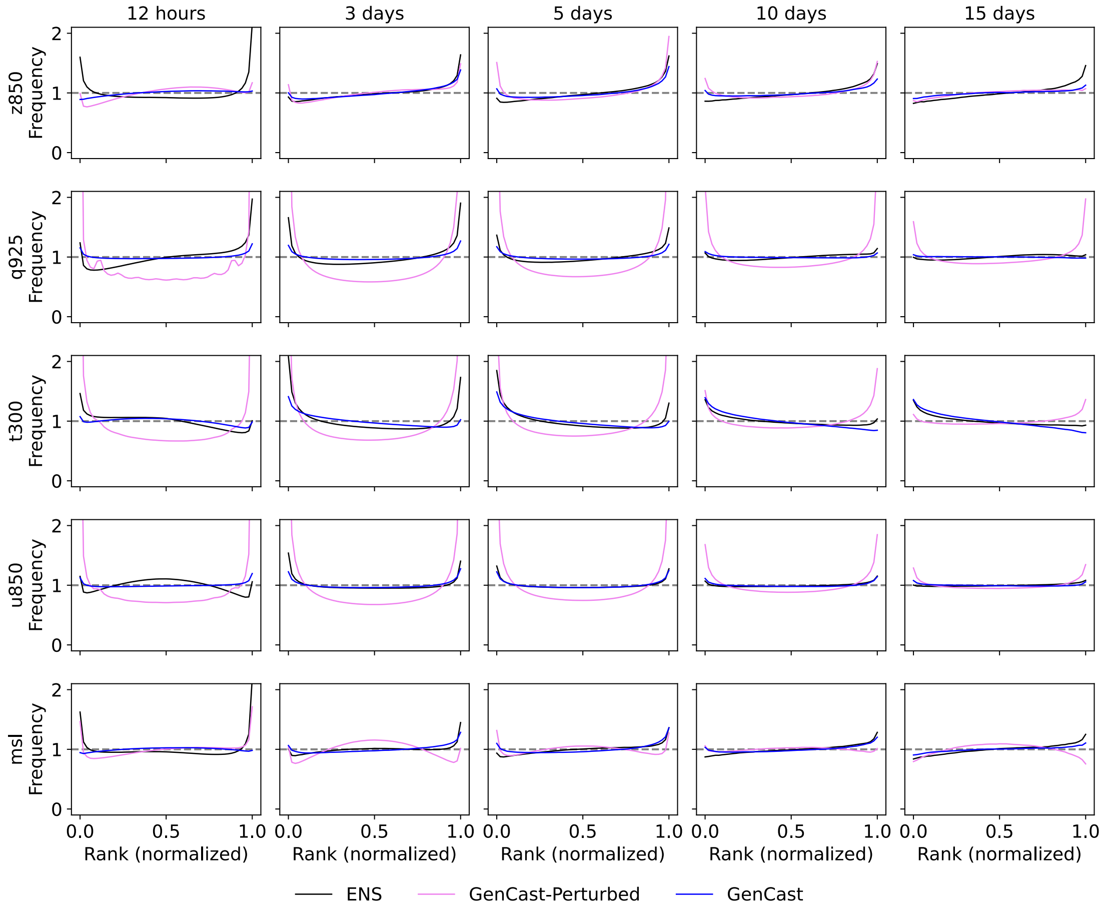

Figure B2: **Rank histograms show that GenCast 's ensembles are well calibrated.** Rank histogram model comparison at different lead times for z850, q925, t300, u850, and msl.

B.2 Precipitation

In line with the rest of the paper, we evaluate precipitation forecasting skill using RMSE, CRPS and rank histograms. Since some of these metrics may not be representative of actual skill due to precipitation being highly sparse and very non-Gaussian, we also evaluated it using Stable Equitable Error in Probability Space (SEEPS) (89–91), using the same methodology as in (2). Because SEEPS is a metric for deterministic categorical forecasts (dry, light-rain, and heavy-rain) we evaluated the ensembles both computing the category of the ensemble mean, as well as the mode of the ensemble category members (i.e. majority vote). We repeat the caveats mentioned in the main text that we lack full confidence in the quality of ERA5 precipitation data, and that we have not tailored our evaluation to precipitation specifically beyond adding SEEPS as a further metric. Results for 24 hour and 12 hour accumulated precipitation are shown in Figure B3 and

Figure B4 respectively.

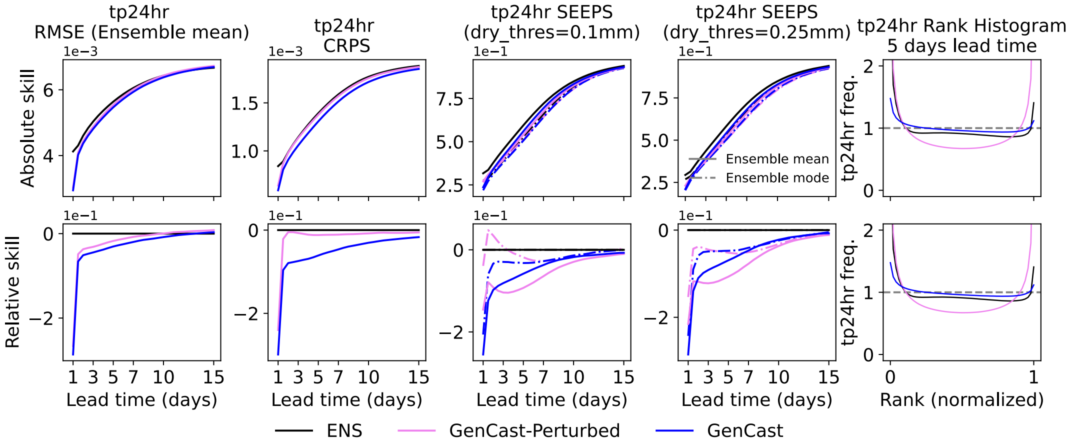

Figure B3: **Preliminary results on 24h accumulated precipitation show GenCast outperforming ENS.** Comparing preliminary results of model performance on predicted total accumulated precipitation over 24 hours, evaluated on Ensemble-Mean RMSE, CRPS, SEEPS with dry thresholds 0.1 and 0.25, and via rank-histogram. All metrics were calculated globally and over the full test period, except for SEEPS which excludes very dry regions according to the criteria in (2). For ensembles, we evaluate SEEPS both on the mean of the ensemble, as well as using the mode of the dry, light-rain, and heavy-rain categories predicted by the different ensemble members (i.e. majority vote). Relative and absolute plots are shown in the top and bottom rows respectively. GenCast shows promising results, almost always outperforming ENS (4) and with a significantly flatter rank histogram.

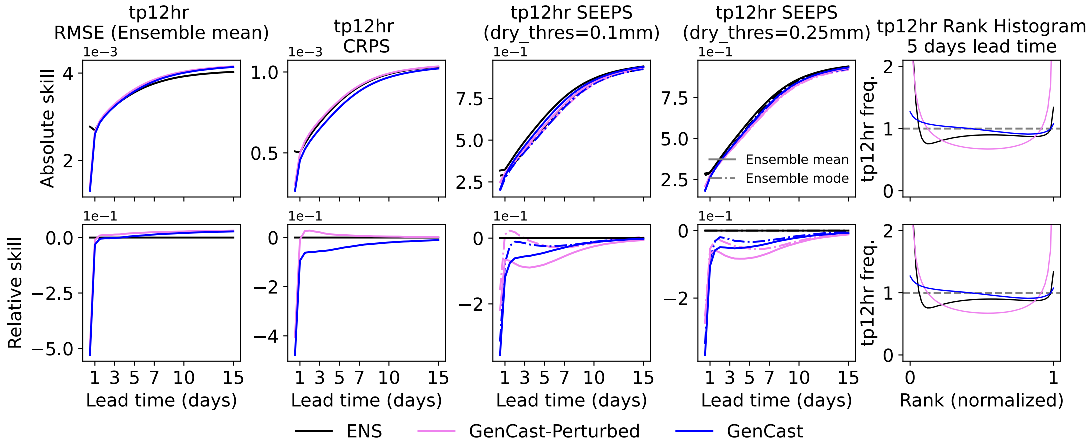

Figure B4: **Preliminary results on 12h accumulated precipitation show GenCast outperforming ENS.** Results analogous to Figure B3 on 12 hour precipitation.

B.3 Spectrum for additional variables

We provide additional spectral results to supplement those the main paper, showing 10 representative variables: z500, z850, q700, q925, t850, t300, u850, 2t, 10u and msl (Figure B5, Figure B6).

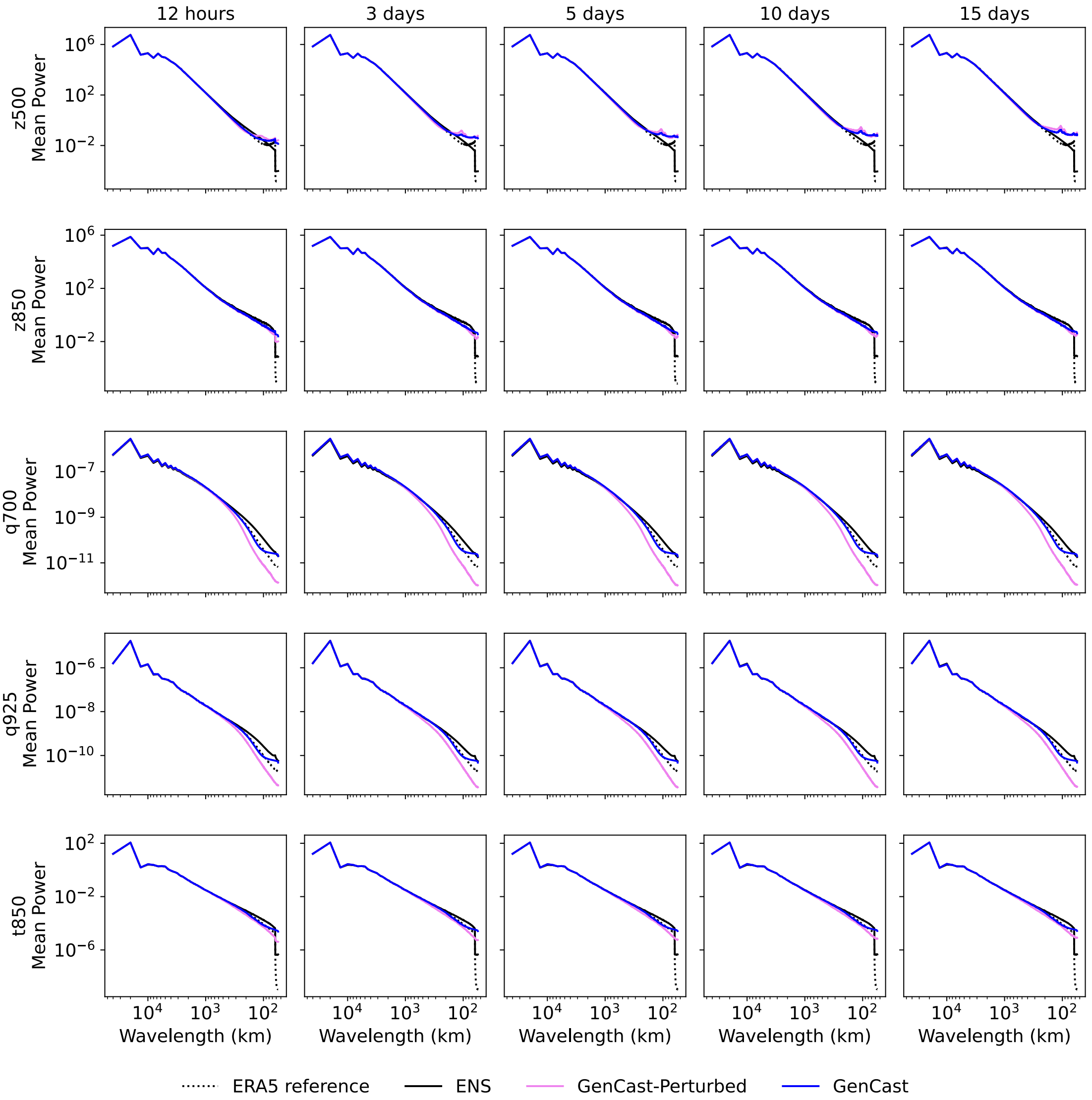

Figure B5: **GenCast's power spectra closely match the ERA5 ground truth.** Power spectrum plots for z500, z850, q700, q925 and t850.

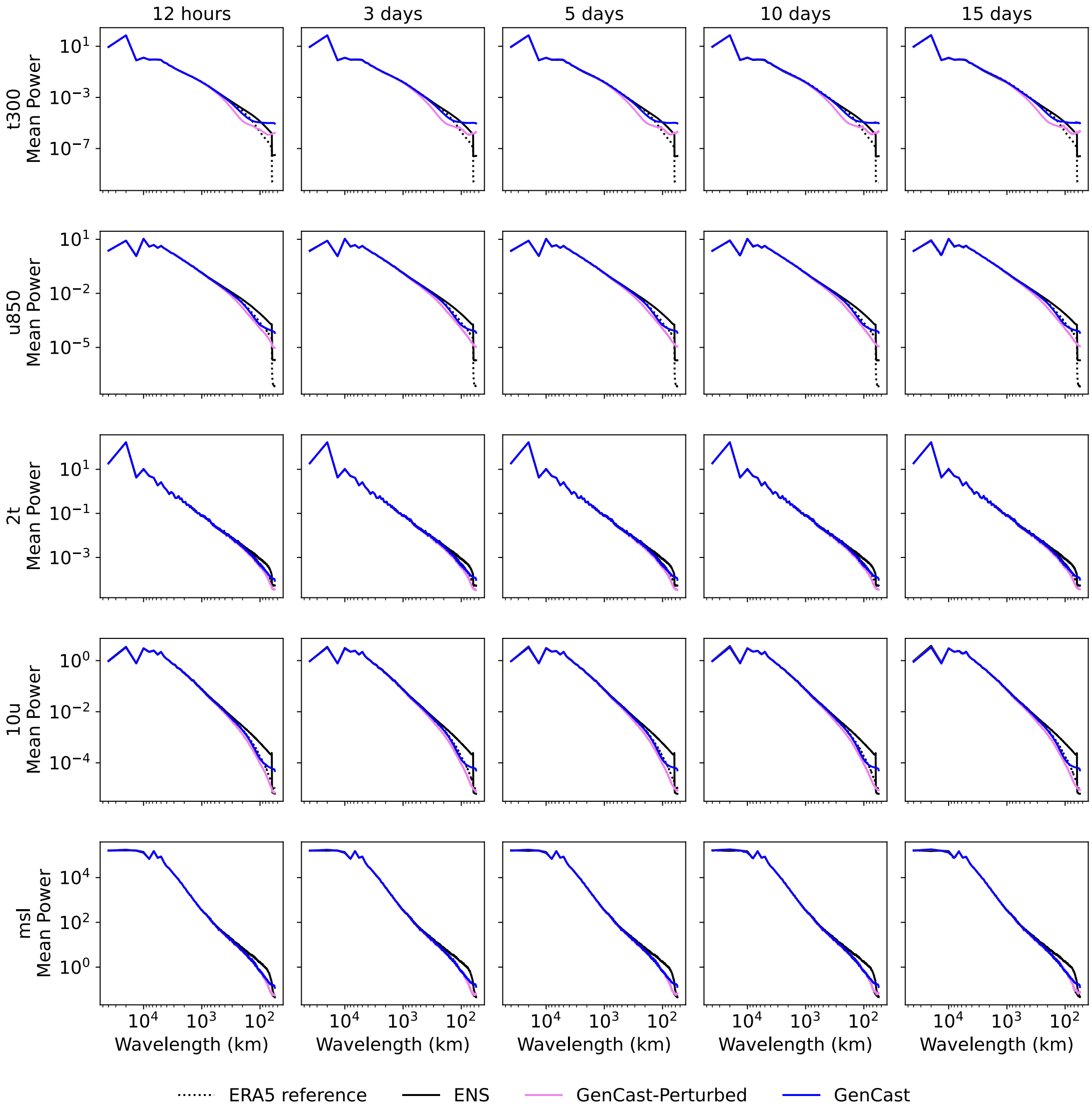

Figure B6: **GenCast's power spectra closely match the ERA5 ground truth.** Power spectrum plots for t300, u850, 2t, 10u and msl.

B.4 GenCast-Perturbed scorecards

Figure B7 shows RMSE and CRPS scorecards comparing GenCast-Perturbed to ENS. GenCast-Perturbed achieves strong results, with better or competitive CRPS compared to ENS on 82% of scorecard targets. When compared to GenCast, it shows consistently worse CRPS, and more comparable but slightly worse ensemble mean RMSE (Figure B8).

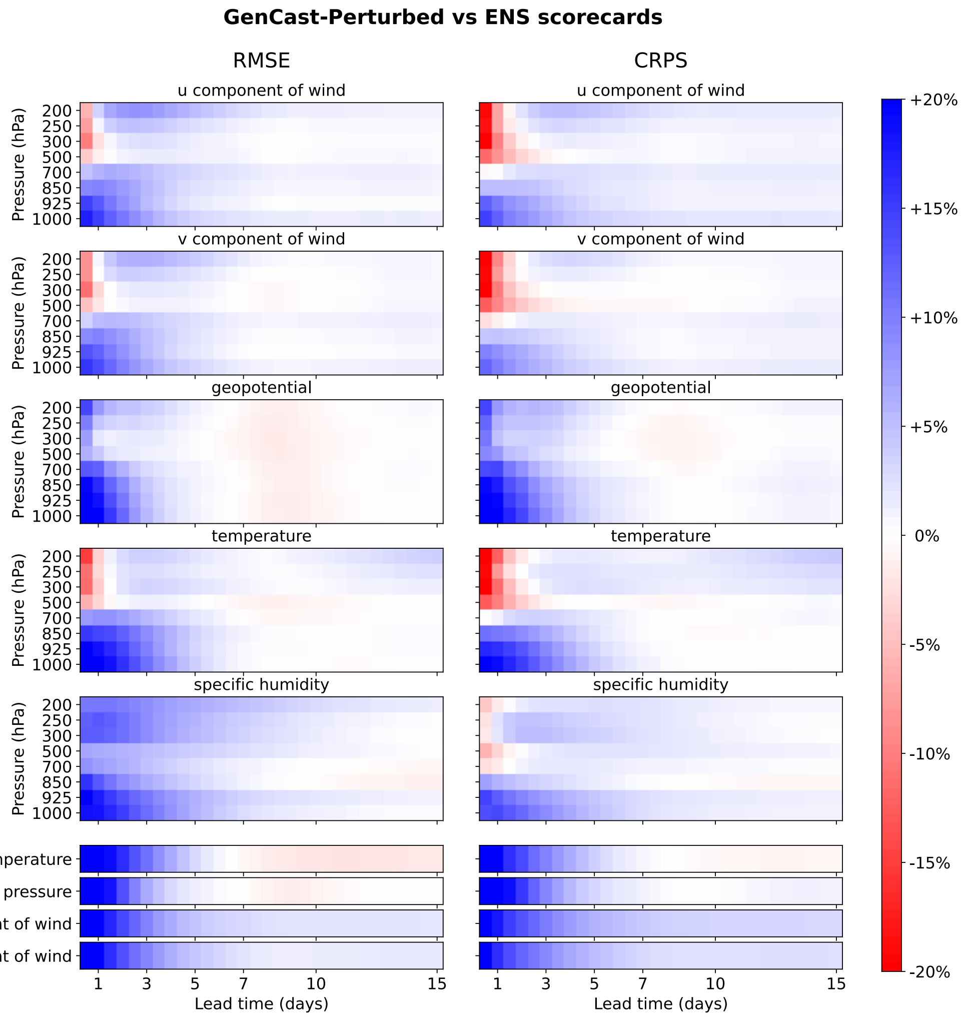

Figure B7: **GenCast-Perturbed is competitive with ENS on CRPS.** RMSE and CRPS scorecard comparing GenCast-Perturbed to ENS (4), dark blue (resp. red) means GenCast-Perturbed is 20% better (resp. worse) than ENS, and white means they perform equally. Analogous to Figure 3 and Extended Data Figure 1.

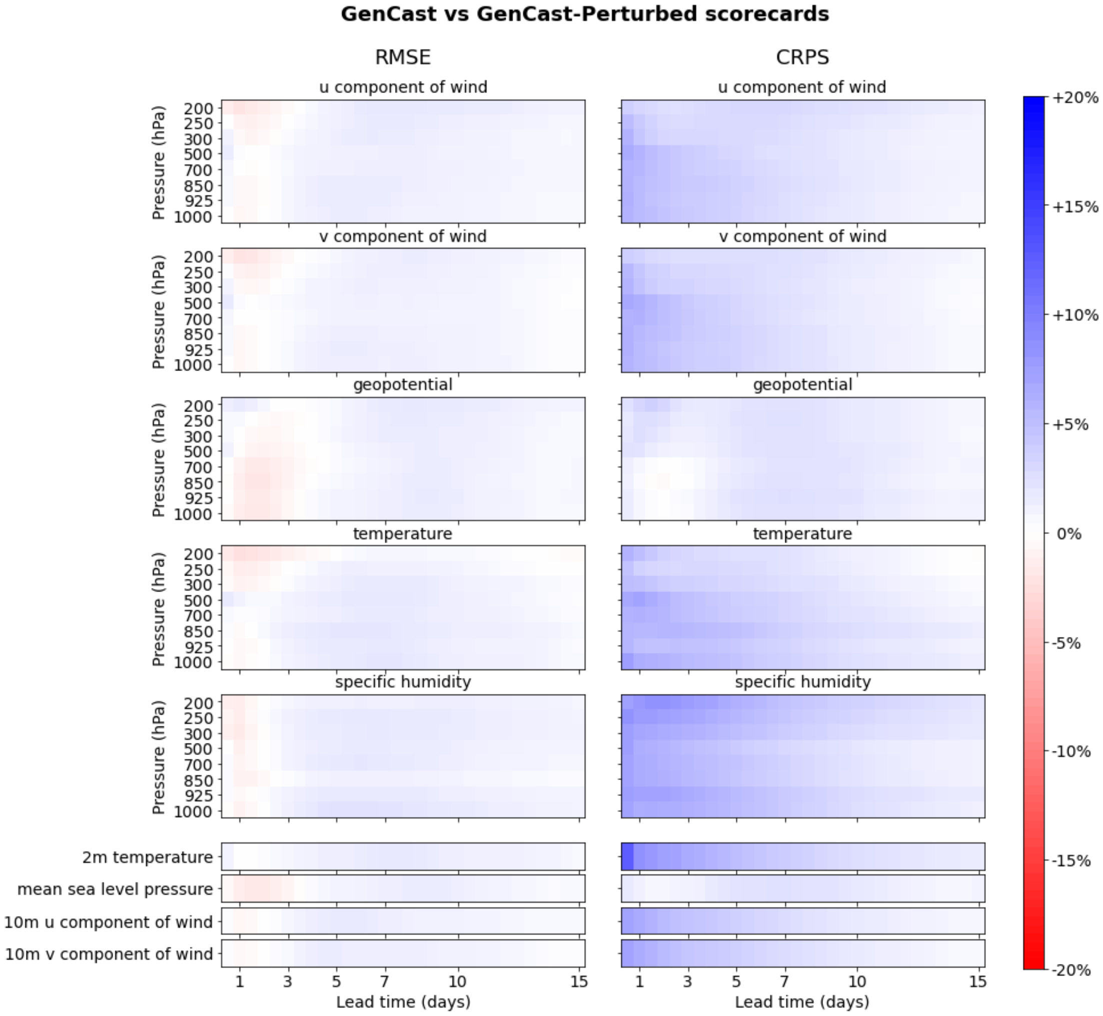

Figure B8: **GenCast outperforms GenCast-Perturbed on CRPS.** RMSE and CRPS scorecard comparing GenCast to GenCast-Perturbed. Analogous to Figure 3, Extended Data Figure 1 and B7.

B.5 Tropical cyclones

This section provides supplementary results for the tropical cyclone evaluation, including statistical significance.

B.5.1 Deterministic evaluation

The pairing process of the cyclone position error analysis ('Cyclone position error evaluation' in Methods) means that overprediction and underprediction are not penalised in this evaluation (unlike with strike probability). However, we computed true-positive rates for GenCast and ENS and confirmed that they were similar across lead times.

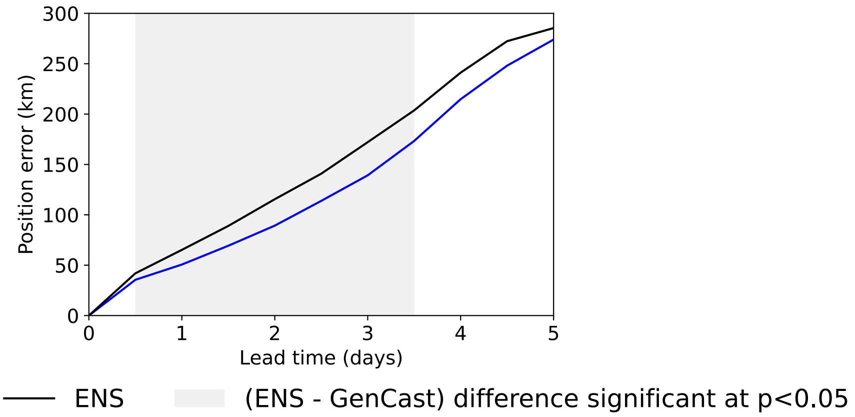

Figure B9: **GenCast achieves lower position error than ENS when tracking tropical cyclones.** Mean position error of ensemble mean cyclone tracks. Statistical significance ($p < 0.05$) of the improvement yielded by GenCast is indicated by light grey shading (between 0.5 and 3.5 days inclusive).

As shown in figure Figure B9, GenCast's position errors are significantly ($p < 0.05$) lower than ENS between 12 hours and 3.5 days, inclusive.

B.5.2 Probabilistic evaluation

When tracks from GenCast and ENS are compared to cyclone tracks from their own ground truths (ERA5 and HRES-fc0, respectively), Figure B11 shows that GenCast significantly outperforms ENS across a broad range of cost-loss ratios at a lead time of 1 day, as well as at smaller cost-loss ratios at longer lead times up to 7 days.

We note that due to differences between ERA5 and HRES-fc0, running the TempestExtremes cyclone tracker on each dataset produces different cyclone locations and counts. On average across 2019, ERA5 has 1.51 cyclones per timestep and HRES-fc0 has 1.85 cyclones per timestep, which gives HRES-fc0 a base rate that is 23% greater than ERA5. Figure B10 plots the total cyclone counts tracked in the ground truth dataset at each time of the year, for both ERA5 and HRES-fc0 (Figure B10). However, REV accounts for this difference in the base rates by virtue of its normalisation with respect to climatology and the perfect forecast, and is thus a fair metric to use when comparing methods evaluated against different ground truths. Furthermore, GenCast still outperforms ENS beyond 1 day lead times even when using HRES-fc0 ground truth, which puts GenCast at a disadvantage (Figure B12).

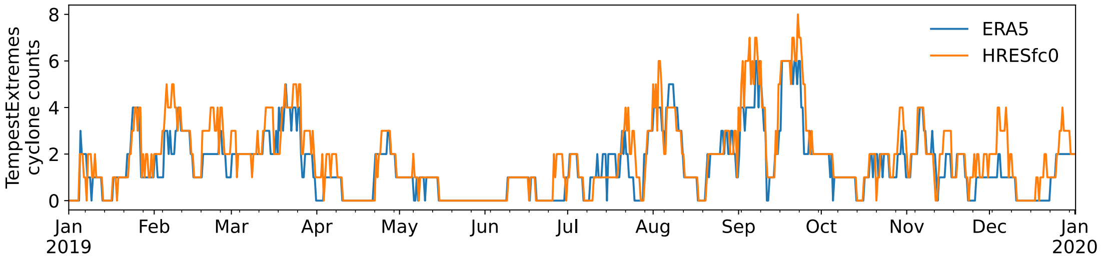

Figure B10: **Per-timestep TempestExtremes cyclone counts for ERA5 and HRES-fc0.** The two time-series exhibit high correlation, but HRES-fc0 has 23% more cyclones than ERA5. The TempestExtremes tracker is applied to these analysis datasets. We then arbitrarily picked a lead time of 4 days to extract cyclone count (all lead times 0-10 days yield very similar results).

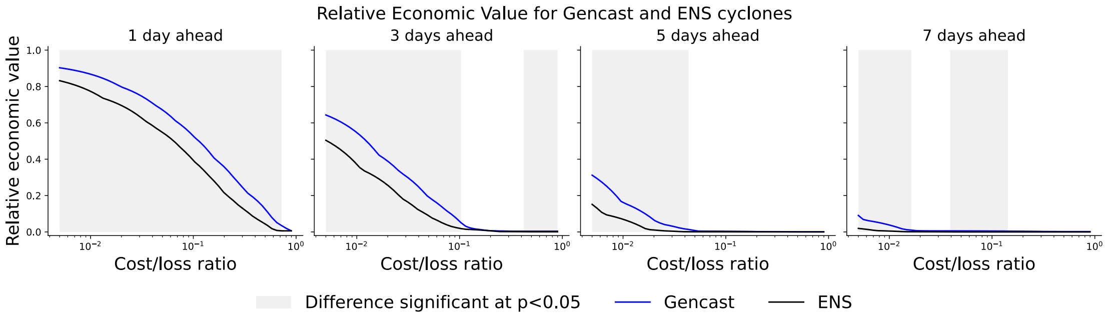

Figure B11: **GenCast achieves better track probability REV than ENS when forecasting tropical cyclones.** Relative economic value for cyclone track prediction when comparing GenCast and ENS (4) to ERA5 (27) and HRES-fc0 targets, respectively. Statistical significance ($p < 0.05$) of the improvement yielded by GenCast is indicated by light grey shading. The surprising statistical confidence at high cost/loss ratios at a 3 and 7 day lead times is due to GenCast having small but non-zero skill and ENS having exactly zero skill.

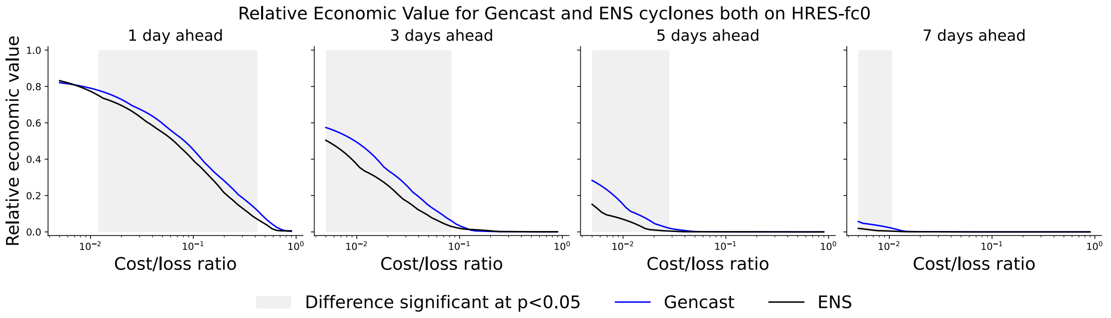

Figure B12: **GenCast's cyclone forecasts still achieve better track probability REV than ENS when compared against HRES-fc0.** Relative Economic Value for cyclone track prediction when comparing both GenCast and ENS (4) to HRES-fc0 targets. Statistical significance ($p < 0.05$) of the improvement yielded by GenCast is indicated by light grey shading. The gap between ENS and GenCast is smaller for 1 day ahead because our tracking procedure concatenates a HRES-fc0 context window to GenCast's predictions ('Cyclone tracker' in Methods), which results in a distribution shift that disadvantages GenCast. However, in general, GenCast is still statistically significantly better than ENS despite the disadvantage of comparing against a different ground truth.

B.6 Pooled evaluation

Extended Data Figures 6 and 7 show averaged-pooled and max-pooled CRPS scorecards for GenCast relative to ENS. Figure B15 and Figure B16 show averaged-pooled and max-pooled CRPS scorecards for GenCast-Perturbed relative to ENS. We aggregate the u-component and v-component of wind into wind speed, and include tp12hr in our surface variables. This results in 5400 pooled verification targets across all variables, lead times, and spatial scales. Aggregating over all pooled verification targets, GenCast outperforms ENS's average-pooled CRPS in 98.1% of cases (compared with 86% for GenCast-Perturbed). For max-pooled CRPS, GenCast outperforms ENS in 97.6% of cases (compared with 50% for GenCast-Perturbed).

These results hold up in our surface-only 0.25° pooled evaluation, where GenCast outperforms both ENS and GenCast-Perturbed's CRPS, often with relative performance improving as pooling size increases (Figure B13 and Figure B14).

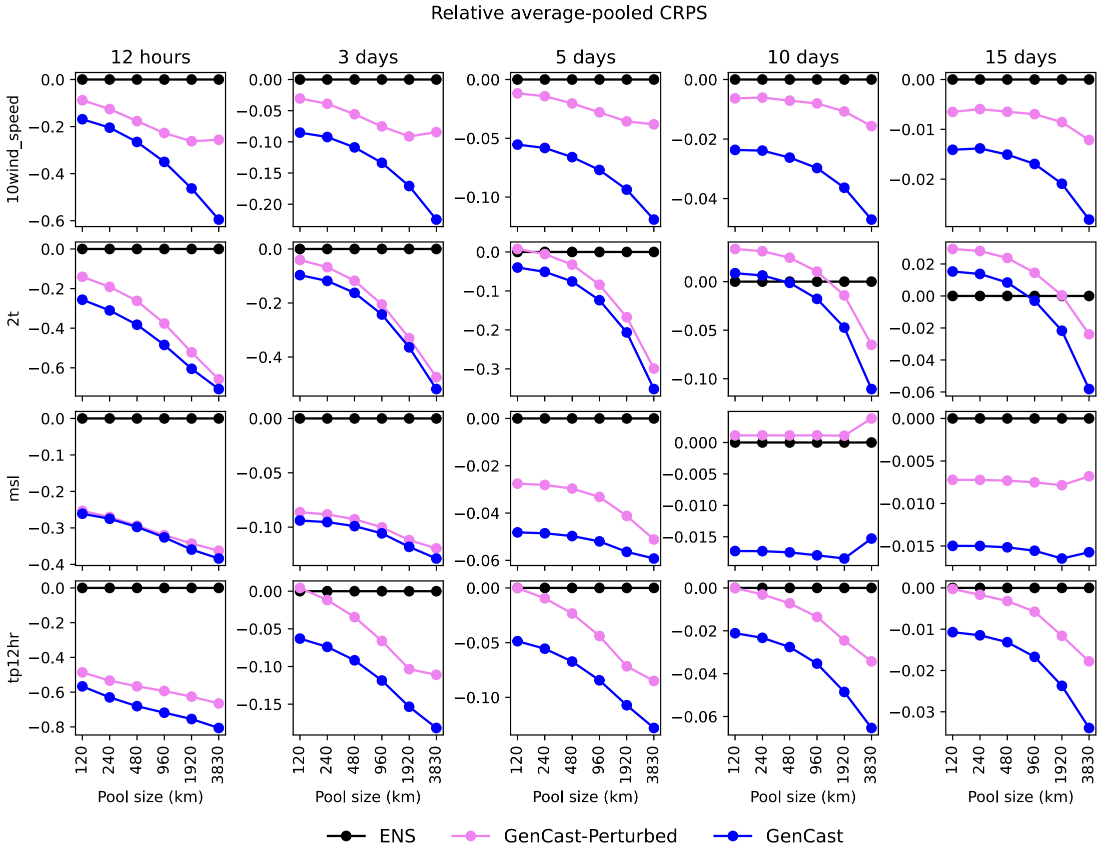

Figure B13: **GenCast ensembles capture spatial dependency structure (Average-pooled CRPS).** Average-pooled CRPS skill score plots for 0.25 deg surface variables.

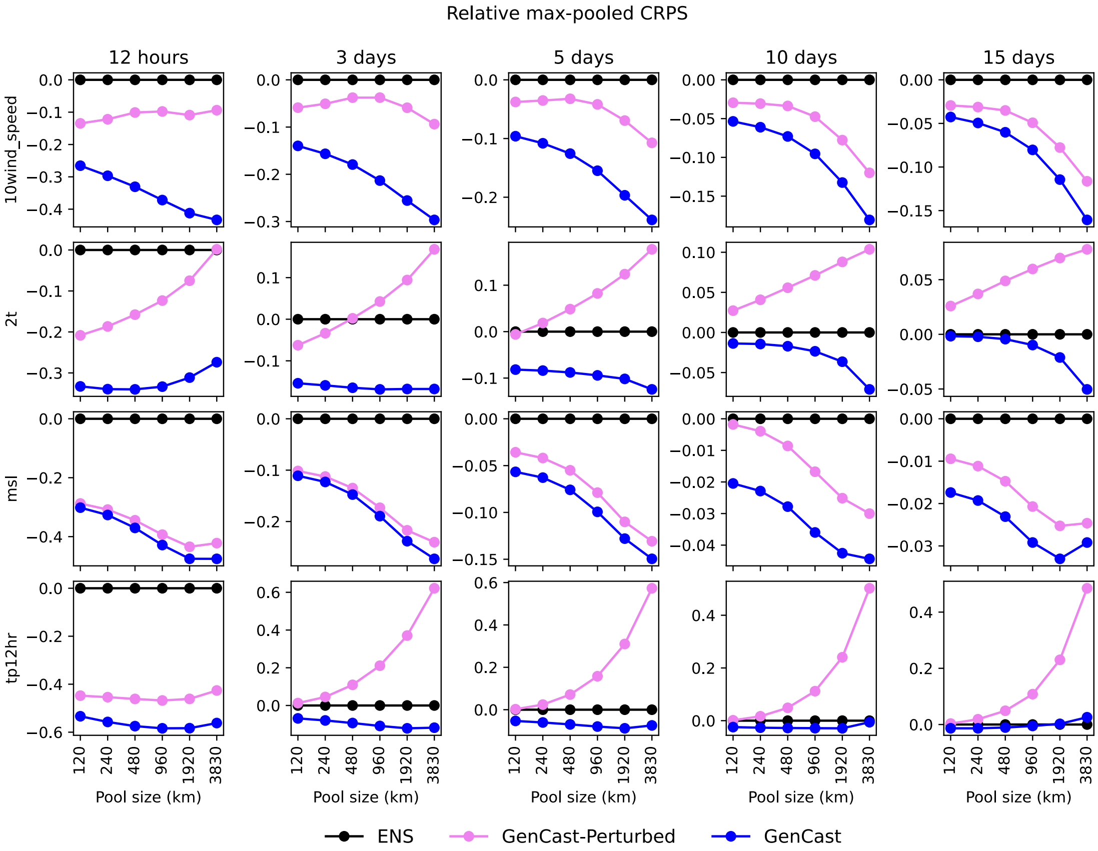

Figure B14: **GenCast ensembles capture spatial dependency structure (Max-pooled CRPS).** Max-pooled CRPS skill score plots for 0.25 deg surface variables.

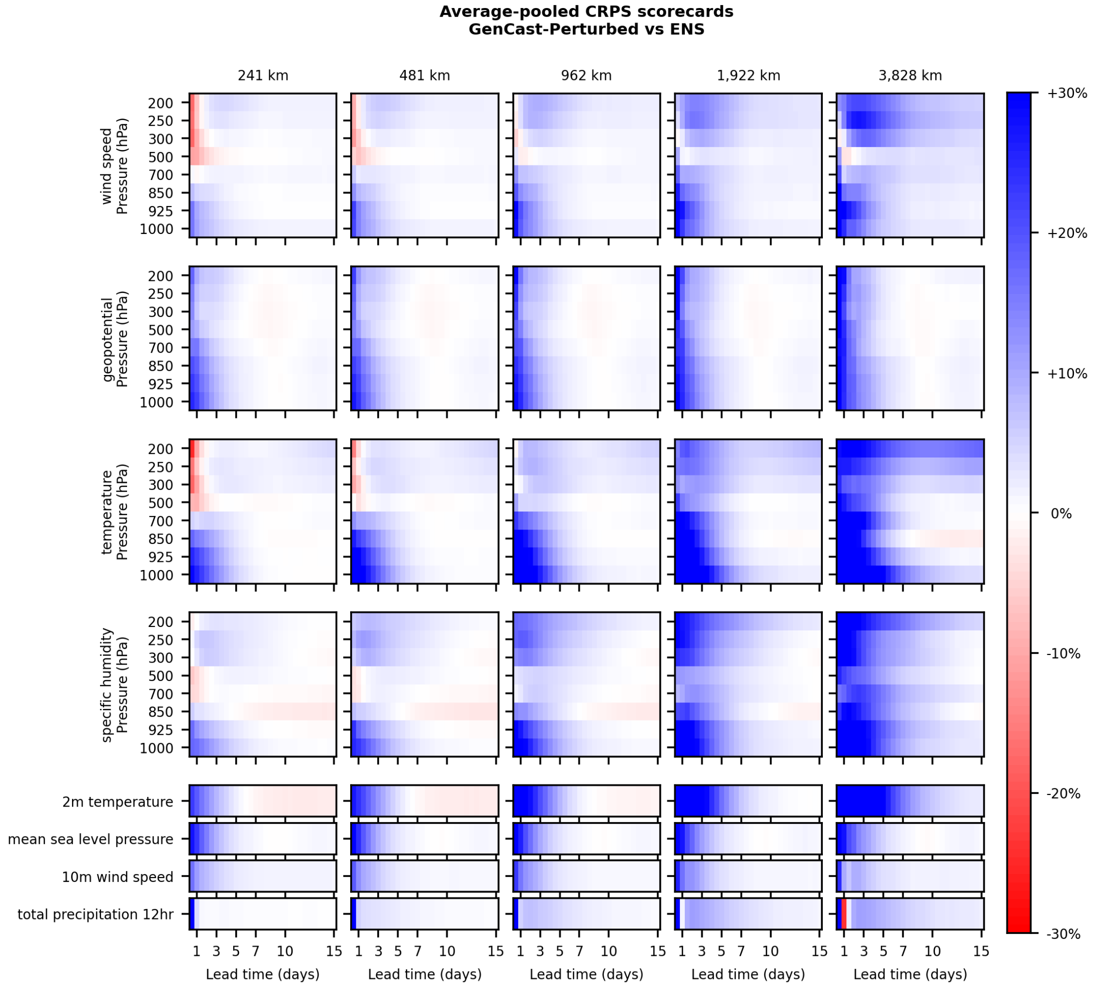

Figure B15: **GenCast-Perturbed often outperforms ENS on Average-pooled CRPS.** Average-pooled CRPS scorecard comparing GenCast-Perturbed and ENS at varying spatial scales, dark blue (resp. red) means GenCast-Perturbed is 30% better (resp. worse) than ENS, and white means they perform equally.

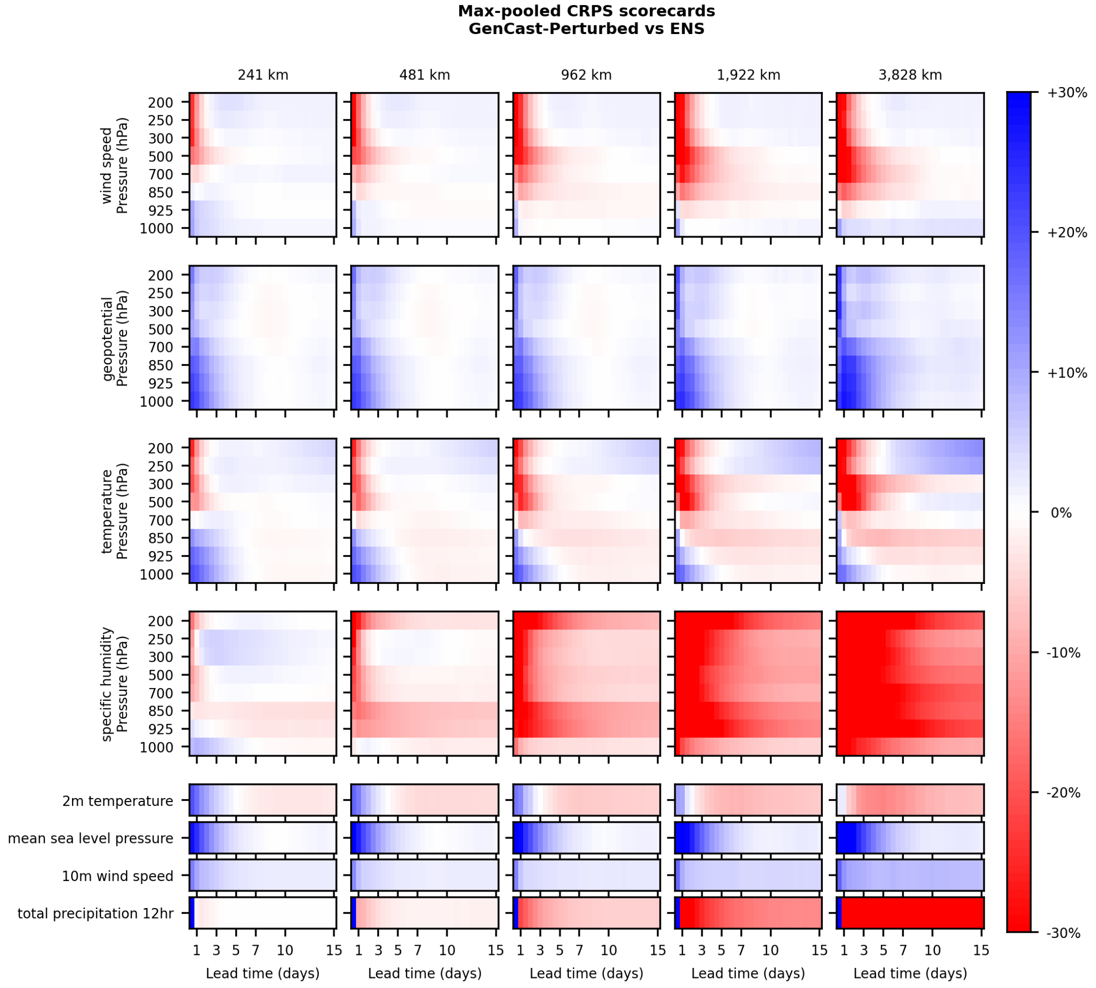

Figure B16: **GenCast-Perturbed performs worse on Max-pooled CRPS than Average-pooled CRPS, relative to ENS.** Max-pooled CRPS scorecard comparing GenCast-Perturbed and ENS at varying spatial scales, dark blue (resp. red) means GenCast-Perturbed is 30% better (resp. worse) than ENS, and white means they perform equally.

B.7 Regional wind power forecasting statistical significance

Figure B17 shows the relative CRPS of GenCast and ENS on the regional wind power forecasting task described in the Main. Lead times at which the difference in CRPS is statistically significant ($p < 0.05$) are shaded grey. Differences are significant up to a 7 day lead time for all pool sizes, and selected lead times beyond that. Figure B18 illustrates the relative performance of GenCast-Perturbed, outperforming ENS but performing worse than GenCast.

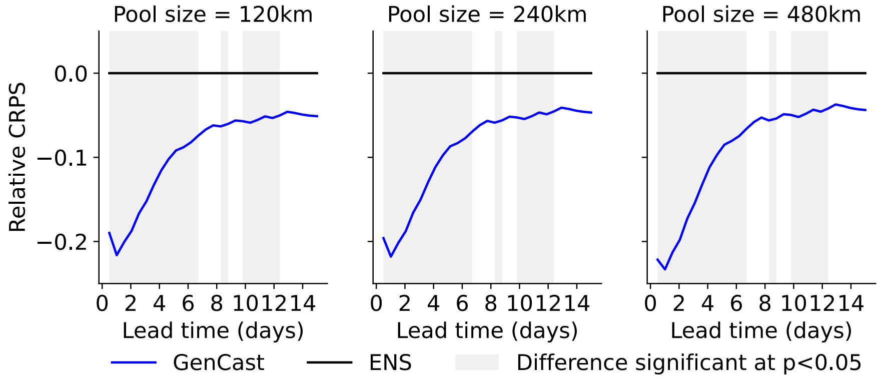

Figure B17: **GenCast outperforms ENS on regional wind power forecasting.** Grey shading indicates the lead times at which the improvement achieved by GenCast is statistically significant ($p < 0.05$).

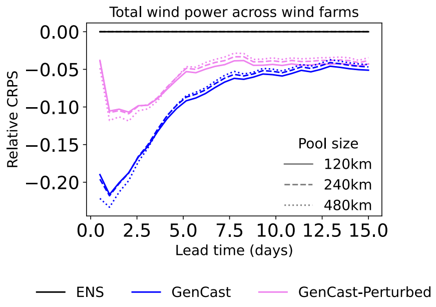

Figure B18: **Regional wind power results including GenCast-Perturbed.**

B.8 Empirical support of initialisation time and evaluation time choices for verification

B.8.1 Lead time interpolation in regional wind farm evaluation

The 'ENS initialization and evaluation times' section in Methods motivates and describes the lead time interpolation method for evaluating ENS and GenCast on the same set of validity times for regional wind power forecasting. We were able to download 06/18 UTC initialised ENS forecasts for some surface variables for 2018 to validate and justify this approach. Figure B19 shows that on the regional wind power forecasting task, lead time interpolation with 00/12-initialised ENS forecasts actually overestimates the performance of 06/18-initialised ENS forecasts on 06/18 UTC targets. This is particularly the case at a 12-hour lead time, where 06/18-initialised ENS performs 6% worse than 00/12-initialised ENS with lead time interpolation. This suggests that the lead time interpolation is in fact advantaging ENS in the regional wind power forecasting experiment, and explains the non-monotonicity in GenCast's relative performance at the shortest lead times in Figure 4a.

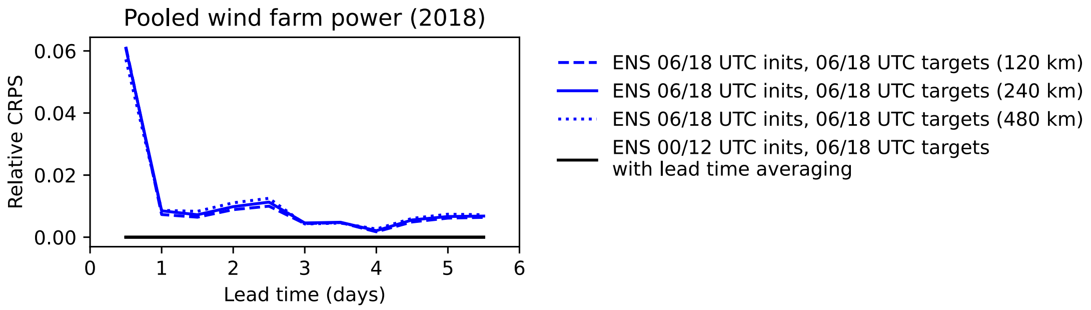

Figure B19: **Lead time interpolation (as used for the regional wind power experiment) advantages ENS.** Relative CRPS of ENS (4) (06/18 UTC initialisations) compared to ENS 00/12 UTC initialisations with the lead time averaging applied to 06/18 UTC targets, on the regional wind power forecasting task.

B.8.2 Initialisations used for GenCast evaluation

Our main results always use 06/18 UTC initialisation times when evaluating GenCast. Figure B20 shows a scorecard comparing GenCast initialised at 00/12 and 06/18 UTC, showing how the 00/12-initialised forecasts have a systematic advantage due to the ERA5 look-ahead at those initialisation times. This motivates the use of 06/18 UTC initialisations for the evaluations of GenCast as the most conservative approach.

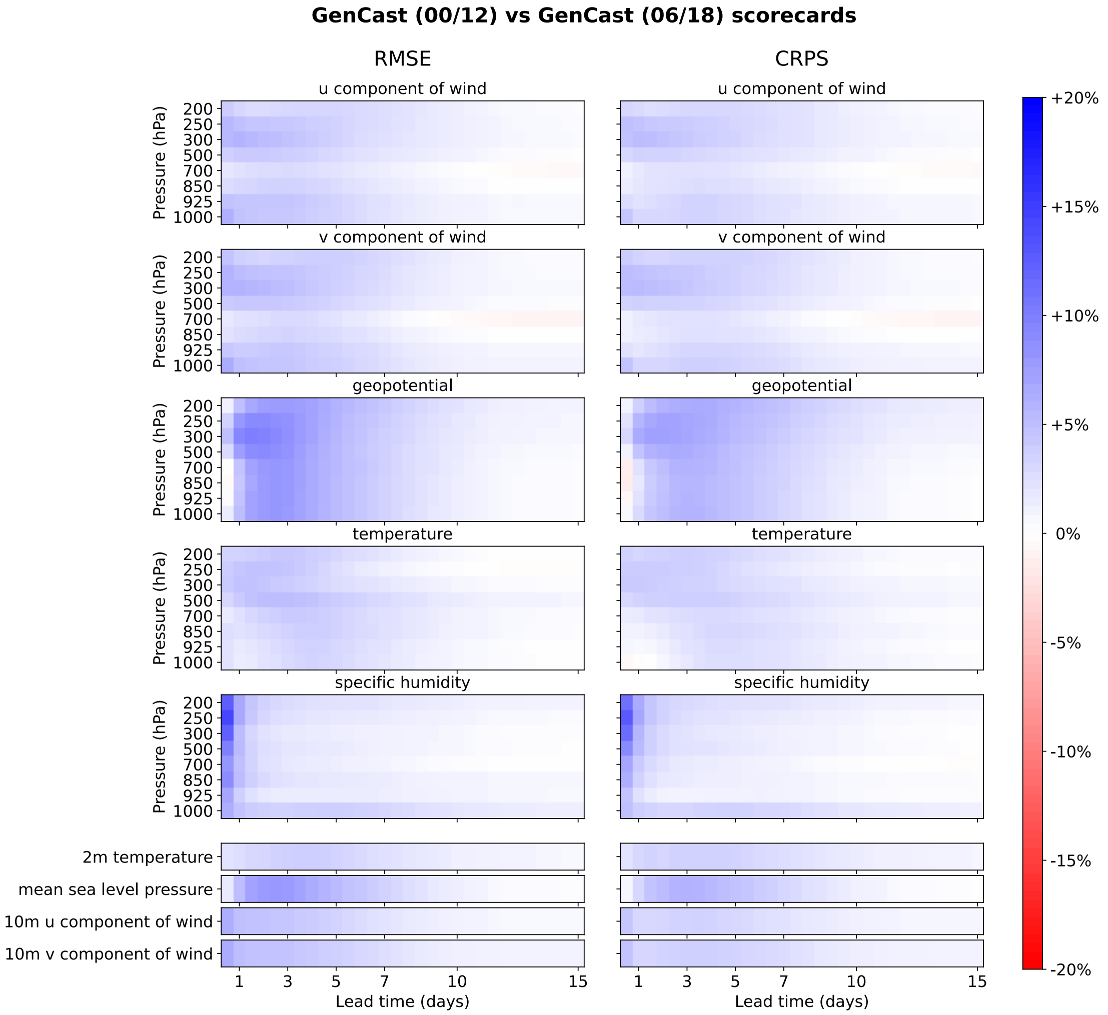

Figure B20: **00/12-initialised GenCast forecasts enjoy a systematic advantage.** Scorecard comparing the performance of GenCast evaluated on 00/12 UTC initialisation times, to GenCast evaluated on the 06/18 UTC initialisation times (the latter is used in the rest of the evaluation in the paper). The 9 hour assimilation look-ahead of the 00/12 UTC initialisations gives the model a consistent advantage in CRPS and Ensemble-Mean RMSE over the 3 hour assimilation look-ahead of the 06/18 UTC initialisations. This highlights the importance of accounting for assimilation window look-ahead when comparing metrics across model evaluations.

Appendix C Forecast visualisations

C.1 Tropical cyclone tracks

Here we provide further cyclone track visualisations. We picked some of the most extreme cyclones in 2019, based on several measures: the deadliest, the costliest, the strongest globally, and the strongest in the Atlantic basin. The deadliest cyclone was Cyclone Idai, which killed over 1500 people and caused a humanitarian crisis in Mozambique, Zimbabwe, and Malawi (Figure C1). The costliest cyclone was Typhoon Hagibis, with struck Japan and caused damages of 17.3 billion USD in 2019 (Figure C2). The strongest cyclone was Typhoon Halong, with a minimum barometric pressure of 905 hPa and maximum 1-minute sustained winds of 305 km/h (85 m/s) (Figure C3). The strongest cyclone in the Atlantic basin was Hurricane Dorian, which caused catastrophic damage in the Bahamas as a Category-5 hurricane before moving northwards along the coast of the United States, where it caused further damage and power outages (Figure C4).

In our visualisations, we set the validity dates as the day of landfall for Idai and Hagibis. For Halong, which did not make landfall, we set the validity date as the day the cyclone reached Category-5 status. For Dorian, we picked a time when the cyclone had weakened to Category-2 status, to also capture capture its effects on the East coast of the US.

Due to the 6-hour offset in initialisation and validity times between GenCast and ENS in our cyclone analysis (06/18 UTC and 00/12 UTC, respectively), in our visualisations we show ENS forecasts initialised both 6 hours before and 6 hours after GenCast's (with the same lead time). A 12-hour later initialisation time can give a model information on a cyclone that is 12 hours further into its development, which can have a material effect on cyclone forecasts in some cases. For example, when initialised 12 hours later, ENS performs substantially better at predicting Hurricane Dorian's curve northward along the eastern coast of the United States after passing the Bahamas (Figure C4a vs Figure C4k).

These visualisations are for illustration purposes only and have not been chosen as representative of differences between GenCast and ENS. We refer the reader to our systematic ensemble mean track position error and REV evaluation for a rigorous and principled comparison of model performance (Figure 4b, Figure B11, 'Tropical cyclone evaluation' in Methods). We note that in some cases the trajectory of the main cyclone being visualised coincides with the trajectory of another cyclone nearby (e.g. Figure C2a, Figure C4a). Finally, whereas tracks with short lead times branch out from an initial point, some tracks for long lead times *start* from different places (e.g. Figure C3a,f,k). This is because the forecasts are initialised before the cyclone started and the model is uncertain about whether cyclogenesis will occur and, if so, where it will occur.

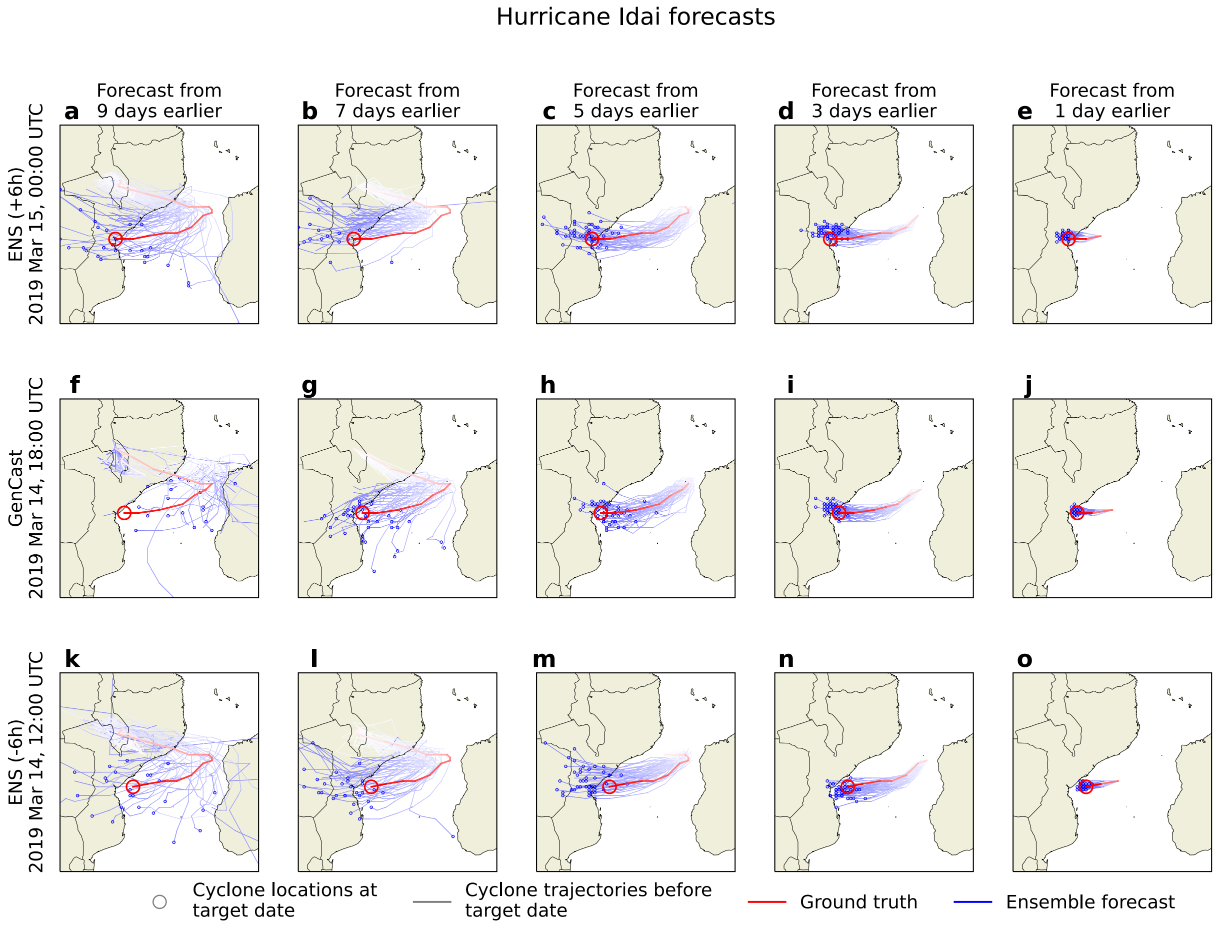

Figure C1: Visualisation of Cyclone Idai trajectory forecasts.

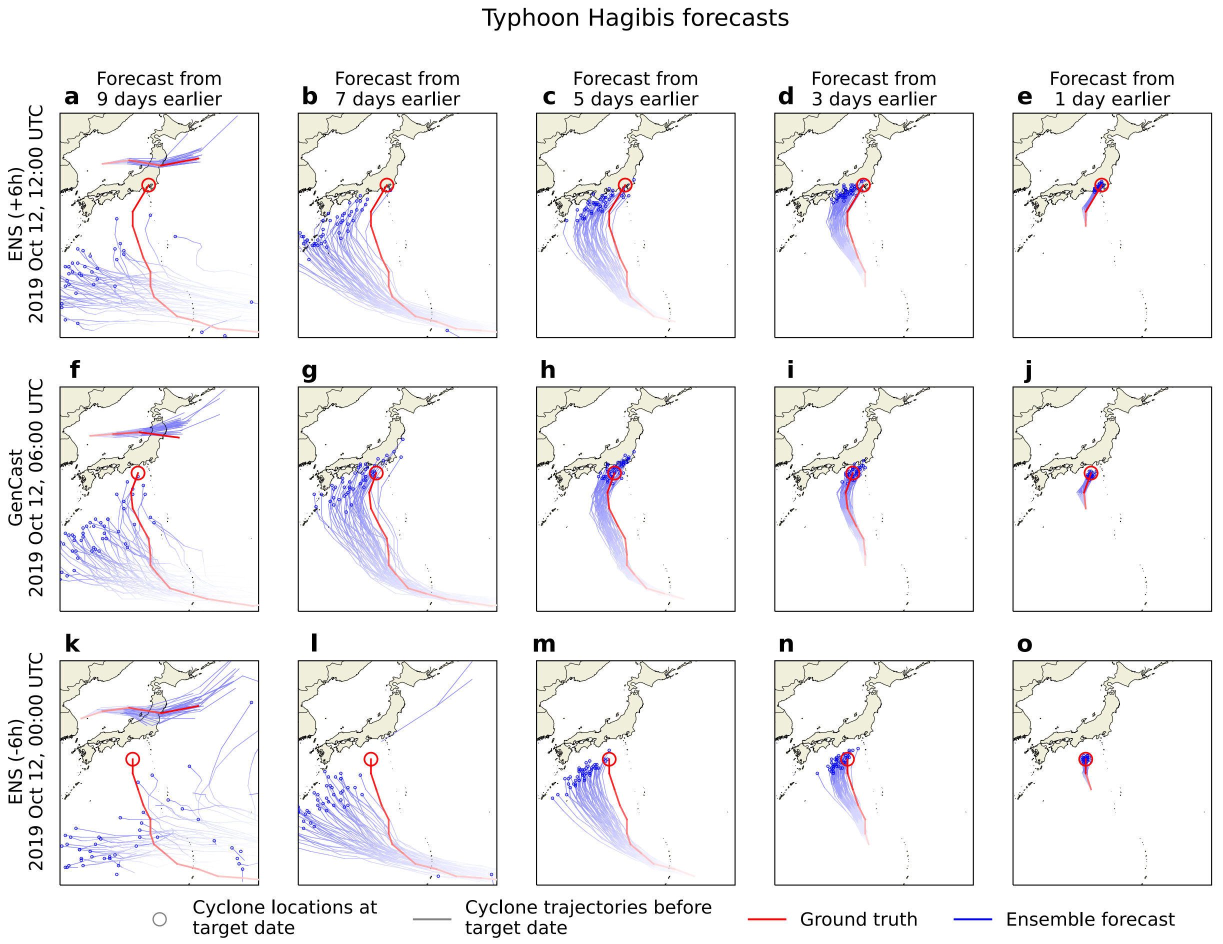

Figure C2: Visualisation of Typhoon Hagibis trajectory forecasts.

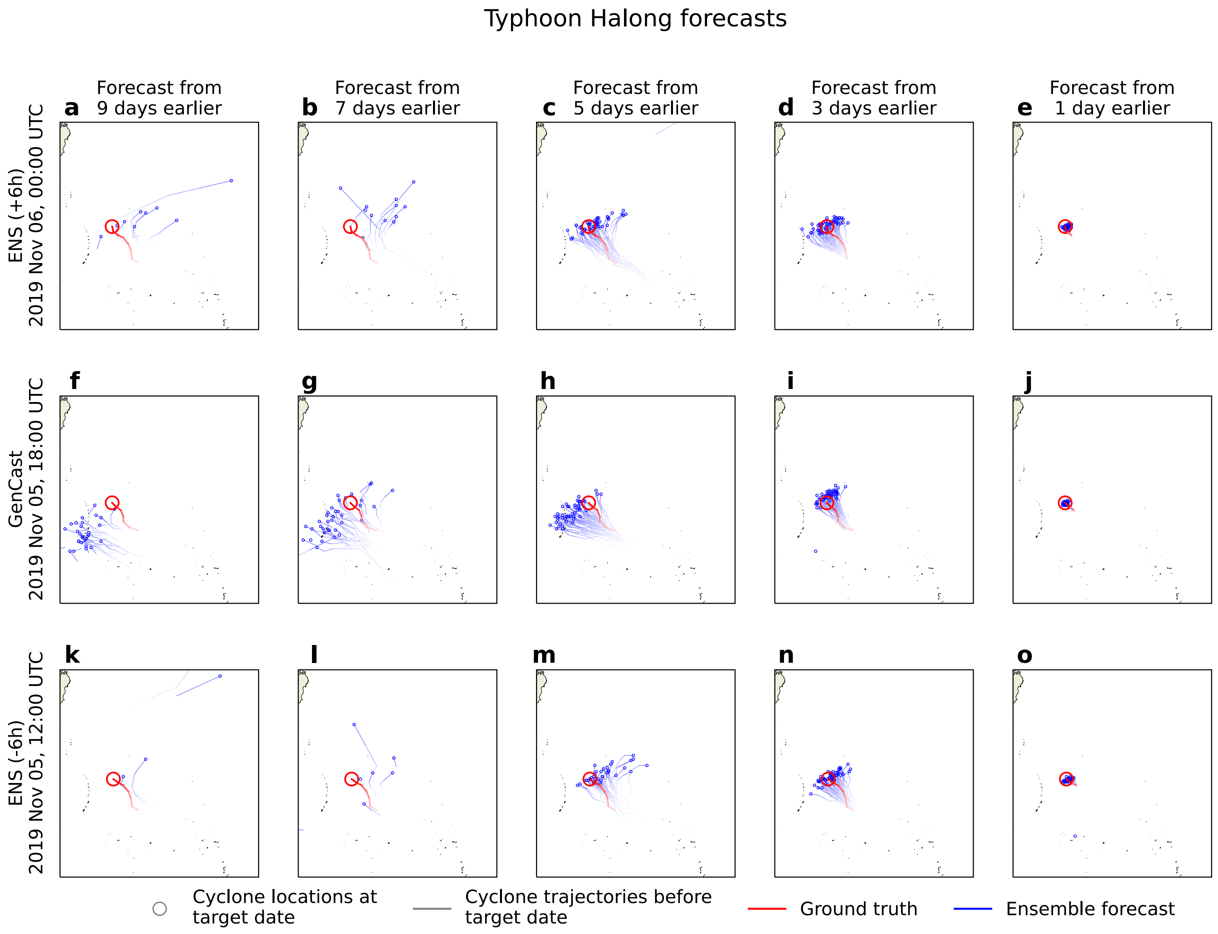

Figure C3: Visualisation of Typhoon Halong trajectory forecasts.

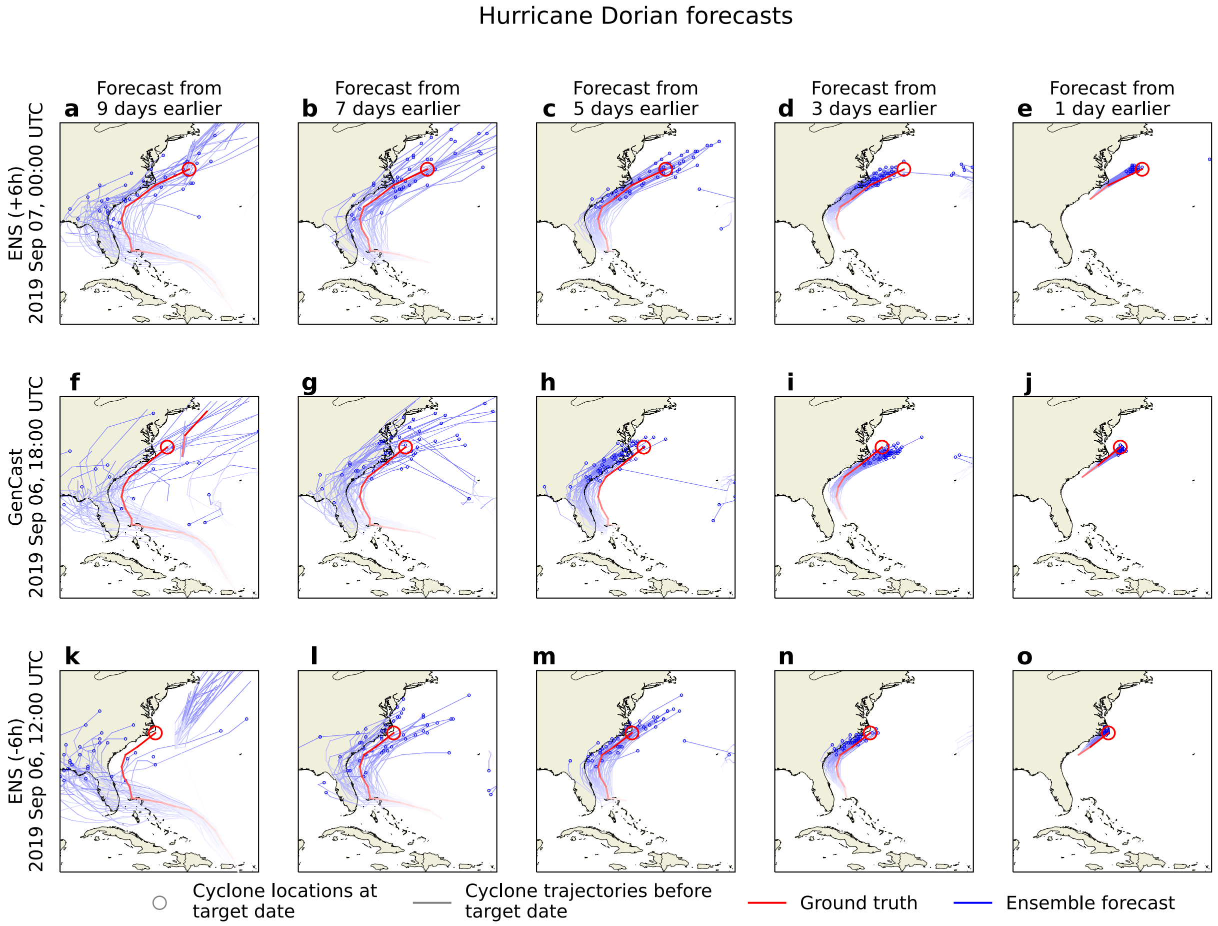

Figure C4: Visualisation of Hurricane Dorian trajectory forecasts.

C.2 Global forecasts

We provide representative global visualisations for various lead times, and 10 representative variables including specific humidity at 700 hPa (Figure C5), specific humidity at 925 hPa (Figure C6), geopotential at 500 hPa (Figure C7), geopotential at 850 hPa (Figure C8), temperature at 850 hPa (Figure C9), temperature at 300 hPa (Figure C10), u component of wind at 850 hPa (Figure C11), 2 meter temperature (Figure C12), 10 meter u component of wind (Figure C13), and mean sea level pressure (Figure C14). For each variable-lead time combination we choose the 2019 initialisation time with median CRPS error. This means that each figure shows a snapshot from a different forecast trajectory at each lead time. In each case we plot the first ensemble member.

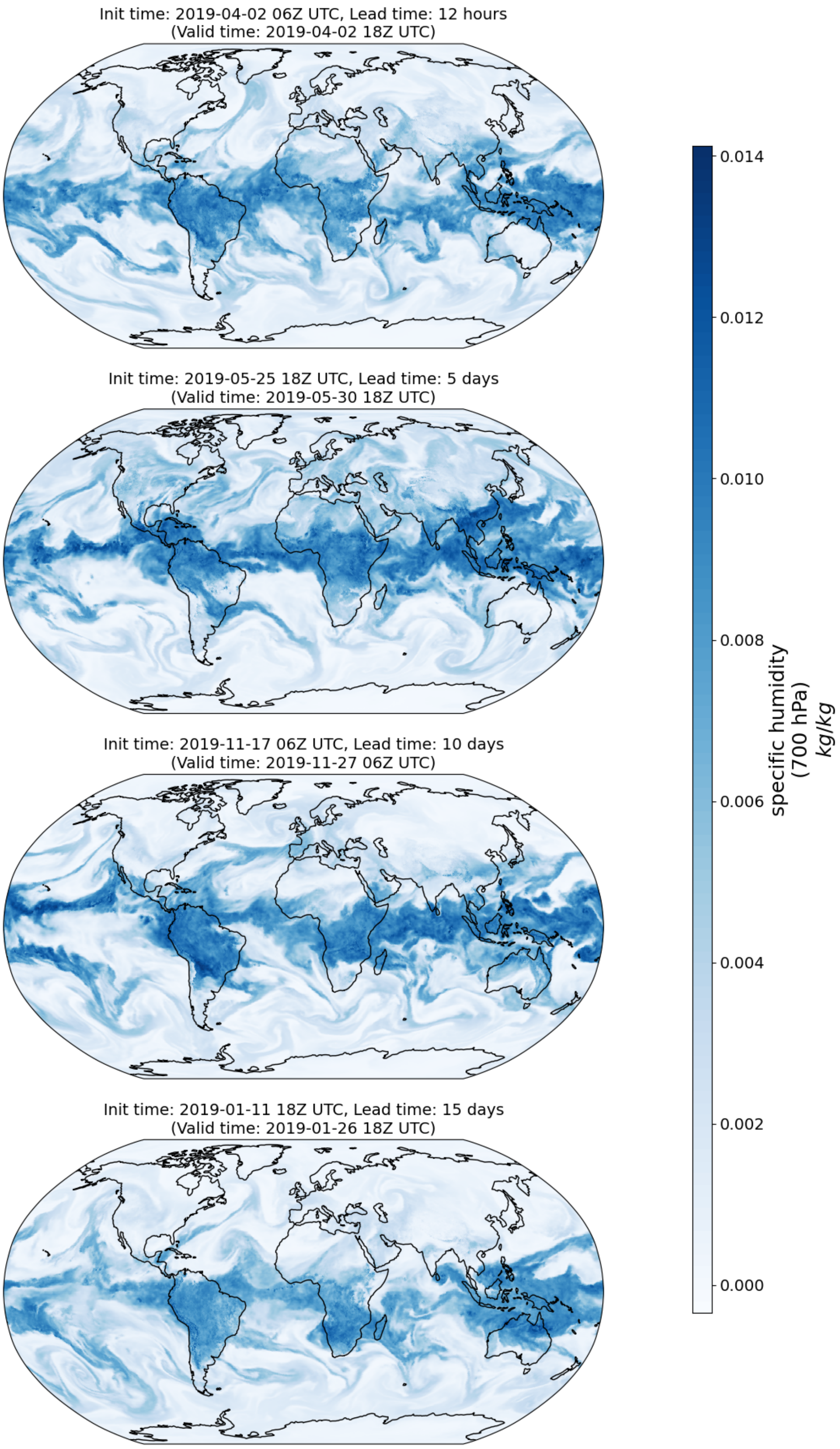

Figure C5: Visualisation of specific humidity at 700 hPa.

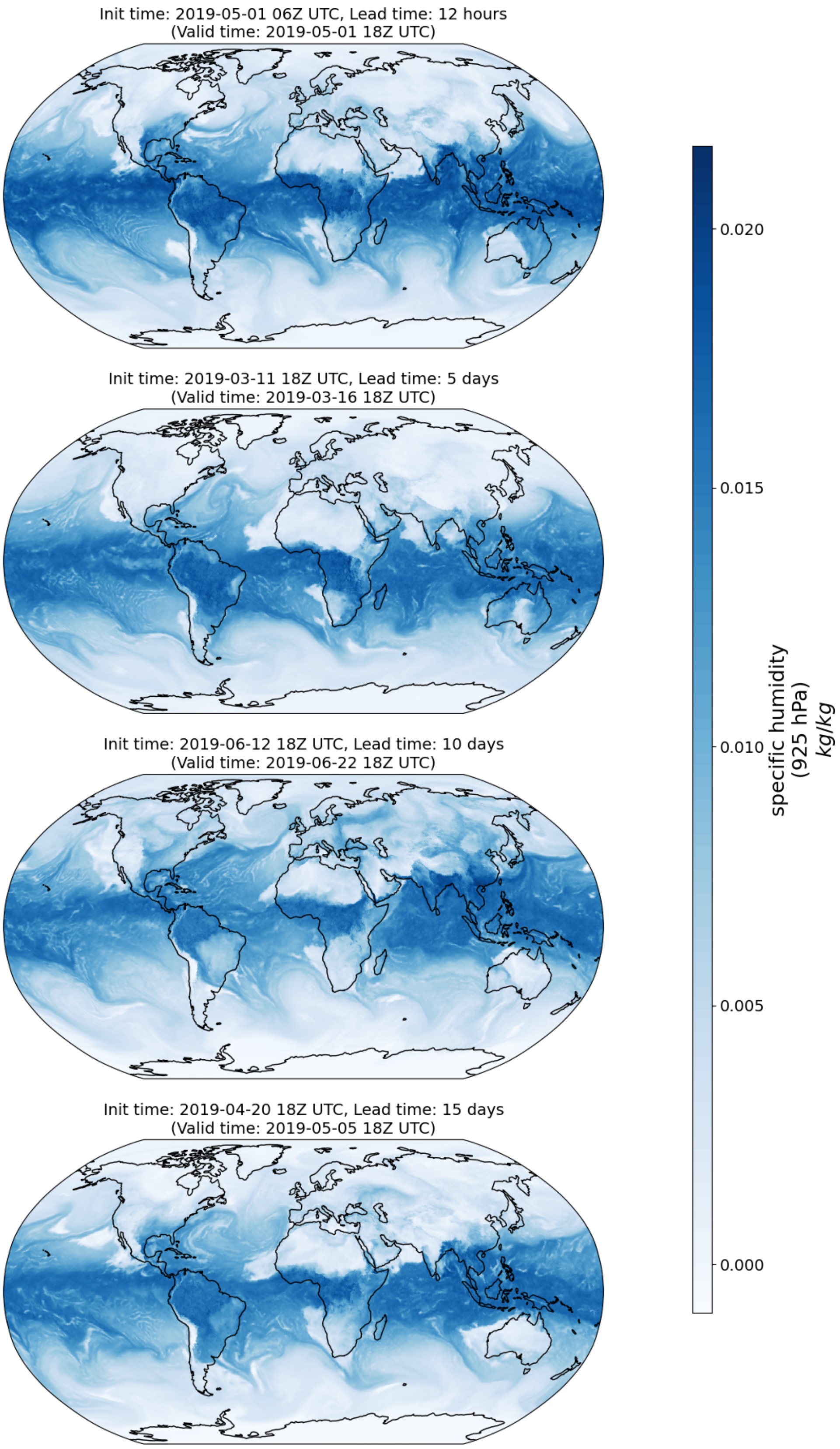

Figure C6: Visualisation of specific humidity at 925 hPa.

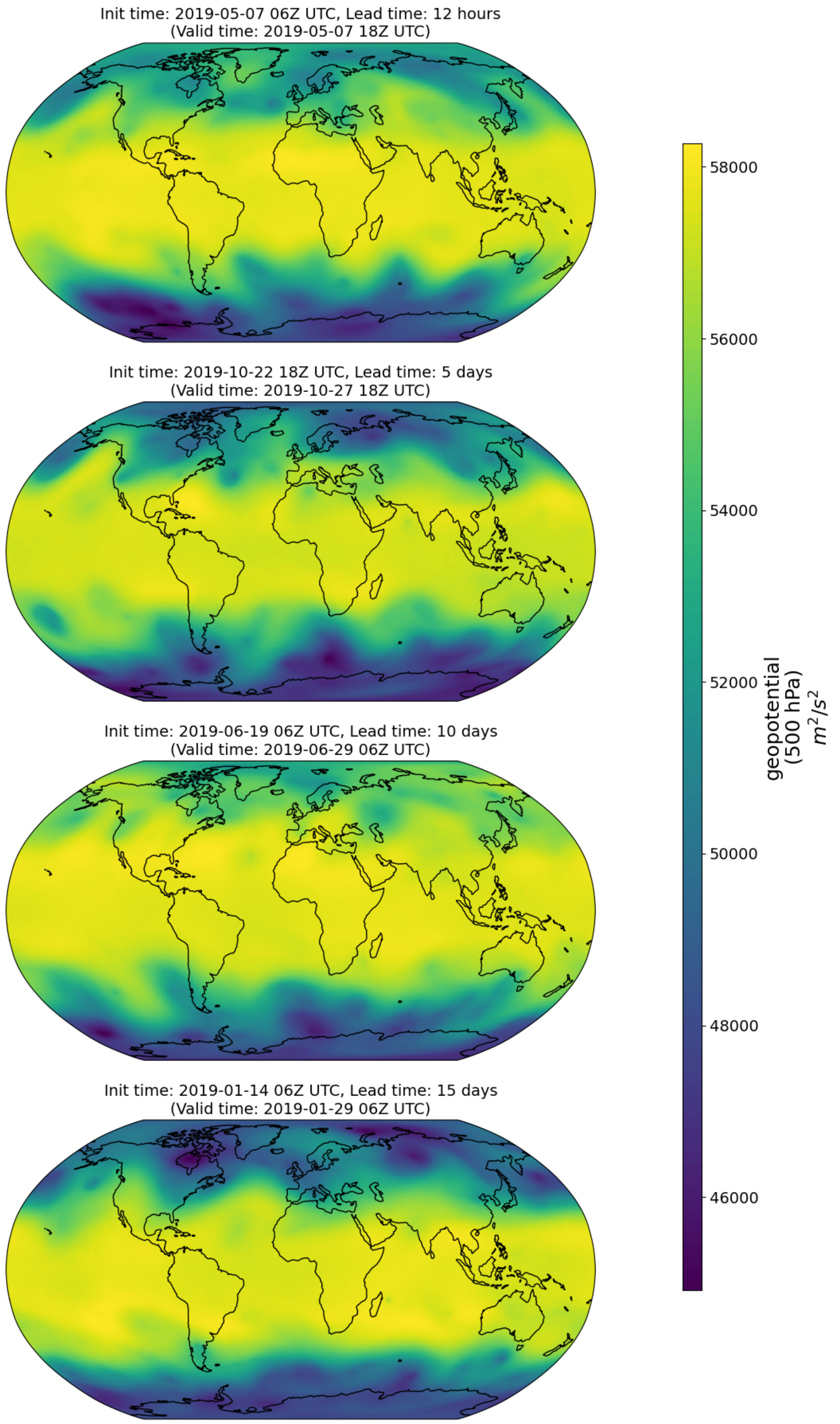

Figure C7: Visualisation of geopotential at 500 hPa.

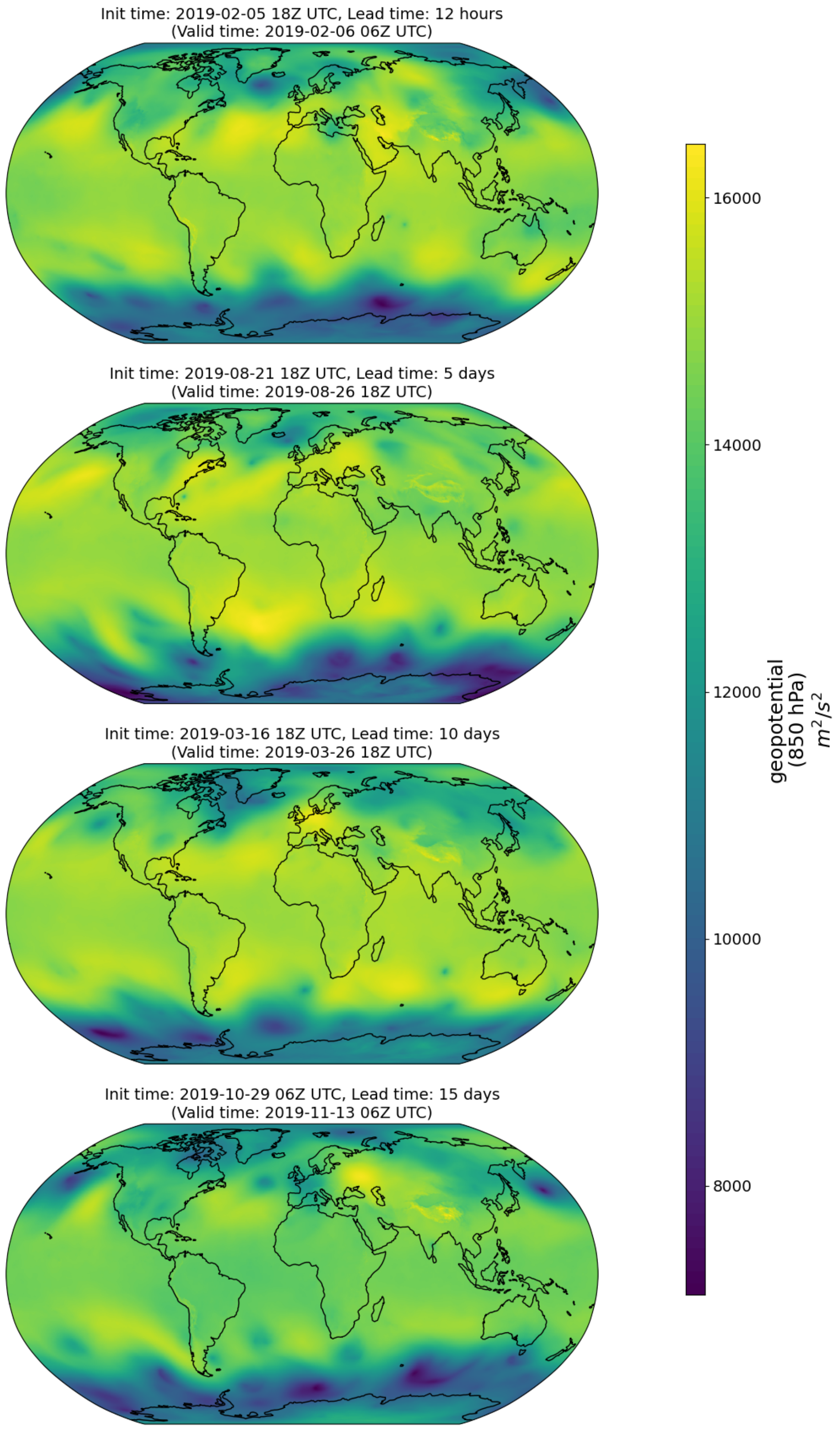

Figure C8: Visualisation of geopotential at 850 hPa.

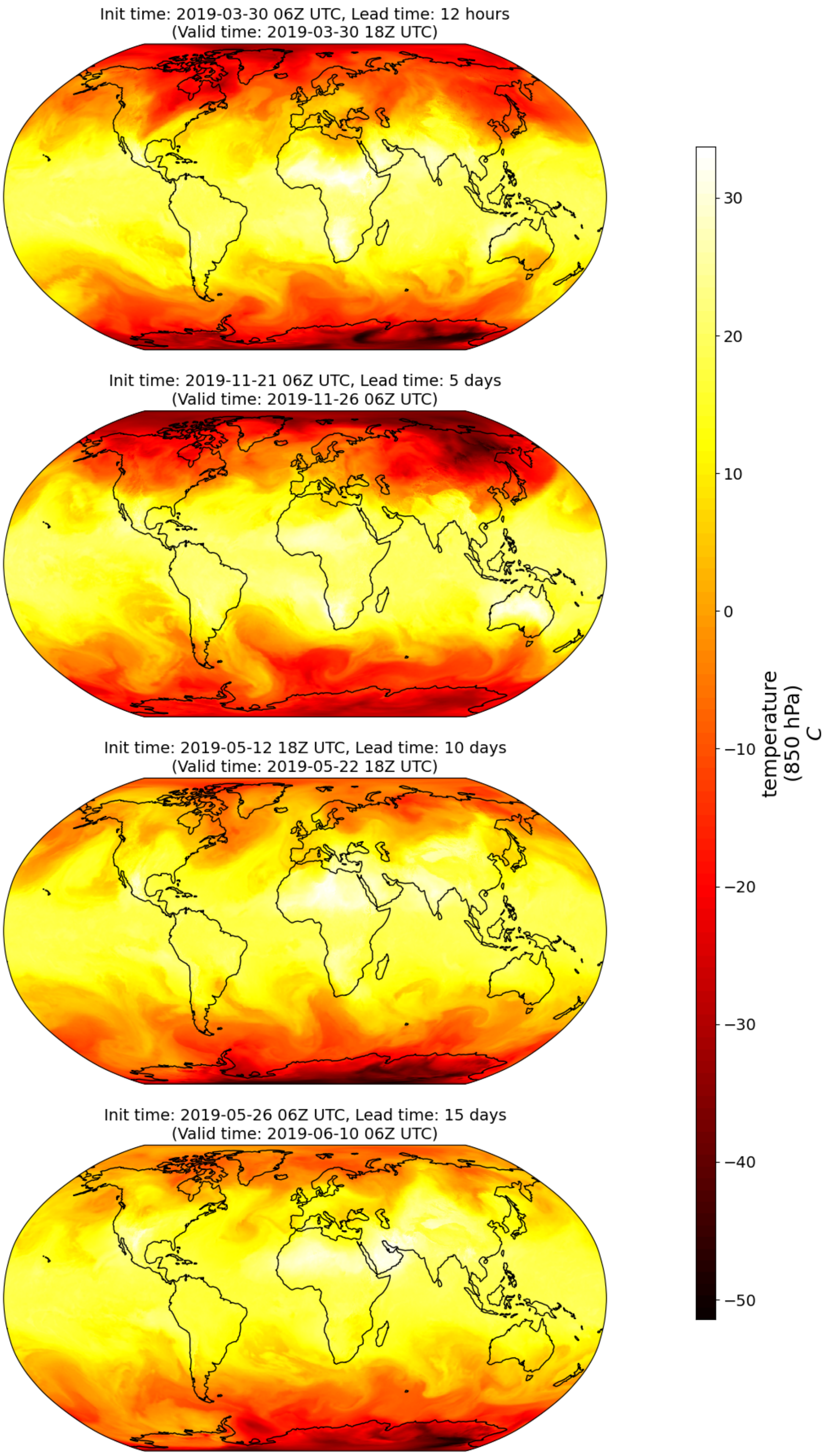

Figure C9: Visualisation of temperature at 850 hPa.

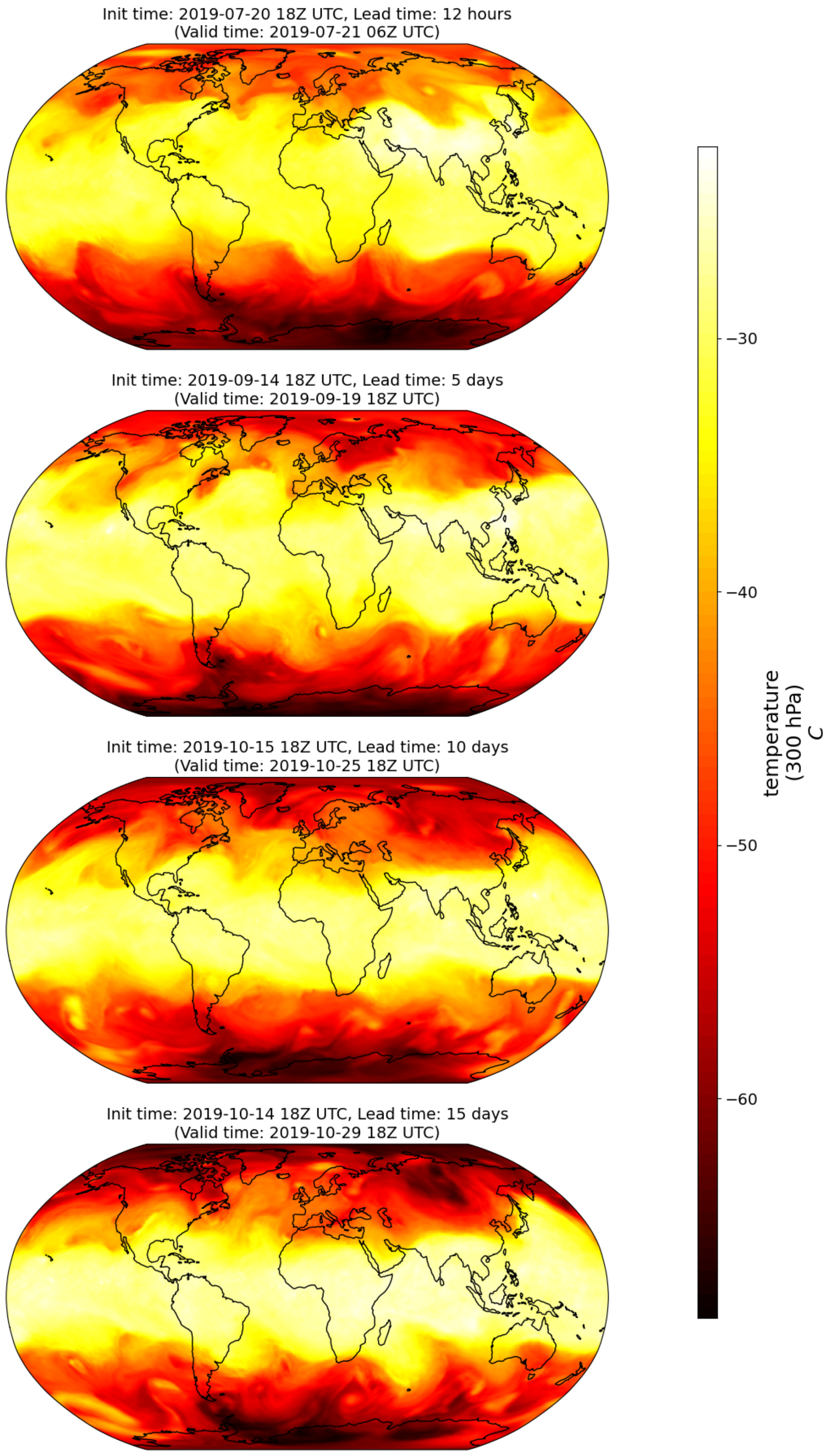

Figure C10: Visualisation of temperature at 300 hPa.

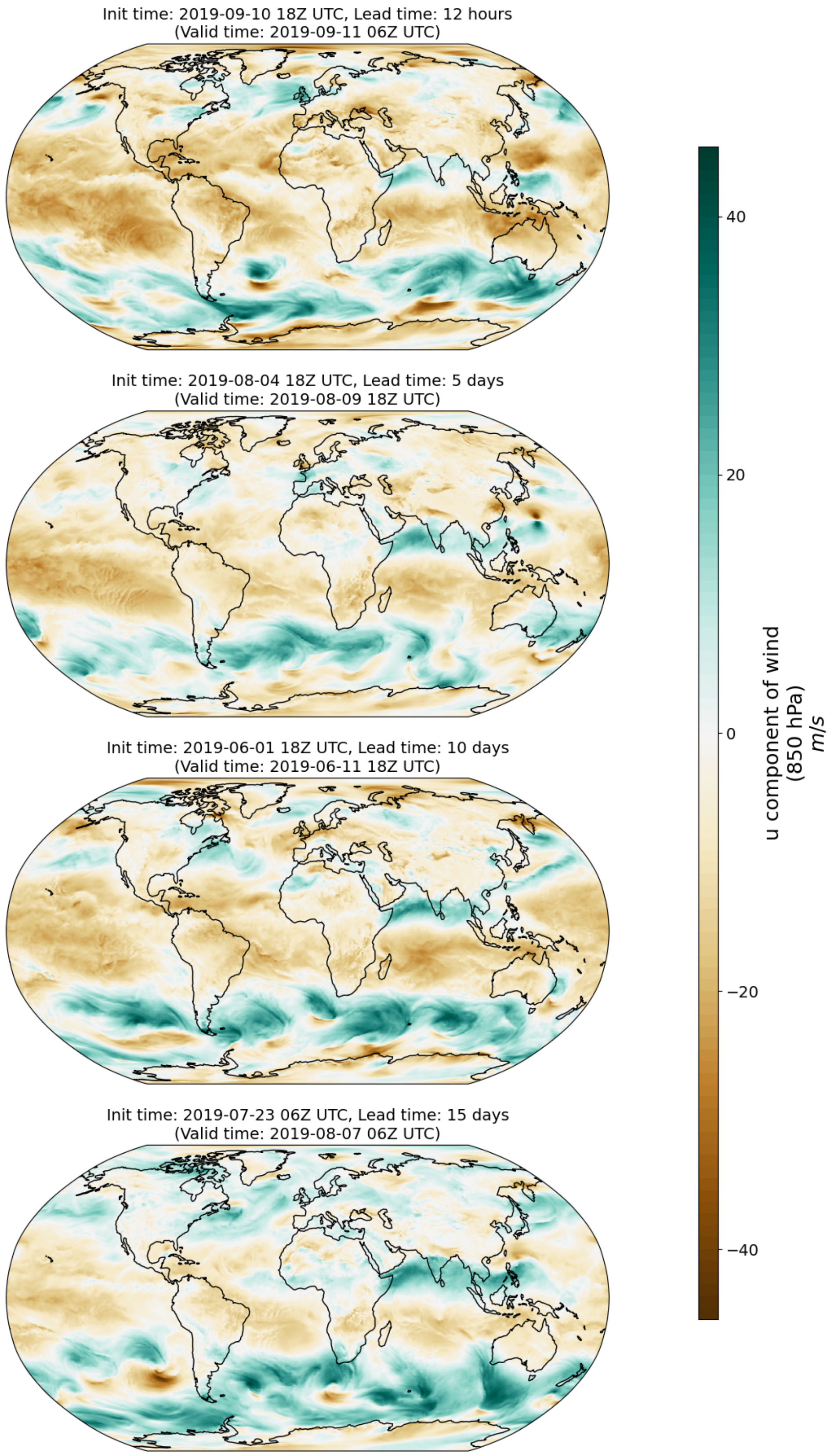

Figure C11: Visualisation of u component of wind at 850 hPa.

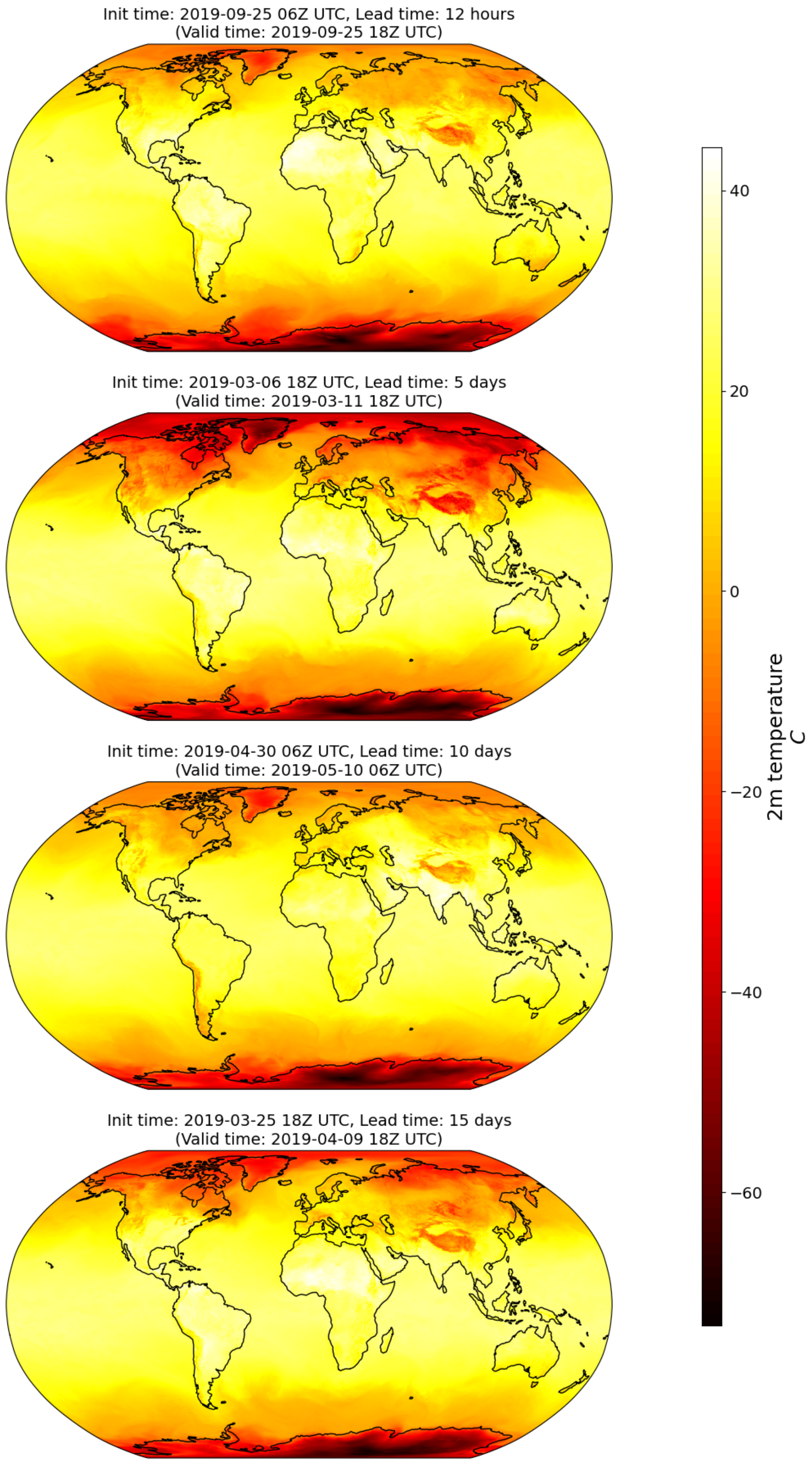

Figure C12: Visualisation of 2 meter temperature.

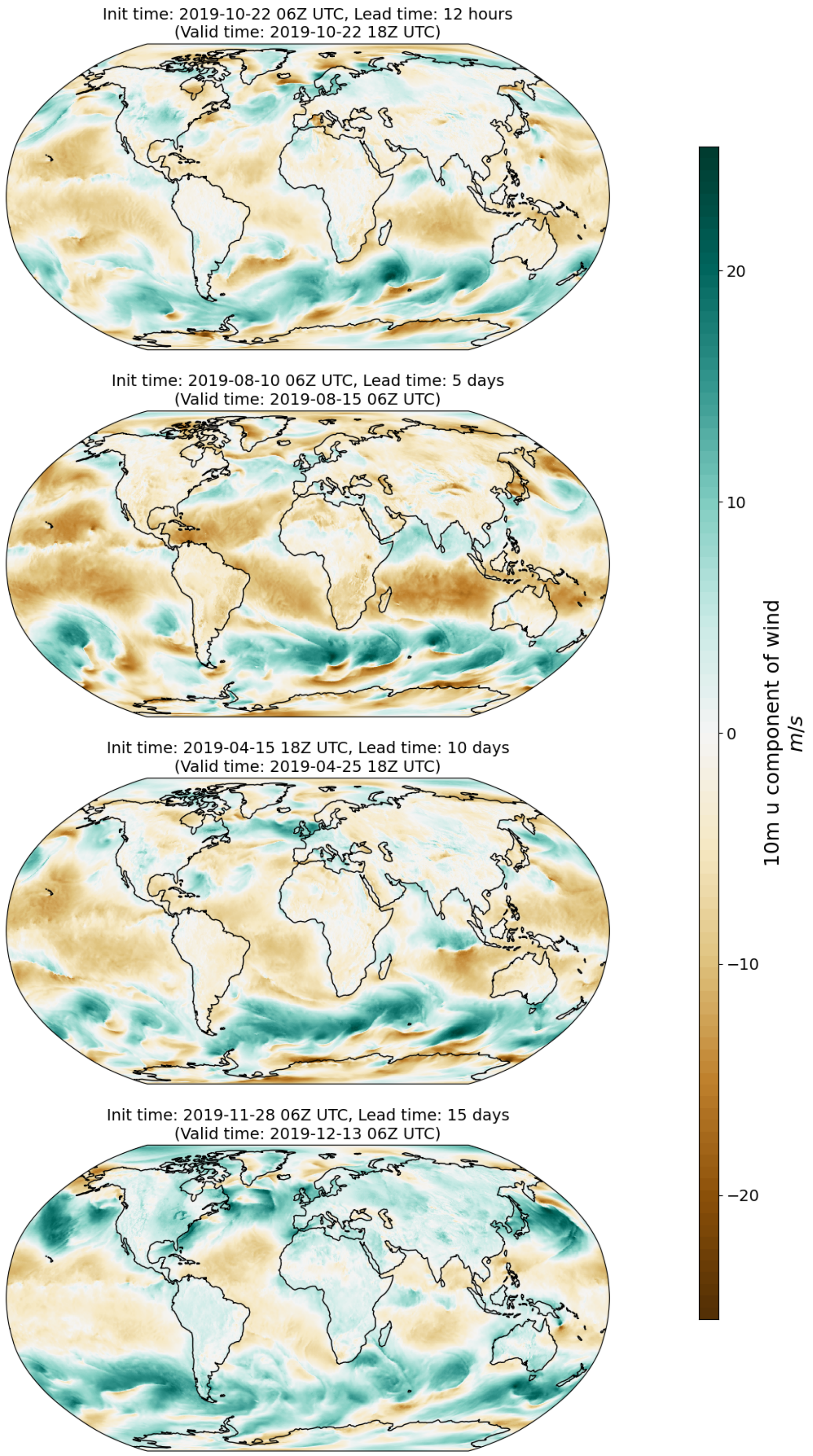

Figure C13: Visualisation of 10 meter u component of wind.

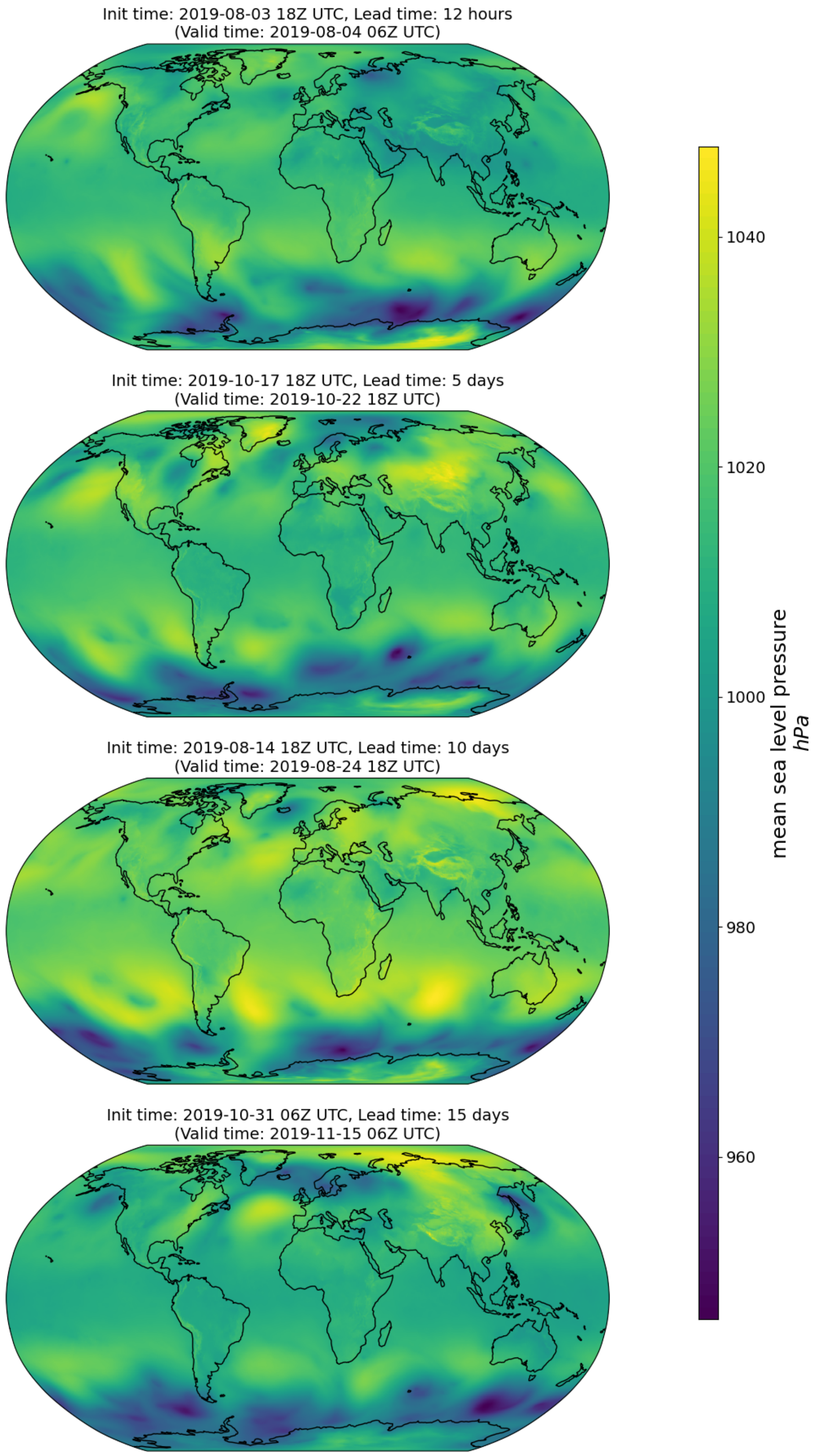

Figure C14: Visualisation of mean sea level pressure.

References

[1] Bauer, P., Thorpe, A. & Brunet, G. The quiet revolution of numerical weather prediction. *Nature* **525**, 47–55 (2015).

[2] Lam, R. *et al.* Learning skillful medium-range global weather forecasting. *Science* **382**, 1416–1421 (2023).

[3] Bi, K. *et al.* Accurate medium-range global weather forecasting with 3d neural networks. *Nature* **619**, 533–538 (2023).

[4] ECMWF. *IFS Documentation CY46R1*, chap. Part V: Ensemble Prediction System (ECMWF, 2019). URL https://www.ecmwf.int/node/19309.

[5] Lorenz, E. N. *The essence of chaos* (University of Washington Press, 1993).

[6] Palmer, T. & Hagedorn, R. *Predictability of weather and climate* (Cambridge University Press, 2006).

[7] Kalnay, E. *Atmospheric modeling, data assimilation and predictability* (Cambridge university press, 2003).

[8] Palmer, T. The ECMWF ensemble prediction system: Looking back (more than) 25 years and projecting forward 25 years. *Quarterly Journal of the Royal Meteorological Society* **145**, 12–24 (2019).

[9] Roberts, N. *et al.* Improver: The new probabilistic postprocessing system at the Met office. *Bulletin of the American Meteorological Society* **104**, E680–E697 (2023).

[10] Yamaguchi, H. *et al.* Introduction to JMA's new global ensemble prediction system. *CAS/JSC WGNE, Research Activities in Atmospheric and Oceanic Modelling* **42**, 6–13 (2018).

[11] Zhu, Y., Toth, Z., Wobus, R., Wei, M. & Cui, B. May 2006 upgrade of the GEFS and first implementation of NAEFS systems (2012b).

[12] ECMWF. Plans for high-resolution forecast (HRES) and ensemble forecast (ENS) control run. https://www.ecmwf.int/en/about/media-centre/focus/2024/plans-high-resolution-forecast-hres-and-ensemble-forecast-ens (2024). [Accessed 25-04-2024].

[13] Pathak, J. *et al.* Fourcastnet: A global data-driven high-resolution weather model using adaptive fourier neural operators. *arXiv preprint arXiv:2202.11214* (2022).

[14] Keisler, R. Forecasting global weather with graph neural networks. *arXiv preprint arXiv:2202.07575* (2022).

[15] Kurth, T. *et al.* FourCastNet: Accelerating global high-resolution weather forecasting using adaptive fourier neural operators. *arXiv preprint arXiv:2208.05419* (2022).

[16] Chen, K. *et al.* Fengwu: Pushing the skillful global medium-range weather forecast beyond 10 days lead. *arXiv preprint arXiv:2304.02948* (2023).

[17] Nguyen, T. *et al.* Scaling transformer neural networks for skillful and reliable medium-range weather forecasting. *arXiv preprint arXiv:2312.03876* (2023).

[18] Li, H. *et al.* FuXi: A cascade machine learning forecasting system for 15-day global weather forecast. *npj Climate and Atmospheric Science* **6** (2023).

[19] Graubner, A. *et al.* Calibration of large neural weather models. In *NeurIPS 2022 Workshop on Tackling Climate Change with Machine Learning* (2022).

[20] Kochkov, D. *et al.* Neural general circulation models for weather and climate. *Nature* **632**, 1060–1066 (2024).

[21] Karras, T., Aittala, M., Aila, T. & Laine, S. Elucidating the design space of diffusion-based generative models. *Advances in Neural Information Processing Systems* **35**, 26565–26577 (2022).

[22] Song, Y. *et al.* Score-based generative modeling through stochastic differential equations. In *International Conference on Learning Representations* (2021). URL https://openreview.net/forum?id=PxTIG12RRHS.

[23] Sohl-Dickstein, J., Weiss, E., Maheswaranathan, N. & Ganguli, S. Deep unsupervised learning using nonequilibrium thermodynamics. In *International conference on machine learning*, 2256–2265 (PMLR, 2015).

[24] Yang, R., Srivastava, P. & Mandt, S. Diffusion probabilistic modeling for video generation. *Entropy* **25**, 1469 (2023).

[25] Croitoru, F.-A., Hondru, V., Ionescu, R. T. & Shah, M. Diffusion models in vision: A survey. *IEEE Transactions on Pattern Analysis and Machine Intelligence* **45**, 10850–10869 (2023).

[26] Vaswani, A. *et al.* Attention is all you need. *Advances in neural information processing systems* **30** (2017).

[27] Hersbach, H. *et al.* The ERA5 global reanalysis. *Quarterly Journal of the Royal Meteorological Society* **146**, 1999–2049 (2020).

[28] Swinbank, R. *et al.* The tigge project and its achievements. *Bulletin of the American Meteorological Society* **97**, 49–67 (2016).

[29] W.M.O. *Manual on the Global Data-processing and Forecasting System* (World Meteorological Organization, 2023). URL https://library.wmo.int/idurl/4/35703.

[30] Rasp, S. *et al.* Weatherbench 2: A benchmark for the next generation of data-driven global weather models. *arXiv preprint arXiv:2308.15560* (2023).

[31] Gneiting, T. & Raftery, A. E. Strictly proper scoring rules, prediction, and estimation. *Journal of the American statistical Association* **102**, 359–378 (2007).

[32] Fortin, V., Abaza, M., Anctil, F. & Turcotte, R. Why should ensemble spread match the RMSE of the ensemble mean? *Journal of Hydrometeorology* **15**, 1708–1713 (2014).

[33] Talagrand, O. Evaluation of probabilistic prediction systems. In *Proceedings of "Workshop on Predictability", 20-22 October 1997, ECMWF, Reading, UK* (1999).

[34] Titley, H. A., Bowyer, R. L. & Cloke, H. L. A global evaluation of multi-model ensemble tropical cyclone track probability forecasts. *Quart. J. Roy. Meteor. Soc.* **146**, 531–545 (2020).

[35] Katz, R. W. & Murphy, A. H. *Economic value of weather and climate forecasts* (Cambridge University Press, 1997).

[36] Murphy, A. H. A note on the utility of probabilistic predictions and the probability score in the Cost-Loss ratio decision situation. *J. Appl. Meteorol. Climatol.* **5**, 534–537 (1966).

[37] Richardson, D. S. Skill and relative economic value of the ECMWF ensemble prediction system. *Quarterly Journal of the Royal Meteorological Society* **126**, 649–667 (2000).

[38] Richardson, D. S. *Predictability and economic value*, 628–644 (Cambridge University Press, 2006).

[39] Ebert, E. E. Fuzzy verification of high-resolution gridded forecasts: a review and proposed framework. *Meteorol. Appl.* **15**, 51–64 (2008).

[40] Siebert, N. *Development of methods for regional wind power forecasting.* Ph.D. thesis, École Nationale Supérieure des Mines de Paris (2008).

[41] Matos, M. A. & Bessa, R. J. Setting the operating reserve using probabilistic wind power forecasts. *IEEE Trans. Power Syst.* **26**, 594–603 (2011).

[42] Rachunok, B., Staid, A., Watson, J.-P. & Woodruff, D. L. Assessment of wind power scenario creation methods for stochastic power systems operations. *Appl. Energy* **268**, 114986 (2020).

[43] Gielen, D. *et al.* The role of renewable energy in the global energy transformation. *Energy Strategy Reviews* **24**, 38–50 (2019).

[44] Byers, L. *et al.* A global database of power plants. *World Resources Institute* (2018).

[45] Martinez, A. B. Forecast accuracy matters for hurricane damage. *Econometrics* **8**, 18 (2020).

[46] Dunion, J. P. *et al.* Recommendations for improved tropical cyclone formation and position probabilistic forecast products. *Tropical Cyclone Research and Review* **12**, 241–258 (2023).

[47] Ullrich, P. A. *et al.* TempestExtremes v2.1: a community framework for feature detection, tracking, and analysis in large datasets. *Geosci. Model Dev.* **14**, 5023–5048 (2021).

[48] Magnusson, L. *et al.* Tropical cyclone activities at ECMWF. *ECMWF Technical Memorandum* (2021).

[49] Salimans, T. & Ho, J. Progressive distillation for fast sampling of diffusion models. In *International Conference on Learning Representations* (2022). URL https://openreview.net/forum?id=TIdIXIpzhoI.

[50] Huang, L., Gianinazzi, L., Yu, Y., Dueben, P. D. & Hoefler, T. DiffDA: a diffusion model for weather-scale data assimilation. In *Forty-first International Conference on Machine Learning* (2024). URL https://openreview.net/forum?id=vhMq3eAB34.

[51] Li, L., Carver, R., Lopez-Gomez, I., Sha, F. & Anderson, J. Generative emulation of weather forecast ensembles with diffusion models. *Science Advances* **10**, eadk4489 (2024).

[52] Addison, H., Kendon, E., Ravuri, S., Aitchison, L. & Watson, P. Machine learning emulation of a local-scale uk climate model. In *NeurIPS 2022 Workshop on Tackling Climate Change with Machine Learning* (2022). URL https://www.climatechange.ai/papers/neurips2022/21.

[53] Lu, C. *et al.* DPM-Solver++: Fast solver for guided sampling of diffusion probabilistic models. *arXiv preprint arXiv:2211.01095* (2022).

[54] Batzolis, G., Stanczuk, J., Schönlieb, C.-B. & Etmann, C. Conditional image generation with score-based diffusion models. *arXiv preprint arXiv:2111.13606* (2021).

[55] Ho, J., Jain, A. & Abbeel, P. Denoising diffusion probabilistic models. *Advances in neural information processing systems* **33**, 6840–6851 (2020).

[56] Nguyen, T. Q. & Salazar, J. Transformers without tears: Improving the normalization of self-attention. In *Proceedings of the 16th International Conference on Spoken Language Translation* (2019).

[57] Chen, M. *et al.* Adaspeech: Adaptive text to speech for custom voice. In *International Conference on Learning Representations* (2021). URL https://openreview.net/forum?id=Drynvt7gg4L.

[58] Politis, D. N. & Romano, J. P. The stationary bootstrap. *Journal of the American Statistical association* **89**, 1303–1313 (1994).

[59] Politis, D. N. & White, H. Automatic block-length selection for the dependent bootstrap. *Econometric reviews* **23**, 53–70 (2004).

[60] Patton, A., Politis, D. N. & White, H. Correction to "Automatic block-length selection for the dependent bootstrap" by D. Politis and H. White. *Econometric Reviews* **28**, 372–375 (2009).

[61] Davison, A. C. & Hinkley, D. V. *Bootstrap Methods and their Application* (Cambridge University Press, 1997).

[62] Efron, B. & Narasimhan, B. The automatic construction of bootstrap confidence intervals. *Journal of Computational and Graphical Statistics* **29**, 608–619 (2020).

[63] Knapp, K. R., Kruk, M. C., Levinson, D. H., Diamond, H. J. & Neumann, C. J. The international best track archive for climate stewardship (IBTrACS): Unifying tropical cyclone data. *Bull. Am. Meteorol. Soc.* **91**, 363–376 (2010).

[64] Knapp, K. R. *et al.* International best track archive for climate stewardship (ibtracs) project, version 4. *NOAA National Centers for Environmental Information* **10** (2018).

[65] Ullrich, P. A. & Zarzycki, C. M. TempestExtremes: a framework for scale-insensitive pointwise feature tracking on unstructured grids. *Geosci. Model Dev.* **10**, 1069–1090 (2017).

[66] Zarzycki, C. M. & Ullrich, P. A. Assessing sensitivities in algorithmic detection of tropical cyclones in climate data. *Geophys. Res. Lett.* **44**, 1141–1149 (2017).

[67] King, J., Clifton, A. & Hodge, B. M. Validation of power output for the wind toolkit. *National Renewable Energy Laboratory, NREL* (2014). URL https://www.osti.gov/biblio/1159354.

[68] Lean, P., Bonavita, M., Hólm, E., Bormann, N. & McNally, T. Continuous data assimilation for the IFS. *ECMWF Newsletter* 21–26 (2019). URL https://www.ecmwf.int/node/18882.

[69] Hunter, J. D. Matplotlib: A 2d graphics environment. *Computing in Science & Engineering* **9**, 90–95 (2007).

[70] Met Office. *Cartopy: a cartographic python library with a Matplotlib interface*. Exeter, Devon (2010 - 2015). URL https://scitools.org.uk/cartopy.

[71] Rasp, S. *et al.* WeatherBench: a benchmark data set for data-driven weather forecasting. *Journal of Advances in Modeling Earth Systems* **12** (2020).

[72] Driscoll, J. R. & Healy, D. M. Computing fourier transforms and convolutions on the 2-sphere. *Adv. Appl. Math.* **15**, 202–250 (1994).

[73] Loshchilov, I. & Hutter, F. Decoupled weight decay regularization. In *International Conference on Learning Representations* (2018).

[74] Weaver, A. & Courtier, P. Correlation modelling on the sphere using a generalized diffusion equation. *Quarterly Journal of the Royal Meteorological Society* **127**, 1815–1846 (2001).

[75] Zamo, M. & Naveau, P. Estimation of the continuous ranked probability score with limited information and applications to ensemble weather forecasts. *Mathematical Geosciences* **50**, 209–234 (2018).

[76] Ferro, C. A. T. Fair scores for ensemble forecasts. *Quart. J. Roy. Meteor. Soc.* **140**, 1917–1923 (2014).

[77] Hamill, T. M. Interpretation of rank histograms for verifying ensemble forecasts. *Monthly Weather Review* **129**, 550–560 (2001).

[78] Wilks, D. On the reliability of the rank histogram. *Monthly Weather Review* **139**, 311–316 (2011).

[79] Brier, G. W. Verification of forecasts expressed in terms of probability. *Monthly weather review* **78**, 1–3 (1950).

[80] Ferro, C. A. T. Comparing probabilistic forecasting systems with the brier score. *Weather Forecast.* **22**, 1076–1088 (2007).

[81] Wilks, D. A skill score based on economic value for probability forecasts. *Meteorological Applications* **8**, 209–219 (2001).

[82] Thompson, J. On the operational deficiences in categorical weather forecasts. *Bulletin of the American Meteorological Society* **33**, 223–226 (1952).

[83] Murphy, A. H. The value of climatological, categorical and probabilistic forecasts in the cost-loss ratio situation. *Monthly Weather Review* **105**, 803–816 (1977).

[84] Sheppard, K. *et al.* bashtage/arch: Release 7.0.0 (2024). URL https://doi.org/10.5281/zenodo.10981635.

[85] Conroy, A. *et al.* Track forecast: Operational capability and new techniques - summary from the tenth international workshop on tropical cyclones (IWTC-10). *Tropical Cyclone Research and Review* **12**, 64–80 (2023).

[86] Gilleland, E., Ahijevych, D., Brown, B. G., Casati, B. & Ebert, E. E. Intercomparison of spatial forecast verification methods. *Weather Forecast.* **24**, 1416–1430 (2009).

[87] Ravuri, S. *et al.* Skilful precipitation nowcasting using deep generative models of radar. *Nature* **597**, 672–677 (2021).

[88] Drew, D. R., Cannon, D. J., Barlow, J. F., Coker, P. J. & Frame, T. H. A. The importance of forecasting regional wind power ramping: A case study for the UK. *Renewable Energy* **114**, 1201–1208 (2017).

[89] Rodwell, M. J., Richardson, D. S., Hewson, T. D. & Haiden, T. A new equitable score suitable for verifying precipitation in numerical weather prediction. *Quarterly Journal of the Royal Meteorological Society* **136**, 1344–1363 (2010).

[90] Haiden, T. *et al.* Intercomparison of global model precipitation forecast skill in 2010/11 using the seeps score. *Monthly Weather Review* **140**, 2720–2733 (2012).

[91] North, R., Trueman, M., Mittermaier, M. & Rodwell, M. J. An assessment of the SEEPS and SEDI metrics for the verification of 6 h forecast precipitation accumulations. *Meteorological Applications* **20**, 164–175 (2013).
